# Protein profile of purified plasma- and aqueous humor-derived extracellular vesicles from patients with ocular toxoplasmosis

**DOI:** 10.5935/0004-2749.2023-0037

**Published:** 2024-07-09

**Authors:** Deise F. Costa, Carmen Luz Pessuti, Thupten Tsering, Mohamed Abdouh, Kleber Ribeiro, Heloisa Nascimento, Alessandra G. Commodaro, Julia V. Burnier, Rubens Belfort Jr, Miguel N. Burnier Jr

**Affiliations:** 1 MUHC-McGill University Ocular Pathology & Translational Research Laboratory Montreal, Canada; 2 Department of Ophthalmology, Universidade Federal de São Paulo, São Paulo, SP, Brazil; 3 Cancer Research Program, Research Institute of McGill University Health Centre, Montreal, Canada

**Keywords:** Extracellular vesicles, Proteomics, *Toxoplasma gondii*, Ocular toxoplasmosis, Aqueous humor, Plasma, Liquid biopsy

## Abstract

**Purpose:**

To characterize the extracellular vesicle protein cargo in the aqueous humor and plasma of patients with ocular toxoplasmosis.

**Methods:**

Aqueous humor and plasma were collected from six patients with active ocular toxoplasmosis and six patients with cataract. Extracellular vesicles were isolated, and western blotting and mass spectrometry were performed for protein analysis.

**Results:**

All plasma samples from patients with ocular toxoplasmosis and cataract were positive for the tetraspanins CD63 and TSG101. However, the aqueous humor from patients with ocular toxoplasmosis was positive only for CD63. Sixty-seven new unreported proteins were identified in the aqueous humor and plasma of patients with the ocular toxoplasmosis and cataract. Of the 67 proteins, 10 and 7 were found only in the cataract and ocular toxoplasmosis groups, respectively. In general, these proteins were involved in immune system activation and retina homeostasis and were related to infections and retina-associated diseases.

**Conclusion:**

The distinct protein signatures between ocular toxoplasmosis and cataract may be helpful in the differential diagnosis of ocular toxoplasmosis. However, more studies are needed to better understand the role of these proteins in the pathogenesis of ocular toxoplasmosis.

## INTRODUCTION

Ocular toxoplasmosis (OT) is caused by the protozoan parasite *Toxoplasma gondii* (*T. gondii*), and it can be congenital or acquired postnatally^(1)^. The parasite infects approximately 25%-30% of the human population worldwide^(2)^. However, the seroprevalence varies between different geographic areas and countries^(3)^. In Brazil, up to 50% of elementary school children and 50%–80% of women of childbearing age demonstrate antibodies against *T. gondii*. Thus, the seroprevalence in Brazil is four times higher than that in USA^(4)^.

Ocular manifestations of OT include floaters and blurred vision. Additionally, decreased visual acuity occur because of macular involvement, optic atrophy, or severe vitreous inflammation^(5)^.

Accurate diagnosis depends on the clinical features of the disease. However, atypical presentations and resemblance to other lesions (e.g., fungal retinitis, septic retinitis, and ocular toxocariasis) are sources of diagnostic challenges, which often lead to misdiagnosis and inappropriate treatment^(5)^.

Similar to several cell types, parasites release extracellular vesicles (EVs) that are postulated to be involved in cell-cell communication or in the modulation of host immune responses^(6)^. The EVs typically consist of a lipid bilayer membrane, containing integral membrane proteins, and a luminal cavity loaded with a variety of soluble proteins and nucleic acids (RNA and DNA)^(6)^. The EVs present a certain set of molecules that include proteins such as tumor susceptibility gene 101 (TGS101), CD9, and CD63; some of these proteins are considered essential components of the EVs. These EV marker proteins have been used to confirm the presence of EVs via immunoblotting^(7)^. Additionally, EVs serve as biomarkers for various diseases, including melanoma^(8)^ and epithelial ovarium cancer^(9)^.

Exosomes have been identified in aqueous humor (AH)^(10)^ and vitreous samples^(11)^. A study revealed that specific proteins were elevated in the AH of individuals with age-related macular degeneration (AMD). These results indicate that exosomal proteins in AH may be used as biomarkers for the diagnosis of AMD^(12)^.

EVs isolated from biological fluids could be a useful tool for the early diagnosis and treatment of OT. Thus, herein, we aimed to characterize the EV protein cargo in the AH and plasma of patients with OT.

## METHODS

### Patients

Six patients with active OT and six patients with a cataract (CAT) were recruited from March to July 2018 at the Department of Ophthalmology, UNIFESP/EPM, Brazil and Vision Institute, IPEPO, Brazil. Six patients diagnosed with active OT (4 women and 2 men; average age, 39 years) who tested seropositive for *T. gondii* underwent an ocular examination, including measurement of best-corrected visual acuity, applanation tonometry, undilated and dilated slit-lamp biomicroscopy, and indirect fundus examination. All six patients demonstrated more than 1+ cells in the anterior chamber, unilateral (n=5) or bilateral (n=1) involvement, recurrence (n=4) or no recurrence (n=2), and OT lesions characterized by typical focal necrotizing retinochoroiditis (Table 1). The six patients with a CAT who underwent routine cataract surgery (2 women and 4 men; average age, 68 years) were included as controls.

**Table 1:** Characteristics of OT-affected patients in this study

Patient ID	Age (y)	Sex	Affected side	Clinical occurrences	Vitreitis	ACR	Samples included in proteomic analyses	New proteins identified in the OT group only/patient and sample	
								AH	Plasma
Patient 1*	61	Male	Unilateral	Recurrent OT	-	1+/4+	Plasma only		ACAN
Patient 2*	30	Female	Bilateral	Recurrent OT	-	2+/4+	Plasma only		ACAN, ARHGAP45, ERBIN, and IGLV9-49
Patient 3*	36	Male	Unilateral	No recurrent OT	3+/4+	4+/4+	AH only	ADAMDEC1, FCRL5	
Patient 4*	27	Female	Unilateral	Recurrent OT	2+/4+	1+/4+	Plasma + AH	ND	ACAN, ARHGAP45
Patient 5*	51	Female	Unilateral	No recurrent OT	-	1+/4+	Plasma + AH	ND	GUCY1B1, ARHGAP45, and ERBIN
Patient 6*	29	Female	Unilateral	Recurrent OT	2+/4+	1+/4+	Plasma only		ARHGAP45, ERBIN

ND= not detected; OT= ocular toxoplasmosis; ACR= anterior chamber reaction; AH= aqueous humor.

* Patient 1= OT RC; Patient 2= OT VO; Patient 3= OT JC; Patient 4= OT JHC; Patient 5= OT JAF; Patient 6= OT JM.

This study was approved by the institutional ethics committee (No: 2198149) and was conducted in adherence to the principles of the Declaration of Helsinki and Resolution 196/96 of the Ministry of Health, Brazil. Informed consent was obtained from all the participants.

### Aqueous humor and plasma sample collection

Eleven peripheral blood and 12 AH samples were collected from the 12 patients. Peripheral blood (10 mL) was collected in EDTA tubes and centrifuged for 10 min at 1900 × g. Subsequently, 1 mL aliquots of plasma were collected. Anterior chamber paracentesis was performed using a slit lamp after application of a topical anesthetic and topical antiseptic. The ophthalmologist used a 1 mL tuberculin syringe (27-gauge ½-inch needle) to aspirate 0.1 mL of the AH. All samples were stored at 4°C until the assays were performed.

### EV isolation

The EVs were isolated according to the guidelines of the International Society for Extracellular Vesicles (ISEV-2018). The samples (1 mL plasma or 100 µl AH) were centrifuged at 16,000 × g for 10 min at 4°C to eliminate cellular debris. Thereafter, the EVs were isolated using the exoEasy Maxi Kit (Qiagen, Valencia, CA, USA) according to the manufacturer’s instructions. The samples were gently mixed with the same volume of buffer XBP. The mixture was charged onto the exoEasy spin column and centrifuged at 500 × g for 1 min. After washing the column with buffer XWP, the EVs were eluted in 250 µl of buffer XE by centrifugation at 500 × g for 5 min.

### EV characterization

Aliquots of isolated EVs were diluted 100 times in PBS and processed using a nanoparticle tracking analysis (NTA) system and the NanosightNS300 (Malvern, UK). The samples were read in triplicate for 30 s at 20 frames/s. NTA (version 3.2) was used to estimate the concentration and size of the EV particles isolated from both the plasma and AH.

The EVs were lysed in RIPA buffer containing complete mini protease inhibitors (Sigma) at 4°C for 30 min. The samples were pulse sonicated for 2 s (3 times) and centrifuged at 13,000 x g for 30 min at 4°C. Protein concentrations were quantified using the BCA assay (Thermo Fisher Scientific) according to the manufacturer’s instructions. Protein samples were processed for western blotting and mass spectrometry (MS) proteomic analysis.

### Western blotting

Aliquots of 20 µg of EV proteins were separated using 12% precast polyacrylamide gel (Mini-PROTEAN; BioRad) in running buffer at 120 V for 70 min. The proteins were electrotransferred onto polyvinylidene difluoride (PVDF) membranes using Trans-Bot Turbo transfer (Biorad). Subsequently, the PVDF membranes were blocked for 1 h at room temperature with 5% non-fat dry milk in 1X Tris-buffer saline with 0.05% Tween 20. The membranes were probed with primary antibodies against TSG101, CD63 (Abcam 1:1000), or GM-130 (Abcam 1:1000), followed by HRP-conjugated secondary antibodies [goat anti-rabbit (Sigma 1:1000) or goat anti-mouse (Sigma 1:3000)]. The membranes were washed five times for 10 min each after each incubation, developed using the ECL prime Western blot detection reagent (GE healthcare), and visualized using the ChemiDoc^TM^ XRS+ System.

### Transmission electron microscopy

Carbon-coated copper transmission electron microscopy (TEM) grids were negatively charged using PELCO easiGlow, and 20 µl of the EV sample was layered on it for 20 min. Following sample adsorption, the grids were quickly and gently washed with milliQ water for 10 min. Excess water was removed using a filter paper. It was immediately negatively stained with 2% uranyl acetate for 4 min, and dried on the filter paper. Imaging was performed using a 120 kV Cryo- transmission electron microsco pe (FEI Tecnai G2 Spirit Twin; ) and a camera system (Ultrascan 4000 4k × 4k CCD Model 895; Gatan).

### Label-free liquid chromatography-MS/MS proteomics analysis of EVs and database search

Aliquots of 20 µg of EV proteins from each sample were loaded onto a single stacking gel band to eliminate contaminants such as lipids, detergents, and salts. Each sample was run in duplicate.

The gel band was reduced with dithiothreitol (DTT), alkylated with iodoacetic acid, and digested with trypsin. The extracted peptides were resolubilized in 0.1% aqueous formic acid and loaded onto an Acclaim Pep-Map (75 µm inner diameter × 2 cm, C18 3 µm particle size; Thermo Scientific) precolumn. Subsequently, they were loaded onto an Acclaim PepMap EASY-Spray (75 µm inner diameter × 15 cm, 2 µm C18, 2 µm beads) analytical separation column using a Dionex UltiMate 3000 uHPLC at 250 nL/min, with a gradient of 2%-35% organic (0.1% formic acid in acetonitrile) over 3 h. The peptides were analyzed using an Orbitrap Fusion MS (Thermo) operating at 120,000 resolution (full width at half maximum in MS1) with higher-energy collisional dissociation sequencing (15,000 resolution) at top speed for all peptides and a charge of ≥2+. The MS raw data were converted into *.mgf format (Mascot generic format) and searched using the Mascot search engine (version 2.6.2; Matrix Science) against human protein sequences (Uniprot 2019). The database search results were loaded onto Scaffold Q+ Scaffold (version 4.10.0; Proteome Sciences) for spectral counting, statistical treat ment, data visualization, and quantification. Protein threshold >99%, peptide threshold >95%, and two of a minimum number of unique peptides were applied in Scaffold Q+ to increase the confidence level of identified proteins. Additional filters such as a p-value of <0.05 and a fold-value change of ≥2 were used to identify the differential expression of proteins. The identified protein list in Scaffold was exported to Microsoft Excel and uploaded into the DAVID bioinformatics database (version 6.8) for gene ontology analyses (i.e., biological process, cellular component, and KEGG pathway). Additionally, bioinformatic analysis and a Vesiclepedia database search were performed using FunRich (version 3.1.3).

The MS proteomics data have been deposited in the ProteomeXchange Consortium via the PRIDE partner repository (dataset identifier: PXD046167).

## RESULTS

### Proteomic analysis of EVs isolated from plasma and AH samples

**Figure 1:**
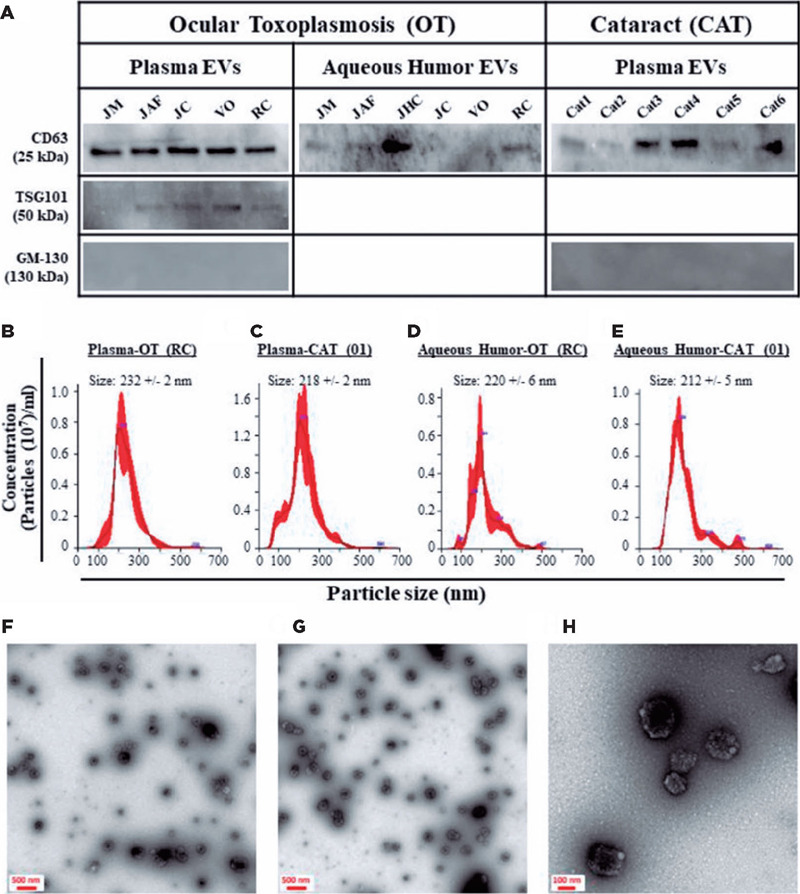
Particles isolated from the plasma and AH of patients with OT and CAT expressed markers of EV and displayed the physical characteristics of EV. (A) Proteins isolated from the plasma- and AH-derived EVs were analyzed by western blot for the expression of specific markers (i.e., CD63 and TSG101). Note: the EV preparations are pure as they did not express markers of other cellular organelles. (B-E) Nanosight analyses of EVs. Representative size distribution histograms showing data of EVs isolated from the plasma of patients with (B) OT and (C) CAT and of EVs isolated from the AH of patients with (D) OT and (E) CAT. Note: the mean EV sizes are identical between the analyzed samples. (F–H) Representative micrographs of TEM analyses demonstrate small vesicles (approximately 100–250 nm in diameter) isolated from the plasma of patients with (F) CAT and (G) OT. (H) 30000 x magnification view.

All five plasma-derived EV samples from the patients with OT were positive for TSG101 and CD63 markers; however, 4 of 6 AH-derived EV samples were positive only for CD63 (Figure 1A). All six plasma-derived EV samples from the patients with CAT were positive for the CD63 marker; however, all the AH-derived EV samples were negative for both markers (Figure 1A). All the analyzed EV samples were negative for GM-130 (a negative EV marker), indicating that the EV preparations were pure and not contaminated with other cellular organelles (Figure 1A). To obtain more insight into the identity of the isolated particles, we physically analyzed them using NTA and TEM. The isolated EVs demonstrated a standard distribution pattern when analyzed by NTA (Figure 1B,1C,1D,E). They had a mean size of 204–290 nm, with no difference based on their origin (plasma vs. AH) or patient’s health status (OT vs. CAT). TEM analyses demonstrated that the isolated particles were rounded structures ranging from 100 to 250 nm in size (Figure 1F,G,H). These findings are similar to those of our previous studies^(13,14)^, indicating that the analyzed particles demonstrated the phenotypic and physical characteristics of EVs.

**Figure 2:**
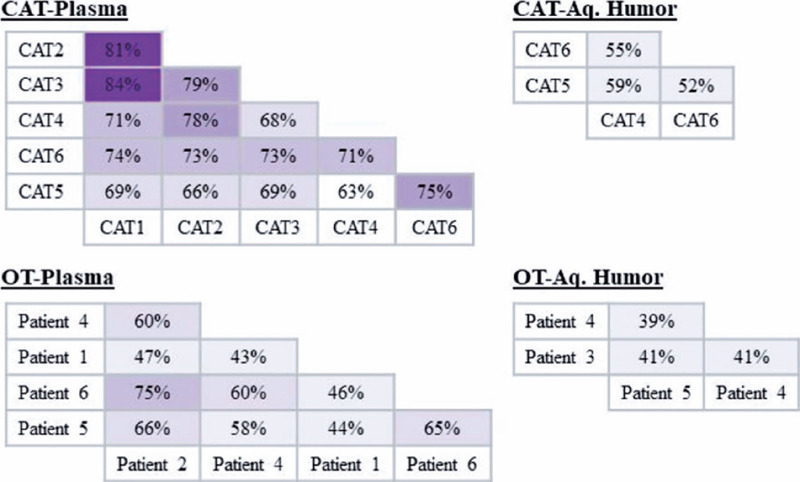
Protein cargo of plasma-derived EV is similar to that of AH-derived EV. Venn diagram analyses was performed using FUNRICH. Analyses of the EV protein cargo per patients’ groups (six CAT plasma, five OT plasma, three CAT AH, and three OT AH samples) is depicted. The protein similarities in the EVs from the same group of donors vary from 39% to 84% (i.e., percentage of overlapping). The different shades of purple reflect the degree of similarity between the corresponding samples: clear purple reflects less similarity and dark purple reflects high similarity.

For protein profile analysis, the EVs were isolated in patients with OT (AH, n=3; plasma n=5) and patients with CAT (AH, n=3; plasma n=6) (Table 1). In the OT group, protein similarities in EVs varied from 43% (Patient 1 vs. Patient 4) to 75% (Patient 6 vs. Patient 2) in the plasma and from 39% (Patient 4 vs. Patient 5) to 41% (Patient 3 vs. Patient 5 and Patient 3 vs. Patient 4) in the AH. In the control group, similarities ranged from 63% (CAT 5 vs. CAT 4) to 84% (CAT 3 vs. CAT 1) in the plasma and from 52% (CAT 5 vs. CAT 6) to 59% (CAT 5 vs. CAT 4) in AH (Figure 2). These results indicate that the protein cargo from EVs was consistent among the samples analyzed in this study.

**Figure 3:**
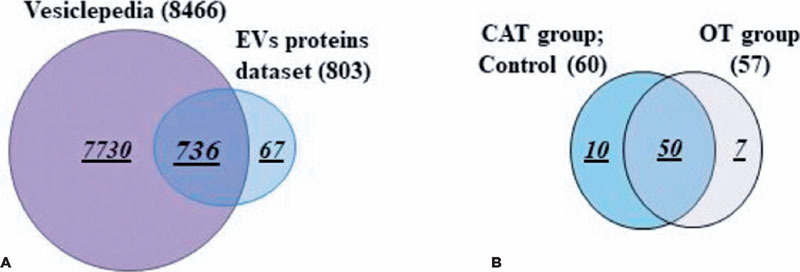
Proteomic minning of the EV cargo revealed the presence of new proteins. (A) Venn diagram analyses demonstrate that the majority (90–93%) of the proteins isolated from EVs derived from diferent samples were published in the Vesiclepedia database. Note: Sixty-seven proteins were not previously reported. (B) Venn diagram showing the distribution of the newly reported EV proteins in the different groups (CAT vs. OT).

A total of 803 proteins were identified in the AH and plasma from both the OT and CAT groups (Table 2). Among these, 736 (92%) had already been reported in the Vesiclepedia database, as determined by the FunRich bioinformatics analysis (Figure 3A).

**Table 2:** Proteins isolated from the plasma- and aqueous humor-derived EVs

#	Identified Proteins	Alternative Name	MW	Aqueous Humour (AH)												Plasma (P)																					
				Cataract (CAT, control)						Ocular Toxoplasmosis (OT)						Cataract (CAT, control)												Ocular Toxoplasmosis (OT)									
				7 1	7 2	JJ_1	JJ_2	MS 1	MS 2	JC_1	JC_2	JHC_1	JHC_2	VO_1	VO_2	01_1	01_2	02_1	02_2	04_1	04_2	7_1	7_2	JJ_1	JJ_2	MS_1	MS_2	JAF_1	JAF_2	JC_1	JC_2	JM_1	JM_2	RC_1	RC_2	VO_1	VO_2
1	Serum albumin OS=Homo sapiens OX=9606 GN=ALB PE=1 SV=2	ALB	69 kDa	458	458	689	651	290	347	271	368	662	622	187	237	1913	1354	881	887	1390	1505	685	714	580	630	688	696	1272	1227	486	462	1652	1509	878	854	598	507
2	Inter-alpha-trypsin inhibitor heavy chain H1 OS=Homo sapiens OX=9606 GN=ITIH1 PE=1 SV=3	ITIH1	101 kDa	26	26	71	64	15	15	20	1	52	62	5	20	811	528	695	799	1055	1008	345	446	609	704	473	417	886	1033	241	212	555	558	502	441	549	387
3	Inter-alpha-trypsin inhibitor heavy chain H2 OS=Homo sapiens OX=9606 GN=ITIH2 PE=1 SV=2	ITIH2	106 kDa	34	34	110	93	22	23	25	2	106	108	20	44	738	570	727	781	1089	1022	372	449	736	792	513	465	680	656	324	284	568	604	477	429	518	437
4	Complement C3 OS=Homo sapiens OX=9606 GN=C3 PE=1 SV=2	C3	187 kDa	118	118	107	91	33	41	43	76	133	137	74	60	602	512	243	251	325	326	145	182	91	78	167	167	259	251	173	164	364	332	395	357	265	200
5	Inter-alpha-trypsin inhibitor heavy chain H3 OS=Homo sapiens OX=9606 GN=ITIH3 PE=1 SV=2	ITIH3	100 kDa	5	5	20	16	1	1	5		25	22	5	19	500	362	812	805	634	621	333	414	506	598	399	412	403	444	168	160	238	240	411	385	316	233
6	Prothrombin OS=Homo sapiens OX=9606 GN=F2 PE=1 SV=2	F2	70 kDa	7	7	65	60	6	10	13	10	159	152	13	63	410	372	475	498	481	565	196	203	242	237	191	199	451	424	299	302	479	435	425	435	328	275
7	Complement C4-B OS=Homo sapiens OX=9606 GN=C4B PE=1 SV=2	C4B	193 kDa	47	47	165	142	39	57	26	37	168	169	40	40	277	245	343	360	376	392	170	226	337	315	269	253	238	234	152	129	338	357	339	305	270	221
8	Complement C4-A OS=Homo sapiens OX=9606 GN=C4A PE=1 SV=2	C4A	193 kDa	46	46	162	138	38	55	25	38	161	163		37	268	239	331	346	365	378	167	225	332	307	259	247	236	226	151	128	329	349	328	296	270	220
9	Serotransferrin OS=Homo sapiens OX=9606 GN=TF PE=1 SV=3	TF	77 kDa	110	110	139	127	70	76	68	98	114	102	58	65	262	244	146	158	178	205	123	134	77	70	117	117	173	166	88	75	236	218	147	130	125	104
10	Alpha-2-macroglobulin OS=Homo sapiens OX=9606 GN=A2M PE=1 SV=3	A2M	163 kDa	24	24	9	5	3	7	8		2	2	9	4	253	291	58	129	237	249	135	117	83	87	99	98	188	227	124	122	252	195	90	92	100	70
11	Immunoglobulin heavy constant gamma 2 OS=Homo sapiens OX=9606 GN=IGHG2 PE=1 SV=2	IGHG2	36 kDa	37	37	39	41	5	9	38	35	54	59	22	19	217	125	68	68	78	88	42	46	52	48	58	57	128	121	55	53	159	157	86	82	61	53
12	Immunoglobulin gamma-1 heavy chain OS=Homo sapiens OX=9606 PE=1 SV=2	IgG1	49 kDa	97	97	93	87	42	54	75	87	201	212	47	46	213	192	123	126	196	209	152	179	118	118	142	142	150	153	118	120	208	217	167	160	154	116
13	Hyaluronan-binding protein 2 OS=Homo sapiens OX=9606 GN=HABP2 PE=1 SV=1	HABP2	63 kDa			10	8		1	1		39	39	1	18	212	177	325	330	333	327	138	156	155	141	129	130	323	299	133	129	225	221	248	225	195	150
14	Fibrinogen beta chain OS=Homo sapiens OX=9606 GN=FGB PE=1 SV=2	FGB	56 kDa	19	19	11	9	14	18	4	5	3	5	4	8	195	161	189	187	201	226	150	172	188	182	115	120	199	191	64	66	154	156	156	153	98	76
15	Apolipoprotein B-100 OS=Homo sapiens OX=9606 GN=APOB PE=1 SV=2	APOB	516 kDa					7	15							194	302	36	191	327	336	48	32	24	23	30	32	151	264	57	67	206	134	85	112	134	92
16	Fibrinogen alpha chain OS=Homo sapiens OX=9606 GN=FGA PE=1 SV=2	FGA	95 kDa	13	13	10	10	5	10	2	2	8	4	5	5	174	181	191	203	209	231	145	168	168	151	131	129	191	183	68	58	81	72	142	127	102	82
17	Coagulation factor V OS=Homo sapiens OX=9606 GN=F5 PE=1 SV=4	F5	252 kDa	5	5	16	14	1	3		1	6	4	1	4	172	173	227	246	190	208	119	118	156	150	101	95	96	100	70	74	168	144	114	112	93	72
18	Fibrinogen gamma chain OS=Homo sapiens OX=9606 GN=FGG PE=1 SV=3	FGG	52 kDa	20	20	10	11	16	15	1	1	6	5	4	11	157	155	145	154	176	188	144	183	188	165	134	136	161	154	74	76	147	144	129	127	102	88
19	Immunoglobulin kappa constant OS=Homo sapiens OX=9606 GN=IGKC PE=1 SV=2	IGKC	12 kDa	55	55	69	63	30	34	46	49	124	133	30	35	157	151	101	111	156	158	107	131	101	89	102	102	146	133	94	88	157	152	115	119	108	102
20	Protein AMBP OS=Homo sapiens OX=9606 GN=AMBP PE=1 SV=1	AMBP	39 kDa	6	6	19	20	5	7	5	4	27	25	4	8	148	129	162	190	179	204	80	73	103	118	74	90	129	128	52	50	107	99	114	120	92	71
21	Haptoglobin OS=Homo sapiens OX=9606 GN=HP PE=1 SV=1	HP	45 kDa	18	18	20	22	3	7	10	2	6	6	7	13	138	123	71	70	75	76	49	54	30	31	39	36	57	55	47	43	114	99	12	12	55	52
22	Immunoglobulin heavy constant gamma 3 OS=Homo sapiens OX=9606 GN=IGHG3 PE=1 SV=2	IGHG3	41 kDa	42	42	43	43	15			42	91	94			131	108	76	76	117	121	63	67	62	62	70	68	105	104	57	58	134	127	88	90	72	60
23	Alpha-1-antitrypsin OS=Homo sapiens OX=9606 GN=SERPINA1 PE=1 SV=3	SERPINA1	47 kDa	58	58	81	69	31	40	31	45	78	82	25	30	126	108	135	119	90	100	62	62	48	53	67	57	85	83	53	57	65	66	82	85	57	54
24	Apolipoprotein A-I OS=Homo sapiens OX=9606 GN=APOA1 PE=1 SV=1	APOA1	31 kDa	116	116	150	133	57	59	65	70	173	152	62	69	125	139	109	128	136	143	93	115	95	101	87	75	128	121	123	130	152	134	118	126	104	112
25	Immunoglobulin kappa light chain OS=Homo sapiens OX=9606 PE=1 SV=1	IgK	23 kDa	37	37	44	43	20	23	27	30	85	91	18	22	119	112	66	75	118	120	75	101	71	63	74	73	110	101	65	61	117	113	82	82	76	73
26	Thrombospondin-1 OS=Homo sapiens OX=9606 GN=THBS1 PE=1 SV=2	THBS1	129 kDa							9				3	17	114	124	123	217	235	251	392	362	433	492	139	151	405	461	265	274	301	261	393	342	276	213
27	Cartilage oligomeric matrix protein OS=Homo sapiens OX=9606 GN=COMP PE=1 SV=2	COMP	83 kDa											1	4	114	109	120	135	131	136	64	69	103	96	114	120	122	123	82	77	113	106	136	132	94	84
28	Immunoglobulin heavy constant gamma 4 OS=Homo sapiens OX=9606 GN=IGHG4 PE=1 SV=1	IGHG4	36 kDa	37	37	50	44		17	22	33	73	85	14	21	103	99	69	72	80	92	65	66	61	62	64	72	82	89	55	60	99	103	62	61	68	52
29	Talin-1 OS=Homo sapiens OX=9606 GN=TLN1 PE=1 SV=3	TLN1	270 kDa									1	3			102	109	133	172	108	126	217	214	88	104	73	59	366	378	163	160	47	42	382	388	313	290
30	Coagulation factor IX OS=Homo sapiens OX=9606 GN=F9 PE=1 SV=2	F9	52 kDa			7	4			1		18	15	1	8	92	77	103	93	125	125	42	50	64	61	54	48	83	81	57	53	109	108	60	58	77	64
31	Hemopexin OS=Homo sapiens OX=9606 GN=HPX PE=1 SV=2	HPX	52 kDa	19	19	25	20	3	7	9	14	23	25	4	8	87	52	32	30	50	49	23	27	14	11	25	25	41	34	16	14	75	73	37	29	27	17
32	Filamin-A OS=Homo sapiens OX=9606 GN=FLNA PE=1 SV=4	FLNA	281 kDa							1						86	135	53	213	127	132	108	69	40	67	18	16	361	476	95	108	63	55	344	364	263	207
33	Ceruloplasmin OS=Homo sapiens OX=9606 GN=CP PE=1 SV=1	CP	122 kDa	130	130	172	154	64	83	20	84	203	195	43	26	85	76	38	45	75	88	28	30	19	19	27	28	55	48	33	27	58	49	57	54	32	26
34	Complement factor H OS=Homo sapiens OX=9606 GN=CFH PE=1 SV=4	CFH	139 kDa	6	6	7	2	1	1			2	5			83	78	7	32	63	67	19	13	7	7	8	8	44	48	6	6	54	30	45	41	19	18
35	Band 3 anion transport protein OS=Homo sapiens OX=9606 GN=SLC4A1 PE=1 SV=3	SLC4A1	102 kDa	5	5			55	69						3	79	79	110	126	73	75	94	105	76	73	75	76	177	177	86	81	88	76	112	107	55	54
36	Immunoglobulin heavy constant mu OS=Homo sapiens OX=9606 GN=IGHM PE=1 SV=4	IGHM	49 kDa	2	2			0	1	3	2	19	11	2	8	78	60	60	45	56	54	63	66	91	75	18	16	78	75	130	111	71	71	132	113	91	85
37	Vitamin D-binding protein OS=Homo sapiens OX=9606 GN=GC PE=1 SV=2	GC	53 kDa	27	27	49	45	10	18	7	11	70	71	6	5	77	62	30	25	49	65	34	25	14	16	26	21	46	44	39	38	50	49	47	51	67	42
38	Haptoglobin-related protein OS=Homo sapiens OX=9606 GN=HPR PE=2 SV=2	HPR	39 kDa	6	6	11	9					1	4		6	77	66	37	39	54	57	23	31	21	17	18	16	31	29	25	23	59	56	18	15	29	27
39	Coagulation factor X OS=Homo sapiens OX=9606 GN=F10 PE=1 SV=2	F10	55 kDa			5	4					7	8		7	73	49	120	97	85	98	26	32	40	38	21	24	82	74	46	39	84	79	74	62	70	40
40	Immunoglobulin heavy constant alpha 1 OS=Homo sapiens OX=9606 GN=IGHA1 PE=1 SV=2	IGHA1	38 kDa	17	17	11	10	7	6	10	10	42	38	12	11	67	61	55	63	65	76	85	80	53	49	57	49	56	54	37	34	41	38	50	52	67	59
41	Lumican OS=Homo sapiens OX=9606 GN=LUM PE=1 SV=2	LUM	38 kDa	15	15	26	22	13	13	7	11	23	27	2	8	66	57	73	67	68	67	60	53	76	89	82	77	62	66	57	53	50	53	62	55	53	54
42	Fibronectin OS=Homo sapiens OX=9606 GN=FN1 PE=1 SV=5	FN1	272 kDa	2	2	8	5	5	10			57	64			66	94	2	70	103	107	22	3	16	39	15	7	74	92	13	28	68	34	55	55	50	25
43	Hemoglobin subunit beta OS=Homo sapiens OX=9606 GN=HBB PE=1 SV=2	HBB	16 kDa	49	49	23	21	360	380	6	5	18	20	13	18	64	61	104	125	92	97	45	45	56	50	45	30	118	120	94	88	57	50	80	88	43	44
44	Actin, cytoplasmic 1 OS=Homo sapiens OX=9606 GN=ACTB PE=1 SV=1	ACTB	42 kDa	24	24	7	7	15	19	14	14	48	45	14	15	64	64	82	77	61	59	110	118	63	53	43	45	154	151	72	66	39	43	122	133	115	101
45	Vitronectin OS=Homo sapiens OX=9606 GN=VTN PE=1 SV=1	VTN	54 kDa	20	20	40	36	8	13	12	13	63	56	16	23	63	62	66	71	60	60	42	42	38	36	32	35	109	96	94	94	64	54	171	166	133	112
46	Keratin, type II cuticular Hb3 OS=Homo sapiens OX=9606 GN=KRT83 PE=1 SV=2	KRT83	54 kDa			60										58																				59	
47	Inter-alpha-trypsin inhibitor heavy chain H4 OS=Homo sapiens OX=9606 GN=ITIH4 PE=1 SV=4	ITIH4	103 kDa	19	19	39	32	5	7		4	44	34	3	1	55	41	35	33	58	59	21	32	20	20	20	23	55	60	18	12	38	37	30	30	17	12
48	Apolipoprotein E OS=Homo sapiens OX=9606 GN=APOE PE=1 SV=1	APOE	36 kDa	40	40	49	48	29	34	29	36	52	48	35	32	54	49	62	57	63	66	37	35	30	31	34	42	52	52	37	44	58	48	44	53	41	45
49	Endoplasmin OS=Homo sapiens OX=9606 GN=HSP90B1 PE=1 SV=1	HSP90B1	92 kDa	1	1		1					18	19			54	47	73	74	44	42	27	32	37	37	34	33	60	53	34	30	34	28	35	34	43	38
50	Keratin, type II cuticular Hb6 OS=Homo sapiens OX=9606 GN=KRT86 PE=1 SV=1	KRT86	53 kDa	2	2	66	19			3		11	1		9	54													1							56	
51	Transthyretin OS=Homo sapiens OX=9606 GN=TTR PE=1 SV=1	TTR	16 kDa	57	57	60	51	41	42	42	44	32	30	41	57	52	46	26	26	42	38	17	28	13	13	19	14	38	39	37	32	43	44	70	77	64	57
52	SPARC-like protein 1 OS=Homo sapiens OX=9606 GN=SPARCL1 PE=1 SV=2	SPARCL1	75 kDa	5	5	25	20	7	12		1	16	18	1		52	45	37	40	39	50	15	20	27	31	21	17	29	30	28	27	31	34	44	38	13	17
53	Immunoglobulin lambda constant 2 OS=Homo sapiens OX=9606 GN=IGLC2 PE=1 SV=1	IGLC2	11 kDa	25	25	26	19	5	7	23	29	65	58	12	14	52	51	41	47	48	58	42	49	39	31	42	34	45	47	40	40	55	63	44	47	39	34
54	Apolipoprotein A-IV OS=Homo sapiens OX=9606 GN=APOA4 PE=1 SV=3	APOA4	45 kDa	49	49	67	52	19	28	16	20	98	99	11	10	51	58	41	36	48	52	46	49	32	40	35	33	38	36	46	47	65	64	45	52	36	37
55	Antithrombin-III OS=Homo sapiens OX=9606 GN=SERPINC1 PE=1 SV=1	SERPINC1	53 kDa	15	15	29	29	3	4	2	3	23	31	0	2	51	40	23	19	36	31	15	20	8	6	17	16	37	34	21	18	31	36	35	31	40	30
56	Vitamin K-dependent protein Z OS=Homo sapiens OX=9606 GN=PROZ PE=1 SV=2	PROZ	45 kDa			1						5	4		2	50	42	72	57	47	50	22	26	60	67	34	32	35	34	37	38	46	44	32	38	22	18
57	Hemoglobin subunit alpha OS=Homo sapiens OX=9606 GN=HBA1 PE=1 SV=2	HBA1	15 kDa	34	34	18	16	234	254	2	3	7	11	4	7	49	45	78	105	58	76	22	25	24	27	22	18	91	90	52	44	32	24	50	60	31	24
58	Plasminogen OS=Homo sapiens OX=9606 GN=PLG PE=1 SV=2	PLG	91 kDa	5	5	10	10	0	2		2	8	7			49	51	15	17	45	39	10	16	7	5	8	8	36	31	5	5	47	41	22	22	18	8
59	Galectin-3-binding protein OS=Homo sapiens OX=9606 GN=LGALS3BP PE=1 SV=1	LGALS3BP	65 kDa	12	12	21	17	5	8	5	12	31	29	8	13	48	43	53	50	54	58	34	37	38	33	40	39	50	50	48	51	46	40	56	52	49	41
60	Complement C1s subcomponent OS=Homo sapiens OX=9606 GN=C1S PE=1 SV=1	C1S	77 kDa	8	8	19	17	4	6	3	12	41	51	5	6	48	33	53	48	43	54	30	32	26	22	16	22	59	54	27	29	57	47	41	42	27	23
61	Clusterin OS=Homo sapiens OX=9606 GN=CLU PE=1 SV=1	CLU	52 kDa	87	87	133	111	47	60	64	67	121	107	69	70	46	44	57	40	52	58	67	66	58	53	30	35	74	76	44	45	62	65	58	52	40	33
62	von Willebrand factor OS=Homo sapiens OX=9606 GN=VWF PE=1 SV=4	VWF	309 kDa													45	58	37	93	100	110	47	30	35	47	37	38	79	98	16	19	99	65	5	1	6	1
63	Kininogen-1 OS=Homo sapiens OX=9606 GN=KNG1 PE=1 SV=2	KNG1	72 kDa	23	23	47	42	13	14	7	13	45	51	5	9	45	36	25	28	33	37	25	27	30	25	21	21	47	47	25	21	35	36	42	45	43	32
64	Angiotensinogen OS=Homo sapiens OX=9606 GN=AGT PE=1 SV=1	AGT	53 kDa	22	22	19	13	6	10	8	11	16	17	6	7	45	36	33	37	37	40	19	24	36	35	22	19	28	28	26	24	33	33	38	33	23	18
65	Keratin, type I cuticular Ha1 OS=Homo sapiens OX=9606 GN=KRT31 PE=1 SV=3	KRT31	47 kDa	4	4	74	25			5		20			14	45													1				1			43	
66	Keratin, type II cuticular Hb5 OS=Homo sapiens OX=9606 GN=KRT85 PE=1 SV=1	KRT85	56 kDa	1	1	59	15			1		8			6	44	0				0		0	0									0			53	
67	Gelsolin OS=Homo sapiens OX=9606 GN=GSN PE=1 SV=1	GSN	86 kDa	31	31	58	45	28	31	16	32	38	34	21	20	43	38	29	32	36	37	37	47	27	25	16	19	50	50	25	22	35	36	49	42	39	28
68	Plasma protease C1 inhibitor OS=Homo sapiens OX=9606 GN=SERPING1 PE=1 SV=2	SERPING1	55 kDa	22	22	34	26	11	13	17	31	76	86	23	20	43	34	27	23	32	36	28	22	24	29	29	27	36	36	29	26	34	27	33	32	25	22
69	Vitamin K-dependent protein S OS=Homo sapiens OX=9606 GN=PROS1 PE=1 SV=1	PROS1	75 kDa	1	1	3	3	3	4		0	3	2	1	1	43	29	38	39	34	34	17	17	23	21	17	16	35	35	22	19	41	46	38	30	31	28
70	Thrombospondin-4 OS=Homo sapiens OX=9606 GN=THBS4 PE=1 SV=2	THBS4	106 kDa													42	40	41	54	56	65	28	29	54	48	42	41	42	41	27	20	39	29	65	58	37	30
71	Putative keratin-87 protein OS=Homo sapiens OX=9606 GN=KRT87P PE=5 SV=4	KRT87P	29 kDa			51										41																				63	
72	Ankyrin-1 OS=Homo sapiens OX=9606 GN=ANK1 PE=1 SV=3	ANK1	206 kDa	1	1			37	45							40	41	71	94	34	33	72	94	20	22	39	33	166	151	49	41	22	16	50	55	22	14
73	Immunoglobulin alpha-2 heavy chain OS=Homo sapiens OX=9606 PE=1 SV=2	IgA2	49 kDa	3	3	7	5	1	1	4	3	12	12	3	3	38	32	17	20	22	24	39	29	29	30	31	15	20	18	10	10	33	30	18	18	27	27
74	Complement factor B OS=Homo sapiens OX=9606 GN=CFB PE=1 SV=2	CFB	86 kDa	6	6	12	15	3	4	10	28	24	25	8	12	38	40	24	27	26	30	8	11	5	8	14	17	24	22	4	4	24	23	27	22	12	9
75	C4b-binding protein alpha chain OS=Homo sapiens OX=9606 GN=C4BPA PE=1 SV=2	C4BPA	67 kDa					1	2	2		1	1			36	54	29	54	67	75	6	10	18	18	15	11	52	63	34	33	53	49	65	75	74	48
76	Immunoglobulin lambda-1 light chain OS=Homo sapiens OX=9606 PE=1 SV=1	IgL1	23 kDa	9	9	15	8	3	3	12	8	38	40	6	6	36	44	15	26	35	42	26	31	16	13	17	13	26	29	26	33	45	52	23	23	16	13
77	Moesin OS=Homo sapiens OX=9606 GN=MSN PE=1 SV=3	MSN	68 kDa									1	1			35	25	32	24	24	26	17	23	18	21	8	12	88	82	30	33	42	34	68	61	53	39
78	Alpha-1-antichymotrypsin OS=Homo sapiens OX=9606 GN=SERPINA3 PE=1 SV=2	SERPINA3	48 kDa	22	22	37	32	10	14	22	28	40	62	15	14	35	23	24	20	25	29	12	19	17	17	15	14	20	21	15	16	21	20	17	15	12	15
79	Integrin alpha-IIb OS=Homo sapiens OX=9606 GN=ITGA2B PE=1 SV=3	ITGA2B	113 kDa												2	34	29	66	62	31	32	86	107	44	38	33	32	133	144	88	90	32	31	159	140	135	112
80	Hemoglobin subunit delta OS=Homo sapiens OX=9606 GN=HBD PE=1 SV=2	HBD	16 kDa	24	24	13	10	186	194						9	34	35	55	66	44	50	21	23	26	24	27	16	65	65	54	51	29	22	41	48	25	29
81	Complement C1r subcomponent OS=Homo sapiens OX=9606 GN=C1R PE=1 SV=2	C1R	80 kDa			8	6		1	2	6	37	52			33	32	47	52	51	57	63	77	31	24	13	11	79	81	22	22	47	47	24	20	20	16
82	Beta-2-glycoprotein 1 OS=Homo sapiens OX=9606 GN=APOH PE=1 SV=3	APOH	38 kDa	4	4	11	8	1	2	1	3	18	14		0	33	38	21	21	23	32	12	16	10	8	10	13	12	13	5	8	14	18	12	16	10	9
83	Complement C5 OS=Homo sapiens OX=9606 GN=C5 PE=1 SV=4	C5	188 kDa									5	9			33	31	10	16	18	25	7	8	4	0	7	7	17	14	9	7	25	23	16	13	3	3
84	Alpha-1B-glycoprotein OS=Homo sapiens OX=9606 GN=A1BG PE=1 SV=4	A1BG	54 kDa	8	8	11	10	5	4	3	3	16	15	1	0	32	31	18	16	29	25	15	21	10	9	17	15	23	15	7	7	24	23	18	16	14	9
85	Alpha-1-acid glycoprotein 1 OS=Homo sapiens OX=9606 GN=ORM1 PE=1 SV=1	ORM1	24 kDa	6	6	8	2	1	3	2	5	15	14	2	2	32	20	21	16	21	23	14	11	5	3	17	16	15	14	9	9	20	23	14	10	10	7
86	Platelet glycoprotein Ib alpha chain OS=Homo sapiens OX=9606 GN=GP1BA PE=1 SV=2	GP1BA	72 kDa									0	0			31	25	20	33	28	30	24	25	22	34	29	29	39	47	29	32	26	18	53	46	33	32
87	Serum paraoxonase/ arylesterase 1 OS=Homo sapiens OX=9606 GN=PON1 PE=1 SV=3	PON1	40 kDa	2	2	19	17	4	2			16	20		3	30	27	50	47	48	48	43	53	37	34	35	26	70	74	33	35	46	43	56	56	49	40
88	Keratin, type I cuticular Ha3-II OS=Homo sapiens OX=9606 GN=KRT33B PE=1 SV=3	KRT33B	46 kDa			55	20					15			14	30																				30	
89	Nidogen-1 OS=Homo sapiens OX=9606 GN=NID1 PE=1 SV=3	NID1	136 kDa			0						3	2			29	40	52	75	44	48	63	71	76	73	38	38	44	40	15	14	23	25	38	38	7	9
90	Complement component C9 OS=Homo sapiens OX=9606 GN=C9 PE=1 SV=2	C9	63 kDa	7	7	19	18	3	6	4	2	19	18	1	2	27	21	21	22	20	21	10	17	15	8	13	14	15	21	13	16	19	18	14	14	10	7
91	Alpha-2-antiplasmin OS=Homo sapiens OX=9606 GN=SERPINF2 PE=1 SV=3	SERPINF2	55 kDa	12	12	22	17	3	4	5	6	21	20	3	5	26	27	32	34	31	39	16	18	12	8	12	11	55	54	41	37	25	19	51	45	49	42
92	Heparin cofactor 2 OS=Homo sapiens OX=9606 GN=SERPIND1 PE=1 SV=3	SERPIND1	57 kDa	4	4	22	18	1	3	1	0	14	16	0	1	26	18	14	16	21	23	15	15	9	9	6	7	27	27	12	10	20	16	6	7	10	8
93	Keratin, type I cuticular Ha4 OS=Homo sapiens OX=9606 GN=KRT34 PE=1 SV=2	KRT34	49 kDa			39	17					13			10	25		1	0					1	0	1					1	1		1		19	1
94	Alpha-2-HS-glycoprotein OS=Homo sapiens OX=9606 GN=AHSG PE=1 SV=2	AHSG	39 kDa	13	13	31	30	13	15	6	9	26	20	2	3	23	18	21	19	22	23	5	8	8	9	9	12	26	23	4	3	15	11	18	15	5	4
95	Apolipoprotein A-II OS=Homo sapiens OX=9606 GN=APOA2 PE=1 SV=1	APOA2	11 kDa	15	15	29	24	6	10	15	12	16	14	13	18	23	25	20	23	22	25	11	15	10	12	8	5	21	24	14	14	26	19	18	22	10	7
96	Vinculin OS=Homo sapiens OX=9606 GN=VCL PE=1 SV=4	VCL	124 kDa													22	20	32	34	21	22	48	50	30	34	17	14	93	88	41	43	14	12	111	103	65	62
97	Calreticulin OS=Homo sapiens OX=9606 GN=CALR PE=1 SV=1	CALR	48 kDa	2	2	11	6	1	3	3	3	24	20	2	1	22	23	27	26	19	23	20	23	24	28	16	17	23	20	29	33	24	31	30	28	33	28
98	Afamin OS=Homo sapiens OX=9606 GN=AFM PE=1 SV=1	AFM	69 kDa	4	4	4	3		1	1	1	14	13		1	22	19	7	8	12	20	6	3	2	2	4	5	13	10	3	1	21	18	5	5	8	5
99	Fermitin family homolog 3 OS=Homo sapiens OX=9606 GN=FERMT3 PE=1 SV=1	FERMT3	76 kDa									1	1			21	24	42	46	23	32	52	63	29	19	24	20	100	99	47	43	12	9	92	95	86	72
100	Integrin beta-3 OS=Homo sapiens OX=9606 GN=ITGB3 PE=1 SV=2	ITGB3	87 kDa												3	21	19	35	26	17	20	45	56	20	15	14	15	76	79	42	40	18	15	76	74	58	54
101	Neural cell adhesion molecule 1 OS=Homo sapiens OX=9606 GN=NCAM1 PE=1 SV=3	NCAM1	95 kDa	6	6	5	7	3	5		1	8	9	0		21	20	18	24	16	26	13	19	20	22	21	18	23	26	18	17	25	14	32	28	14	7
102	Protein Z-dependent protease inhibitor OS=Homo sapiens OX=9606 GN=SERPINA10 PE=1 SV=1	SERPINA10	51 kDa			1						4	3			21	21	34	37	25	32	4	3	14	13	11	8	17	16	18	19	19	17	16	16	11	7
103	Erythrocyte membrane protein band 4.2 OS=Homo sapiens OX=9606 GN=EPB42 PE=1 SV=3	EPB42	77 kDa					14	15							18	21	36	41	14	15	26	30	14	13	20	22	80	72	21	17	19	18	37	35	7	8
104	Alpha-actinin-1 OS=Homo sapiens OX=9606 GN=ACTN1 PE=1 SV=2	ACTN1	103 kDa	4	4				1			2	2			18	15	5	10	3	10	69	78	28	24	4	5	26	17	4	3	14	10	44	38	13	12
105	Vitamin K-dependent protein C OS=Homo sapiens OX=9606 GN=PROC PE=1 SV=1	PROC	52 kDa			1	2					7	9			17	17	16	15	20	23	9	11	11	12	5	6	20	20	13	11	26	29	14	12	14	15
106	Apolipoprotein D OS=Homo sapiens OX=9606 GN=APOD PE=1 SV=1	APOD	21 kDa	9	9	21	23	18	21	4	9	31	36	7	11	16	18	9	9	19	22	8	11	17	18	14	10	20	17	18	19	26	27	8	10	2	4
107	14-3-3 protein zeta/ delta OS=Homo sapiens OX=9606 GN=YWHAZ PE=1 SV=1	YWHAZ	28 kDa	9	9	2		6	11			16	17	2		16	16	21	25	14	19	26	28	19	18	15	13	41	41	32	28	14	15	36	42	40	36
108	Alpha-1-acid glycoprotein 2 OS=Homo sapiens OX=9606 GN=ORM2 PE=1 SV=2	ORM2	24 kDa	4	4	4						9	8			16	10	12	8	7	12	8	6	4	4	7	10	8	10	7	7	12	10	10	8	7	6
109	Ras-related protein Rap-1b OS=Homo sapiens OX=9606 GN=RAP1B PE=1 SV=1	RAP1B	21 kDa										1			15	15	20	33	15	21	29	36	15	8	7	11	79	88	28	22	15	13	56	54	52	38
110	Histidine-rich glycoprotein OS=Homo sapiens OX=9606 GN=HRG PE=1 SV=1	HRG	60 kDa	12	12	14	10	6	9	2	4	14	14	1	3	15	14	10	9	17	20	15	17	14	18	10	12	21	21	19	17	10	9	49	58	42	36
111	Peroxiredoxin-2 OS=Homo sapiens OX=9606 GN=PRDX2 PE=1 SV=5	PRDX2	22 kDa	10	10	1	1	32	35	1	1	2	2	1	2	15	17	18	21	12	11	11	11	11	9	12	12	23	19	15	12	7	6	20	21	9	8
112	Lipopolysaccharide-binding protein OS=Homo sapiens OX=9606 GN=LBP PE=1 SV=3	LBP	53 kDa			1	1					8	9			15	9	34	27	9	8	6	6	7	5	7	6	19	18	7	7	18	15	4	5	4	5
113	Zinc-alpha-2-glycoprotein OS=Homo sapiens OX=9606 GN=AZGP1 PE=1 SV=2	AZGP1	34 kDa	7	7	12	4	4	1	3	3	11	9	4	5	15	16	6	3	11	13	3	2	1	2	4	4	8	9	3	3	17	16	3	3	2	3
114	Coagulation factor XIII A chain OS=Homo sapiens OX=9606 GN=F13A1 PE=1 SV=4	F13A1	83 kDa													14	12	13	20	6	7	17	20	12	11	4	4	37	33	12	10	4	4	32	31	27	17
115	Plasma serine protease inhibitor OS=Homo sapiens OX=9606 GN=SERPINA5 PE=1 SV=3	SERPINA5	46 kDa			1	1	2				0	2		2	14	11	17	16	19	17	11	14	8	5	5	7	5	3	22	23	23	20	26	25	4	7
116	Complement factor H-related protein 1 OS=Homo sapiens OX=9606 GN=CFHR1 PE=1 SV=2	CFHR1	38 kDa										1			14	12	3	5	6	6	1	3	2		1		5	4			7	6	3		1	1
117	Heat shock protein HSP 90-alpha OS=Homo sapiens OX=9606 GN=HSP90AA1 PE=1 SV=5	HSP90AA1	85 kDa	10	10	2	1	5	6			23	20	1	4	13	11	29	27	31	36	16	21	13	9	14	10	69	56	67	57	31	25	82	79	68	52
118	Serum amyloid P-component OS=Homo sapiens OX=9606 GN=APCS PE=1 SV=2	APCS	25 kDa									3	4			13	14	7	5	9	8	4	6	5	4	7	7	7	7	4	2	11	10	6	6	7	6
119	Multimerin-1 OS=Homo sapiens OX=9606 GN=MMRN1 PE=1 SV=3	MMRN1	138 kDa													12	11	18	25	36	33	91	89	73	76	18	13	90	92	49	54	60	49	81	84	56	47
120	Tubulin alpha-1B chain OS=Homo sapiens OX=9606 GN=TUBA1B PE=1 SV=1	TUBA1B	50 kDa	10	10		1	1	3			25	23			12	15	19	24	11	14	34	31	28	21	6	3	34	34	31	26	9	9	40	40	37	31
121	Cofilin-1 OS=Homo sapiens OX=9606 GN=CFL1 PE=1 SV=3	CFL1	19 kDa	2	2			2	3			5	5			12	10	12	11	13	11	10	12	3	2	3	3	29	31	10	6	3	3	27	25	21	20
122	Catalase OS=Homo sapiens OX=9606 GN=CAT PE=1 SV=3	CAT	60 kDa	3	3			20	27	1	2					12	5	20	15	6	7	8	9	8	9	8	6	22	19	9	11	2	1	25	22	3	2
123	Retinol-binding protein 4 OS=Homo sapiens OX=9606 GN=RBP4 PE=1 SV=3	RBP4	23 kDa	10	10	10	11	2	4			10	12		0	12	21	3	2	18	23	4	2	3	1	2	3	9	7	1	1	13	12	3	4	3	3
124	Ficolin-3 OS=Homo sapiens OX=9606 GN=FCN3 PE=1 SV=2	FCN3	33 kDa													12	9	1	1	1		1	2	4	3			3	2	0		3	1	1			
125	Keratin, type II cytoskeletal 1 OS=Homo sapiens OX=9606 GN=KRT1 PE=1 SV=6	KRT1	66 kDa	256	256	192	118	246	187	205	215	143	157	223	211	11	32	12	23	29	46	32	20	23	23	14	11	45	50	108	112	31	39	35	51	140	51
126	Transitional endoplasmic reticulum ATPase OS=Homo sapiens OX=9606 GN=VCP PE=1 SV=4	VCP	89 kDa	0	0			7	7			7	9			11	11	31	32	22	27	14	17	13	13	9	11	56	58	39	34	30	22	49	54	27	30
127	Erythrocyte band 7 integral membrane protein OS=Homo sapiens OX=9606 GN=STOM PE=1 SV=3	STOM	32 kDa					7	8							11	12	35	43	23	30	26	36	20	17	11	10	45	43	19	17	14	11	28	27	27	24
128	Ezrin OS=Homo sapiens OX=9606 GN=EZR PE=1 SV=4	EZR	69 kDa													11	10	8		5	9	5	9	8	9	4		27	26			14	10	18	16	15	9
129	Protein disulfide-isomerase OS=Homo sapiens OX=9606 GN=P4HB PE=1 SV=3	P4HB	57 kDa									11	8			11	7	13	9	11	13	5	6	7	5	2	1	9	10	10	10	15	10	8	13	6	6
130	Complement component C8 beta chain OS=Homo sapiens OX=9606 GN=C8B PE=1 SV=3	C8B	67 kDa	1	1	2	2				1	3	4			11	4	2	4	7	8	4	8	3	1	1	2	10	7	2	2	10	7	11	11	9	5
131	Kallistatin OS=Homo sapiens OX=9606 GN=SERPINA4 PE=1 SV=3	SERPINA4	49 kDa	3	3	2	1				0	4	5			11	5	2	2	5	4	3	1	2	1	3	2	3	3	1	1	6	11	2	2	2	4
132	Keratin, type I cuticular Ha5 OS=Homo sapiens OX=9606 GN=KRT35 PE=2 SV=5	KRT35	50 kDa			21										11																				22	
133	Complement component C6 OS=Homo sapiens OX=9606 GN=C6 PE=1 SV=3	C6	105 kDa			4						2	3			10	11	1	1	5	10	0	2			2	2	2	2	2		7	5	1	3	1	0
134	Tubulin alpha-4A chain OS=Homo sapiens OX=9606 GN=TUBA4A PE=1 SV=1	TUBA4A	50 kDa									17	15			10	14	14	21	9	12	31	27	26	18	5		32	33	27	22	8	7	37	38	35	26
135	Heat shock protein HSP 90-beta OS=Homo sapiens OX=9606 GN=HSP90AB1 PE=1 SV=4	HSP90AB1	83 kDa	10	10							21	18			10	11	16	18	20	22	9	13	9			8	44	36	33	27	21	17	41	40	30	29
136	Pigment epithelium-derived factor OS=Homo sapiens OX=9606 GN=SERPINF1 PE=1 SV=4	SERPINF1	46 kDa	30	30	35	33	17	23	24	29	18	19	27	26	9	8	2	3	6	7		2	3	2	4	2	3	1			2	3		2	1	1
137	Keratin, type II cytoskeletal 75 OS=Homo sapiens OX=9606 GN=KRT75 PE=1 SV=2	KRT75	60 kDa									13				9																				14	
138	Myosin-9 OS=Homo sapiens OX=9606 GN=MYH9 PE=1 SV=4	MYH9	227 kDa	11	11				1			24	28			9	6		6	2	4	23	18	29	22	1		18	36	2	2	3	1	20	22	11	8
139	Profilin-1 OS=Homo sapiens OX=9606 GN=PFN1 PE=1 SV=2	PFN1	15 kDa	1	1							3	3		1	9	10	14	16	10	12	16	17	14	13	5	4	32	26	16	14	10	8	26	26	22	18
140	Tubulin beta-1 chain OS=Homo sapiens OX=9606 GN=TUBB1 PE=1 SV=1	TUBB1	50 kDa	1	1							1	0			9	11	13	13	3	5	18	21	14	10	1	1	32	26	8	4	3	2	22	19	28	21
141	Complement component C7 OS=Homo sapiens OX=9606 GN=C7 PE=1 SV=2	C7	94 kDa	2	2	2	1					1	3			9	10			5	6	1	4	1		3	2	1	4	1	2	11	12	1	2	1	1
142	Tubulin alpha-1A chain OS=Homo sapiens OX=9606 GN=TUBA1A PE=1 SV=1	TUBA1A	50 kDa									23	21			9	11	14	20		11	28	28					29	28	25				33	37	32	26
143	Neuropilin-1 OS=Homo sapiens OX=9606 GN=NRP1 PE=1 SV=3	NRP1	103 kDa									1				8	5	6	9	8	8	4	5	10	9	6	2	8	6	6	5	7	6	10	6	4	4
144	N-acetylmuramoyl-L-alanine amidase OS=Homo sapiens OX=9606 GN=PGLYRP2 PE=1 SV=1	PGLYRP2	62 kDa			4	2					5	6			8	6	5	5	3	10	4	5	1	2	3	1	11	10	1	2	2	2	4	4	1	1
145	Apolipoprotein C-I OS=Homo sapiens OX=9606 GN=APOC1 PE=1 SV=1	APOC1	9 kDa			2	1					3	1		0	8	9	5	9	8	9	2	3	2	1	1	1	4	7	3	4	6	3	3	6	2	4
146	Keratin, type II cuticular Hb2 OS=Homo sapiens OX=9606 GN=KRT82 PE=3 SV=3	KRT82	57 kDa	0	0	10										8																				20	
147	Complement factor H-related protein 2 OS=Homo sapiens OX=9606 GN=CFHR2 PE=1 SV=1	CFHR2	31 kDa													8	11																				
148	Glyceraldehyde-3-phosphate dehydrogenase OS=Homo sapiens OX=9606 GN=GAPDH PE=1 SV=3	GAPDH	36 kDa	46	46	10	8	12	13	7	5	33	30	12	10	7	5	11	11	6	8	7	10	6	2	3	2	28	23	23	23	3	1	29	43	35	31
149	Latent-transforming growth factor beta-binding protein 1 OS=Homo sapiens OX=9606 GN=LTBP1 PE=1 SV=4	LTBP1	187 kDa													7	8	6	16	17	17	31	24	23	32	6	6	37	42	12	13	15	14	28	26	11	6
150	WD repeat-containing protein 1 OS=Homo sapiens OX=9606 GN=WDR1 PE=1 SV=4	WDR1	66 kDa	1	1											7	8	12	10	6	11	16	20	7	4	2	6	46	45	5	6	4	3	37	44	33	28
151	14-3-3 protein epsilon OS=Homo sapiens OX=9606 GN=YWHAE PE=1 SV=1	YWHAE	29 kDa	7	7			4	7			8	7			7	8	10	11	7	9	12	11	5	3	2		22	21	21	21	12	9	23	24	17	16
152	Beta-parvin OS=Homo sapiens OX=9606 GN=PARVB PE=1 SV=1	PARVB	42 kDa													7	4	12	11	5	6	13	16	6	7	6	3	30	28	10	15	4	3	28	37	26	22
153	Pleckstrin OS=Homo sapiens OX=9606 GN=PLEK PE=1 SV=3	PLEK	40 kDa													7	7	7	9	3	7	9	12	6	3	1	2	41	41	10	9	3	2	31	29	34	17
154	Apolipoprotein C-III OS=Homo sapiens OX=9606 GN=APOC3 PE=1 SV=1	APOC3	11 kDa	3	3	1	1			1			1	2	2	7	13	11	13	13	14	7	7	7	7	3	3	10	10	8	5	13	10	8	10	10	11
155	Heat shock cognate 71 kDa protein OS=Homo sapiens OX=9606 GN=HSPA8 E=1 SV=1	HSPA8	71 kDa	4	4			5	8			2	2	1		7	5	6	9	5	7	15	19	8	8	4	5	16	16	7	8	3	5	16	14	11	8
156	CD5 antigen-like OS=Homo sapiens OX=9606 GN=CD5L PE=1 SV=1	CD5L	38 kDa													7	8	3	6	2	4	3	5	6	5		2	9	10	11	11	4	5	16	15	7	6
157	Immunoglobulin kappa variable 3-20 OS=Homo sapiens OX=9606 GN=IGKV3-20 PE=1 SV=2	IGKV3-20	13 kDa	2	2	2	1	1	1	2	2	4	3	2	2	7	6	3	2	8	7	5	6	4	3	3	5	6	6	4	5	4	5	6	7	4	4
158	Corticosteroid-binding globulin OS=Homo sapiens OX=9606 GN=SERPINA6 PE=1 SV=1	SERPINA6	45 kDa			2	2				1	2	3			7	5	3	0	4	5	1	2			4	2	3	3	1	1	5	5	3	2	2	2
159	Leucine-rich alpha-2-glycoprotein OS=Homo sapiens OX=9606 GN=LRG1 PE=1 SV=2	LRG1	38 kDa			0						1	1			7	5	5	6	3	2	1				1	1	1	1					1	4	0	
160	Keratin, type II cytoskeletal 6A OS=Homo sapiens OX=9606 GN=KRT6A PE=1 SV=3	KRT6A	60 kDa	47	47	50	25	67	48	75	59	43	47	84	65	7										5	4		10	24	35		8		20	41	9
161	Immunoglobulin heavy variable 3-7 OS=Homo sapiens OX=9606 GN=IGHV3-7 PE=1 SV=2	IGHV3-7	13 kDa	1	1	1	1			3	3	5	3		2	7	6	5	3	5	5	4	4	5	3	3	3	6	6	7	5	6	5	5	5	5	5
162	Keratin, type I cytoskeletal 17 OS=Homo sapiens OX=9606 GN=KRT17 PE=1 SV=2	KRT17	48 kDa	18	18	17	7	22	20	26	22	9	10	46	21	7														8	7						3
163	Keratin, type I cuticular Ha6 OS=Homo sapiens OX=9606 GN=KRT36 PE=2 SV=1	KRT36	52 kDa			16										7																				15	
164	Keratin, type II cytoskeletal 6B OS=Homo sapiens OX=9606 GN=KRT6B PE=1 SV=5	KRT6B	60 kDa	44	44	47		60		70	58			83	60	7	7				11							7	9			6	9	6			
165	Fibulin-1 OS=Homo sapiens OX=9606 GN=FBLN1 PE=1 SV=4	FBLN1	77 kDa	6	6	13	10	3	4	4	5	3	1	4	3	6	5	2	2	4	3	1	4	4	1	3		20	20	3	2	1	2	19	21	14	13
166	Phosphatidylinositolglycan-specific phospholipase D OS=Homo sapiens OX=9606 GN=GPLD1 PE=1 SV=3	GPLD1	92 kDa									4	4			6	8	4	3	11	5	7	9	1	1	0	1	15	13	2	1	11	9	4	4	6	5
167	CD44 antigen OS=Homo sapiens OX=9606 GN=CD44 PE=1 SV=3	CD44	82 kDa									3	2			6	4	6	5	6	7	4	5	8	9	6	7	4	4	4	4	4	3	7	7	6	4
168	Zyxin OS=Homo sapiens OX=9606 GN=ZYX PE=1 SV=1	ZYX	61 kDa													6	5	7	5	6	6	4	6	1				14	13	1				14	11	4	2
169	Plasma kallikrein OS=Homo sapiens OX=9606 GN=KLKB1 PE=1 SV=1	KLKB1	71 kDa													6	3	1	0	6	4	1	4	2	1	0	2	11	13		0	5	3	6	3	5	4
170	Extracellular matrix protein 2 OS=Homo sapiens OX=9606 GN=ECM2 PE=2 SV=1	ECM2	80 kDa													6	5	4	6	7	8	1	3	4	6	2	1	1	1	1	1	7	6		1		
171	Carboxypeptidase N subunit 2 OS=Homo sapiens OX=9606 GN=CPN2 PE=1 SV=3	CPN2	61 kDa			1						1	1			6	2	4	1	3	6	2	2	2	3	0		3	4		1	4	6	5	3	4	2
172	Insulin-like growth factor-binding protein complex acid labile subunit OS=Homo sapiens OX=9606 GN=IGFALS PE=1 SV=1	IGFALS	66 kDa			2	1					2	4			6	6	3	2	3	5	4	2	1	1	3	1	8	6	1	2	1	2			3	2
173	Annexin A6 OS=Homo sapiens OX=9606 GN=ANXA6 PE=1 SV=3	ANXA6	76 kDa													6	3			2	2							21	15	1	1			1	1		
174	Dermatopontin OS=Homo sapiens OX=9606 GN=DPT PE=1 SV=2	DPT	24 kDa	1	1							1	2			6	3	5	7	8	8	5	7	5	6	3	4	5	6	1	1	3	2	6	5	5	5
175	Keratin, type I cytoskeletal 14 OS=Homo sapiens OX=9606 GN=KRT14 PE=1 SV=4	KRT14	52 kDa	52	52	42	23	71	44	57	48	30	31	62	56	5	2		1		1	3			4	3	3	5	4	19	20		3	3	11	36	9
176	Carbonic anhydrase 1 OS=Homo sapiens OX=9606 GN=CA1 PE=1 SV=2	CA1	29 kDa					105	118							5	5	5	8	9	12	1	1	1		1		16	11	3	3	3	1	3	4	1	1
177	Integrin-linked protein kinase OS=Homo sapiens OX=9606 GN=ILK PE=1 SV=2	ILK	51 kDa													5	2	8	13	6	9	10	7	1	0	1	1	29	30	4	4	0		27	36	17	14
178	HLA class I histocompatibility antigen, A alpha chain OS=Homo sapiens OX=9606 GN=HLA-A PE=1 SV=2	HLA-A	41 kDa								1	9	9		1	5	6	10	9	2	5	6	6	6	3	4	4	20	21	8	6	6	4	11	13	10	12
179	Apolipoprotein L1 OS=Homo sapiens OX=9606 GN=APOL1 PE=1 SV=5	APOL1	44 kDa			1						1	3			5	7	9	9	9	8	9	11	9	8	3	3	4	5	8	5	5	4	7	9	12	10
180	L-lactate dehydrogenase B chain OS=Homo sapiens OX=9606 GN=LDHB PE=1 SV=2	LDHB	37 kDa	7	7			3	6			1	1			5	4	6	6	2	3	8	8	4	2	3	1	19	20	3	3	2	1	10	13	7	6
181	Collagen alpha-1(XVIII) chain OS=Homo sapiens OX=9606 GN=COL18A1 PE=1 SV=5	COL18A1	178 kDa	3	3	4	6	2	2		1	2	1			5	6	5	9	9	5	2	7	5	4	3	4	6	6	6	5	9	6	6	6	4	2
182	Protein disulfide-isomerase A3 OS=Homo sapiens OX=9606 GN=PDIA3 PE=1 SV=4	PDIA3	57 kDa													5	3	5	5	4	6	7	10	9	11	2	2	6	7	6	4	5	4	6	7	3	3
183	Platelet glycoprotein IX OS=Homo sapiens OX=9606 GN=GP9 PE=1 SV=3	GP9	19 kDa													5	6	7	8	3	7	4	5	2	2	1	1	12	11	5	4	3	3	12	10	11	7
184	Immunoglobulin kappa variable 4-1 OS=Homo sapiens OX=9606 GN=IGKV4-1 PE=1 SV=1	IGKV4-1	13 kDa	1	1	2	2				1	5	5		1	5	3	4	4	2	4	4	3	3	3	3	3	4	4	5	5	4	4	4	4	3	3
185	Osteomodulin OS=Homo sapiens OX=9606 GN=OMD PE=1 SV=1	OMD	49 kDa			2	3		1		2		1			5	4	4	5	3	2	3	2	8	8	4	5	2	3	4	4		2	2	1	1	
186	Glucosidase 2 subunit beta OS=Homo sapiens OX=9606 GN=PRKCSH PE=1 SV=2	PRKCSH	59 kDa			1	1					3	3			5	5	8	7	4	5	2	7	5	2	3	4	3	2	6	3	2	2	3	2	4	3
187	Apolipoprotein C-II OS=Homo sapiens OX=9606 GN=APOC2 PE=1 SV=1	APOC2	11 kDa													5	5	7	8	7	9	2	2	1				5	5		2	6	6	3	6	2	3
188	Cholesteryl ester transfer protein OS=Homo sapiens OX=9606 GN=CETP PE=1 SV=2	CETP	55 kDa									1	2			5	5	3	3	3	3			3	2	1	1	7	6	3	4	4	2	6	3		2
189	Solute carrier family 2, facilitated glucose transporter member 1 OS=Homo sapiens OX=9606 GN=SLC2A1 PE=1 SV=2	SLC2A1	54 kDa					4	2							5	4	9	10	4	5	5	3	3	0	1	3	15	13	5	5	5	5	8	10	3	2
190	Immunoglobulin heavy variable 3-74 OS=Homo sapiens OX=9606 GN=IGHV3-74 PE=3 SV=1	IGHV3-74	13 kDa	1	1		1			3	3	3	3	2	2	5	4	3	3	6	5	3	3	3	3	2	1	6	4	5	5	6	6	5	5	5	4
191	Ribosome-binding protein 1 OS=Homo sapiens OX=9606 GN=RRBP1 PE=1 SV=5	RRBP1	152 kDa	2	2	3	3	4	4	2	2	3	4	2		5	5	4	4	5	4	6	3	3	4	5	6	4	3	3	5	6	8	3	3	4	4
192	14-3-3 protein beta/ alpha OS=Homo sapiens OX=9606 GN=YWHAB PE=1 SV=3	YWHAB	28 kDa	2	2			1				7	6			5	6	7	7	6	7	10	11	3	4			15	17	18	14	8	6	19	23	20	17
193	14-3-3 protein theta OS=Homo sapiens OX=9606 GN=YWHAQ PE=1 SV=1	YWHAQ	28 kDa	2	2			1	3			5	4			5		8	4	4	4	7	9				4	17	18	12	8	7	5	16	15	15	12
194	Immunoglobulin heavy variable 3-43D OS=Homo sapiens OX=9606 GN=IGHV3-43D PE=3 SV=1	IGHV3-43D	13 kDa													5	4		1	4	5		2					4	4	4	3	4	4		3		
195	Tubulin beta chain OS=Homo sapiens OX=9606 GN=TUBB PE=1 SV=2	TUBB	50 kDa	6	6			0	1			15	17			4	8	11	15	4	13	18	18	13	8		1	29	26	21	19	3	6	42	39	31	20
196	Tropomyosin alpha-4 chain OS=Homo sapiens OX=9606 GN=TPM4 PE=1 SV=3	TPM4	29 kDa	1	1		0	1	1			3	2	1	0	4	9	9	10	5	7	19	22	8	11	1	4	14	20	7	8	6	4	11	14	11	10
197	Peptidyl-prolyl cis-trans isomerase A OS=Homo sapiens OX=9606 GN=PPIA PE=1 SV=2	PPIA	18 kDa	1	1				1							4	5	8	12	5	6	6	8	8	5	3	2	24	21	8	7		1	13	15	12	10
198	Mannan-binding lectin serine protease 1 OS=Homo sapiens OX=9606 GN=MASP1 PE=1 SV=3	MASP1	79 kDa													4	5	7	6	6	7	8	8	7	9	5	2	8	10	6	5	9	7	10	7	7	5
199	Secreted phosphoprotein 24 OS=Homo sapiens OX=9606 GN=SPP2 PE=1 SV=1	SPP2	24 kDa													4	5	2	3	7	6	6	5	3	3	3	1	10	11	6	5	6	5	7	12	9	5
200	Platelet factor 4 OS=Homo sapiens OX=9606 GN=PF4 PE=1 SV=2	PF4	11 kDa													4	4	5	4	6	4	4	7	7	5	1		9	7	6	4	9	7	8	8	4	3
201	Protein disulfide-isomerase A4 OS=Homo sapiens OX=9606 GN=PDIA4 PE=1 SV=2	PDIA4	73 kDa				1					3	3			4	1	7	10	3	1	2	3	4	4	2	4	4	3	5	6	2	4	7	5	7	6
202	Actin-related protein 3 OS=Homo sapiens OX=9606 GN=ACTR3 PE=1 SV=3	ACTR3	47 kDa													4	4	4	3	2	1	3	5	1		0		15	15	2	1	1	2	11	16	10	10
203	Complement factor I OS=Homo sapiens OX=9606 GN=CFI PE=1 SV=2	CFI	66 kDa	1	1	7	2		1	0	1	2	3	0	0	4	3	2	1	4	2	2	2	1	1	2	2	3	2	1	1	4	5	3	2	1	1
204	Immunoglobulin heavy variable 5-51 OS=Homo sapiens OX=9606 GN=IGHV5-51 PE=3 SV=1	IGHV5-51	13 kDa	1	1	1						2	2			4	5	0	2	1	4	1		2			0	3	2	2	2	3	2	4	3	1	1
205	Coagulation factor XII OS=Homo sapiens OX=9606 GN=F12 PE=1 SV=3	F12	68 kDa				1					3	3			4	4	1	1	2	4	0	1	1		0		5	3	1		4	3	2	1	1	1
206	Pyruvate kinase PKM OS=Homo sapiens OX=9606 GN=PKM PE=1 SV=4	PKM	58 kDa	50	50	4	1	6	10			17	19	0	1	4	6	9	9	8	7	14	22	5	3	1	2	33	30	14	11			24	27	38	30
207	Bone marrow proteoglycan OS=Homo sapiens OX=9606 GN=PRG2 PE=1 SV=2	PRG2	25 kDa													4	2	9	11	4	3	3	4	6	7	5	5	6	4	2	4	7	4	6	5	2	4
208	C4b-binding protein beta chain OS=Homo sapiens OX=9606 GN=C4BPB PE=1 SV=1	C4BPB	28 kDa													4	2	3	3	4	6	1	1	2	3	2	1	4	4	1	2	2	5	6	2	3	3
209	L-selectin OS=Homo sapiens OX=9606 GN=SELL PE=1 SV=2	SELL	42 kDa									1	1			4	1	4	2	1	5	2	1	4	4	2	4	7	9	3	5	3	3	6	5	2	3
210	Ras-related protein Rab-10 OS=Homo sapiens OX=9606 GN=RAB10 PE=1 SV=1	RAB10	23 kDa													4	3	6	9		3	6	9	4			3	8	13	4	6	5	3	9	10	8	8
211	Immunoglobulin heavy variable 4-61 OS=Homo sapiens OX=9606 GN=IGHV4-61 PE=3 SV=1	IGHV4-61	13 kDa	1	1	1	1		1	1	1	5	6	1	1	4	3	3	3	5	6	2	2	1	1	1	2	4	5	1	2	4	3	4	5	3	2
212	Immunoglobulin lambda variable 3-21 OS=Homo sapiens OX=9606 GN=IGLV3-21 PE=1 SV=2	IGLV3-21	12 kDa	1	1	1	1			2	1	4	3	1	1	4	3	2	0	4	3	2	3	1	1	1	1	3	3	3	1	4	4	2	2	2	2
213	Keratin, type II cytoskeletal 2 epidermal OS=Homo sapiens OX=9606 GN=KRT2 PE=1 SV=2	KRT2	65 kDa	129	129	110	77	178	120	127	146	87	97	143	144	4	16	5	14	9	21	17	5	12	13	9	8	22	20	74	57	15	26	22	18	89	28
214	Endoplasmic reticulum chaperone BiP OS=Homo sapiens OX=9606 GN=HSPA5 PE=1 SV=2	HSPA5	72 kDa	2	2							3				4	4	3	5	3	6	10	15	9	9	2	3	7	7	3	4	7	7	3	7	4	3
215	Keratin, type I cytoskeletal 16 OS=Homo sapiens OX=9606 GN=KRT16 PE=1 SV=4	KRT16	51 kDa	38	38	34	21	47	33	58	40	33	29	52	49	4															17		2		9	22	
216	Keratin, type I cytoskeletal 13 OS=Homo sapiens OX=9606 GN=KRT13 PE=1 SV=4	KRT13	50 kDa			20							11	29		4																					
217	14-3-3 protein eta OS=Homo sapiens OX=9606 GN=YWHAH PE=1 SV=4	YWHAH	28 kDa	1	1							5	3			4	5	9	6	5	6	8	11	5	3	3	4	18	14	10	8	7	6	16	18	14	12
218	Keratin, type I cytoskeletal 10 OS=Homo sapiens OX=9606 GN=KRT10 PE=1 SV=6	KRT10	59 kDa	221	221	138	118	229	213	194	246	83	105	230	226	3	15	5	16	20	28	19	7	10	16	7	7	33	35	116	74	20	36	25	30	128	36
219	Platelet glycoprotein V OS=Homo sapiens OX=9606 GN=GP5 PE=1 SV=1	GP5	61 kDa													3	2	5	6	1	3	6	8	6	4	4	1	34	34	9	6			19	19	21	15
220	Guanine nucleotide-binding protein G(q) subunit alpha OS=Homo sapiens OX=9606 GN=GNAQ PE=1 SV=4	GNAQ	42 kDa													3	0	2	3	0	0	4	6	1		0		8	8	3	5	1	0	7	9	12	11
221	Plexin domain-containing protein 1 OS=Homo sapiens OX=9606 GN=PLXDC1 PE=1 SV=2	PLXDC1	56 kDa													3	3	6	5	2	3	1	0	2	2	2	0	1	2		0	1	2	3	3		
222	Sex hormone-binding globulin OS=Homo sapiens OX=9606 GN=SHBG PE=1 SV=2	SHBG	44 kDa													3	0		1	1		5	5			1		2	3	4	3		0	4	5	2	1
223	Adipocyte plasma membrane-associated protein OS=Homo sapiens OX=9606 GN=APMAP PE=1 SV=2	APMAP	46 kDa													3	2	1		0			1					2	1	1				2	2	3	3
224	Platelet endothelial cell adhesion molecule OS=Homo sapiens OX=9606 GN=PECAM1 PE=1 SV=2	PECAM1	83 kDa													3	3	7	9	3	5	7	10	2	3	2	1	30	24	9	7		1	30	23	12	15
225	Calnexin OS=Homo sapiens OX=9606 GN=CANX PE=1 SV=2	CANX	68 kDa	1	1							1	1			3	4	9	5	1	1	7	7	6	6	1	1	6	5	1	1	3	4	2	2	0	4
226	Leukocyte surface antigen CD47 OS=Homo sapiens OX=9606 GN=CD47 PE=1 SV=1	CD47	35 kDa						0							3		2	2	1	1	3	2	1	2		2	3	3	3	3	2	1	3	4	2	2
227	Tenascin OS=Homo sapiens OX=9606 GN=TNC PE=1 SV=3	TNC	241 kDa									10	18			3	21	1	9	9	14							10	27			4	1	0	3		
228	Protein 4.1 OS=Homo sapiens OX=9606 GN=EPB41 PE=1 SV=4	EPB41	97 kDa					2	2							3	3	3	11	2	3	2	6				1	14	13	0		2	1	4	3		0
229	Immunoglobulin J chain OS=Homo sapiens OX=9606 GN=JCHAIN PE=1 SV=4	JCHAIN	18 kDa				1			1	1	1	1	1	1	3	3	4	3	3	2	2	3	3	2	3	2	3	2	2	4	3	2	5	5	1	2
230	Beta-Ala-His dipeptidase OS=Homo sapiens OX=9606 GN=CNDP1 PE=1 SV=4	CNDP1	57 kDa									1				3	1		1		1	4	5					1	3	1	1					3	2
231	Collagen alpha-1(XII) chain OS=Homo sapiens OX=9606 GN=COL12A1 PE=1 SV=2	COL12A1	333 kDa													3	2	0	1	2				1	1	0		1	2	2	2	2		8	7	0	1
232	Coagulation factor XIII B chain OS=Homo sapiens OX=9606 GN=F13B PE=1 SV=3	F13B	76 kDa													3	5	1	1	5	6	1	3	2		0		1	1			1	1			1	
233	Integrin beta-1 OS=Homo sapiens OX=9606 GN=ITGB1 PE=1 SV=2	ITGB1	88 kDa													3	0	2	5			6	6	0	0		0	14	17	5	6	2	2	19	14	12	5
234	Immunoglobulin kappa variable 3-11 OS=Homo sapiens OX=9606 GN=IGKV3-11 PE=1 SV=1	IGKV3-11	13 kDa	0	0	1	1	0	1	0	1	4	5		0	3	2		1	3	3	1	2	1	1	2	3	2	2	3	2	2	3	3	3	2	3
235	Complement component C8 gamma chain OS=Homo sapiens OX=9606 GN=C8G PE=1 SV=3	C8G	22 kDa	1	1	1	2					1	1			3	3	2	2	4	4	1	1					2	2	1		3	2	1	1		1
236	Coronin-1A OS=Homo sapiens OX=9606 GN=CORO1A PE=1 SV=4	CORO1A	51 kDa									1	1			3	4	0	3	2	3	4	5	1		1		9	8	2	1			4	6	5	5
237	HLA class I histocompatibility antigen, C alpha chain OS=Homo sapiens OX=9606 GN=HLA-C PE=1 SV=3	HLA-C	41 kDa									20	16			3	4	5	6	3	5	5	7	3	4	3	3	16	17	8	8	4	4	14	14	11	11
238	Ras-related protein Rab-1B OS=Homo sapiens OX=9606 GN=RAB1B PE=1 SV=1	RAB1B	22 kDa	1	1							1	0			3	3	5	7	3	2	6	9	3	2	2	2	9	11	2	5	2	2	10	8	5	8
239	Triosephosphate isomerase OS=Homo sapiens OX=9606 GN=TPI1 PE=1 SV=3	TPI1	31 kDa	5	5			4	4			1				3	3	6	6	4	4	6	6	1	1	1	1	11	10	2	4	1	1	8	10	4	6
240	Complement C1q subcomponent subunit C OS=Homo sapiens OX=9606 GN=C1QC PE=1 SV=3	C1QC	26 kDa			1				1	3	4	5	1	1	3	4	2	4	3	4	1	1			2	2	1	1	2	1	3	3	1	2	1	1
241	Probable non-functional immunoglobulin kappa variable 2D-24 OS=Homo sapiens OX=9606 GN=IGKV2D-24 PE=5 SV=1	IGKV2D-24	13 kDa									1	1			3	1	1		1	1	1		1	1		1	2	1	1	1	2	2	2	1	1	1
242	Adenylyl cyclase-associated protein 1 OS=Homo sapiens OX=9606 GN=CAP1 PE=1 SV=5	CAP1	52 kDa									2	2			3	4	7	6	4	2	16	19	12	10	5	3	23	15	6	6	1	1	15	18	11	10
243	Immunoglobulin lambda variable 1-51 OS=Homo sapiens OX=9606 GN=IGLV1-51 PE=1 SV=2	IGLV1-51	12 kDa									5	6			3	3	1	1	1	2	2	1	3	3	0	1	1	1	5	3	1	1	1	1	0	0
244	Thrombospondin-3 OS=Homo sapiens OX=9606 GN=THBS3 PE=1 SV=1	THBS3	104 kDa													3	4	3	5	6	8		6	6	7	4	5	8	5	6	4		4	4	5	4	1
245	Immunoglobulin kappa variable 3-15 OS=Homo sapiens OX=9606 GN=IGKV3-15 PE=1 SV=2	IGKV3-15	12 kDa			1	2				0	5	3			3	3	1	3	3	5	5	2	2	2	3	3	4	3	3	2	4	2	3	4	2	2
246	Platelet glycoprotein 4 OS=Homo sapiens OX=9606 GN=CD36 PE=1 SV=2	CD36	53 kDa													2	4	4	1	4	5	7	12	3	2	5	5	17	20	14	10	1	2	18	15	18	17
247	Keratin, type I cytoskeletal 9 OS=Homo sapiens OX=9606 GN=KRT9 PE=1 SV=3	KRT9	62 kDa	220	220	154	83	241	170	171	200	85	86	205	156	2	21	4	3	15	24	16	9	16	10	5	7	23	28	68	81	15	21	13	19	75	28
248	Complement C1q subcomponent subunit B OS=Homo sapiens OX=9606 GN=C1QB PE=1 SV=3	C1QB	27 kDa							3	3	4	4	1	2	2	4	1		2	2	1	2	1	1	1	1	2	1	1	1	3	3	1	1	1	1
249	CD9 antigen OS=Homo sapiens OX=9606 GN=CD9 PE=1 SV=4	CD9	25 kDa													2	4	2	7	2	5	4	5	1	1	0	2	12	13	10	8	1	2	8	10	16	9
250	Apolipoprotein M OS=Homo sapiens OX=9606 GN=APOM PE=1 SV=2	APOM	21 kDa										0			2	1	1	1	2	4		1			0	0	1	1	0	0	2	2	1	1	0	0
251	Immunoglobulin heavy variable 3-72 OS=Homo sapiens OX=9606 GN=IGHV3-72 PE=3 SV=1	IGHV3-72	13 kDa									0	0			2	1	0	0		0					0	0	1		0		1	0		1	0	0
252	SPARC OS=Homo sapiens OX=9606 GN=SPARC PE=1 SV=1	SPARC	35 kDa	2	2	2	2		1			1	1			2	1	6	5	18	16	11	14	8	17	3	4	17	21	7	8	20	27	21	16	6	6
253	Integrin alpha-6 OS=Homo sapiens OX=9606 GN=ITGA6 PE=1 SV=5	ITGA6	127 kDa													2	1	6	6	3	1	4	15	3		2	1	27	25	9	8	2	2	29	29	24	19
254	Complement C2 OS=Homo sapiens OX=9606 GN=C2 PE=1 SV=2	C2	83 kDa									5	5	0		2	2			2	2					1	1					0	2				
255	Complement component C8 alpha chain OS=Homo sapiens OX=9606 GN=C8A PE=1 SV=2	C8A	65 kDa			2	0					1	1			2	4		1	2	4		3					3	2	1		2	4	3	2	1	1
256	EGF-containing fibulin-like extracellular matrix protein 1 OS=Homo sapiens OX=9606 GN=EFEMP1 PE=1 SV=2	EFEMP1	55 kDa	21	21	28	30	15	18	20	31	17	16	10	17	2	2				1	0		1			0	2	1	1		1	1				0
257	Fructose-bisphosphate aldolase A OS=Homo sapiens OX=9606 GN=ALDOA PE=1 SV=2	ALDOA	39 kDa	10	10			1	5			1				2	4	6	6	1	4	15	21	7	3	2	3	21	16	8	3	3	3	15	19	16	15
258	HLA class I histocompatibility antigen, B alpha chain OS=Homo sapiens OX=9606 GN=HLA-B PE=1 SV=3	HLA-B	40 kDa									9	7			2	3	4	4	3	4	5	6	4	3	3	3	15	17	11	11	5	3	11	15	12	14
259	Phospholipid transfer protein OS=Homo sapiens OX=9606 GN=PLTP PE=1 SV=1	PLTP	55 kDa	5	5	10	7	1	2	2	8	14	18	1	1	2	1	3	1	2	1	2	1	2	2	2	2	3	3	2		3	3	1	1	3	
260	C-type lectin domain family 11 member A OS=Homo sapiens OX=9606 GN=CLEC11A PE=1 SV=1	CLEC11A	36 kDa									2	2			2	3	8	8	3	6	4	2	9	4	5	3	2	3	3	2	2	3	8	7	2	
261	ADP-ribosylation factor 3 OS=Homo sapiens OX=9606 GN=ARF3 PE=1 SV=2	ARF3	21 kDa						0			2	1			2	2	1	3	2	4	2	6	2	2		0	10	9	4	2	3	3	6	5	7	4
262	Actin-related protein 2/3 complex subunit 1B OS=Homo sapiens OX=9606 GN=ARPC1B PE=1 SV=3	ARPC1B	41 kDa													2	2	3	4	2	2	5	3	0		1		9	10	2	2			10	12	6	6
263	Serum amyloid A-4 protein OS=Homo sapiens OX=9606 GN=SAA4 PE=1 SV=2	SAA4	15 kDa			3	1			1		3	2	2	1	2	3	3	5	2	5		1	2	0	1		2	2	0		5	5	1	3	1	1
264	Proteoglycan 4 OS=Homo sapiens OX=9606 GN=PRG4 PE=1 SV=3	PRG4	151 kDa													2	3	4	3	2	1		0	0				4	2		1	3	2		1	2	1
265	Immunoglobulin heavy variable 3-49 OS=Homo sapiens OX=9606 GN=IGHV3-49 PE=3 SV=1	IGHV3-49	13 kDa									0	1			2	1		1	1	2	1	1		1	2	1	2	2	1	0	2	1	1	1	1	1
266	Histone H2B type 1-K OS=Homo sapiens OX=9606 GN=H2BC12 PE=1 SV=3	H2BC12	14 kDa	5	5	1						3	3			2																			1	1	
267	Immunoglobulin kappa variable 1-17 OS=Homo sapiens OX=9606 GN=IGKV1-17 PE=1 SV=2	IGKV1-17	13 kDa													2	3			2	2	1	1					1				1	1	2	1	1	
268	Complement C1q subcomponent subunit A OS=Homo sapiens OX=9606 GN=C1QA PE=1 SV=2	C1QA	26 kDa									1	0			2	1			1	1		0					1	1			1	1				
269	Plastin-2 OS=Homo sapiens OX=9606 GN=LCP1 PE=1 SV=6	LCP1	70 kDa									5	4			2	2			0	2							9	8	1	1		1	6	4	1	1
270	Adiponectin OS=Homo sapiens OX=9606 GN=ADIPOQ PE=1 SV=1	ADIPOQ	26 kDa													2	1			1	1							1	1			1	1	1	1		
271	Platelet glycoprotein Ib beta chain OS=Homo sapiens OX=9606 GN=GP1BB PE=1 SV=1	GP1BB	22 kDa													2	2	5	5	2	5	4	4	2	2			10	8	4	2	1	2	7	10	8	7
272	Junctional adhesion molecule A OS=Homo sapiens OX=9606 GN=F11R PE=1 SV=1	F11R	33 kDa													2	2	3	4	1	3	3	4	1	1	2	2	10	9	4	2	2	2	6	6	6	9
273	Mannan-binding lectin serine protease 2 OS=Homo sapiens OX=9606 GN=MASP2 PE=1 SV=4	MASP2	76 kDa													2	1	3	4	5	4	5	5					5	3	1	1	6	2			2	1
274	Neuropilin-2 OS=Homo sapiens OX=9606 GN=NRP2 PE=1 SV=3	NRP2	105 kDa									2	2			2	3	6	7	4	2	1	4	7	4	3	1	2	3	1	1	4	3	3	4	2	1
275	Chromogranin-A OS=Homo sapiens OX=9606 GN=CHGA PE=1 SV=7	CHGA	51 kDa	1	1	5	4	1	1			5	5	1	1	2	3	2	3	3	6			2	1					1			2	1			
276	Extracellular matrix protein 1 OS=Homo sapiens OX=9606 GN=ECM1 PE=1 SV=2	ECM1	61 kDa	2	2	3	3	2	3	1	1	3	3			2	1	1	2	3	5	1	1	1	1	2	1		2	1	1			2	2	2	0
277	Proto-oncogene tyrosine-protein kinase Src OS=Homo sapiens OX=9606 GN=SRC PE=1 SV=3	SRC	60 kDa													2	3	4	6	1	1	4	7	1	1	1	1	21	20	5	3			21	16	14	9
278	Apolipoprotein F OS=Homo sapiens OX=9606 GN=APOF PE=1 SV=2	APOF	35 kDa			2	1					3	1			2	3	6	5	4	5	2	2	4	3	1	1	3	2	2	2	3	4	3	2	2	1
279	Ras suppressor protein 1 OS=Homo sapiens OX=9606 GN=RSU1 PE=1 SV=3	RSU1	32 kDa													2	3	3	7	4	4	11	18	7	4	4	2	24	27	13	13	1	1	21	28	18	14
280	Histone H4 OS=Homo sapiens OX=9606 GN=H4C1 PE=1 SV=2	H4C1	11 kDa	2	2	1				1		3	4	1	0	2					0															2	
281	Immunoglobulin lambda variable 8-61 OS=Homo sapiens OX=9606 GN=IGLV8-61 PE=3 SV=7	IGLV8-61	13 kDa									1	1			2	4	1	1	1	3		2					2	2	1	2	2	1	2	2	2	1
282	Keratin, type II cytoskeletal 1b OS=Homo sapiens OX=9606 GN=KRT77 PE=2 SV=3	KRT77	62 kDa	28	28	17	11	25	16	26	24	15	15	28	19	2	5	3	3	4	6	4	3	4	3	3	3	5	6	14	14	4	6	4	4	15	6
283	Putative HLA class I histocompatibility antigen, alpha chain H OS=Homo sapiens OX=9606 GN=HLA-H PE=5 SV=3	HLA-H	41 kDa									8	8			2	3	4	4	2	3	3	3	2		2		9	8	5	4	3	3	8	8	7	
284	Serine/threonine-protein phosphatase 6 regulatory ankyrin repeat subunit B OS=Homo sapiens OX=9606 GN=ANKRD44 PE=1 SV=3	ANKRD44	108 kDa	1	1	1	1	1	2		0	2	2			2	2	1	2	1	3	0	2	2	0	2	2	6	7	6	5	1	1	9	6	6	4
285	Phosphatidylinositol 4-phosphate 3-kinase C2 domain-containing subunit alpha OS=Homo sapiens OX=9606 GN=PIK3C2A PE=1 SV=2	PIK3C2A	191 kDa													2	3	0	2	0	1		0		1	0		0	1	0		2	0	0			
286	Ras-related protein Rab-8A OS=Homo sapiens OX=9606 GN=RAB8A PE=1 SV=1	RAB8A	24 kDa													2	3	6	8	3	3	5	8	4				11	11	3	4			10	8	9	6
287	Amyloid-beta precursor protein OS=Homo sapiens OX=9606 GN=APP PE=1 SV=3	APP	87 kDa	2	2	24	19	8	13		1	2	1	3		1	2	6	6	7	9	17	17	19	20	4	3	17	19	15	14	16	14	34	28	12	9
288	Ubiquitin-40S ribosomal protein S27a OS=Homo sapiens OX=9606 GN=RPS27A PE=1 SV=2	RPS27A	18 kDa	4	4	2	3	6	7	3	2	3	2	2	3	1	3	2	3	3	2	2	2	2	2	1	1	5	6	2	2	1	1	5	4	3	1
289	LIM and senescent cell antigen-like-containing domain protein 1 OS=Homo sapiens OX=9606 GN=LIMS1 PE=1 SV=4	LIMS1	37 kDa													1	3	2	5	3	3	4	4	2	1	1	1	16	17	3	3	1	1	13	16	5	6
290	Immunoglobulin kappa variable 2D-29 OS=Homo sapiens OX=9606 GN=IGKV2D-29 PE=3 SV=1	IGKV2D-29	13 kDa	1	1		1	1	1	2	1	3	3	1	2	1	2	2	1	2	1	2	1	2	1	1		1	2	2	2	2	1	2	2	2	1
291	Spectrin beta chain, erythrocytic OS=Homo sapiens OX=9606 GN=SPTB PE=1 SV=5	SPTB	246 kDa					4	10							1	3	3	9	1	1	2	1	0	0	0	0	30	32	2	3		1	19	29		
292	Phosphoglycerate kinase 1 OS=Homo sapiens OX=9606 GN=PGK1 PE=1 SV=3	PGK1	45 kDa	10	10			1	4				1	1	1	1	1	1	2		0	3	5	2	1	1		5	7	1	2			4	2	4	2
293	Ras-related C3 botulinum toxin substrate 2 OS=Homo sapiens OX=9606 GN=RAC2 PE=1 SV=1	RAC2	21 kDa									2				1	1	1	2	3	2	1	2	1				9	10	4	5	2	1	5	8	5	8
294	Ras-related protein Rab-27B OS=Homo sapiens OX=9606 GN=RAB27B PE=1 SV=4	RAB27B	25 kDa													1		2	3	1	1	11	12	2	1			9	7	5	3	1		7	7	9	8
295	Arf-GAP with GTPase, ANK repeat and PH domain-containing protein 9 OS=Homo sapiens OX=9606 GN=AGAP9 PE=3 SV=2	AGAP9	78 kDa													1	1	2	1	1	2		0					2	1				1	2	1		
296	Collectin-11 OS=Homo sapiens OX=9606 GN=COLEC11 PE=1 SV=1	COLEC11	29 kDa													1	2	1	3	1	3	1	2	1				2	1	0	0	2	1	1	1	1	1
297	Guanine nucleotide-binding protein G(i) subunit alpha-2 OS=Homo sapiens OX=9606 GN=GNAI2 PE=1 SV=3	GNAI2	40 kDa										1			1	5	4	9	2	3	3	6	1		1	1	20	27	5	2	5	6	14	17	14	11
298	Glycophorin-A OS=Homo sapiens OX=9606 GN=GYPA PE=1 SV=2	GY PA	16 kDa					2	4							1	2	1	1	2	2	3	7	4	3	3	3	1	1	4	4	2	3	4	4		1
299	Acylamino-acid-releasing enzyme OS=Homo sapiens OX=9606 GN=APEH PE=1 SV=4	APEH	81 kDa	4	4			6	10							1	2	1	5			1		0				3	3	2	4			2	3		
300	Pentraxin-related protein PTX3 OS=Homo sapiens OX=9606 GN=PTX3 PE=1 SV=3	PTX3	42 kDa													1	2			1	2		0	1	1			1	1	6	2	2	1	1	1	2	1
301	Spectrin alpha chain, erythrocytic 1 OS=Homo sapiens OX=9606 GN=SPTA1 PE=1 SV=5	SPTA1	280 kDa					9	13							1	4	9	20	4	6	4	1					40	54	5	2	6	7	25	27	1	1
302	Receptor-type tyrosine-protein phosphatase zeta OS=Homo sapiens OX=9606 GN=PTPRZ1 PE=1 SV=4	PTPRZ1	255 kDa			3	1					2	2			1	2			3	1				0		1	0	4		1	1	1	3	4		
303	Alpha-enolase OS=Homo sapiens OX=9606 GN=ENO1 PE=1 SV=2	ENO1	47 kDa	100	100	3	1	9	13	3	6	6	5	17	11	1	3	3	2	6	5	12	14	1	1	2	1	22	18	11	12	1	2	14	13	13	10
304	Transgelin-2 OS=Homo sapiens OX=9606 GN=TAGLN2 PE=1 SV=3	TAGLN2	22 kDa													1	1	4	9	2	4	11	11	9	9			17	14	6	5	1	0	13	16	7	10
305	Monocyte differentiation antigen CD14 OS=Homo sapiens OX=9606 GN=CD14 PE=1 SV=2	CD14	40 kDa	2	2	10	4	1	2	4	8	27	29	2	2	1	3	3	5	2	2	5	5	3	1	2	2	2	4	2	1	0	2				
306	Transforming growth factor-beta-induced protein ig-h3 OS=Homo sapiens OX=9606 GN=TGFBI PE=1 SV=1	TGFBI	75 kDa	8	8	4	3	1	2	1	1	14	12	0		1	1	3	4	5	3	2	2	10	6	1	0	1	2	0	0	5	4	4	5	1	0
307	Cell division control protein 42 homolog OS=Homo sapiens OX=9606 GN=CDC42 PE=1 SV=2	CDC42	21 kDa													1	1	5	6		1	2	4					14	15	8	5	1	1	10	14	11	8
308	Carbonic anhydrase 2 OS=Homo sapiens OX=9606 GN=CA2 PE=1 SV=2	CA2	29 kDa					27	31							1	0	1	3	2	3	1	3	1				4	5	4	3	1	1	5	3	5	3
309	Coronin-1C OS=Homo sapiens OX=9606 GN=CORO1C PE=1 SV=1	CORO1C	53 kDa													1	3	3	5	4	3	8	9	0	3		1	14	9	2	2	1	1	11	7	8	9
310	Chloride intracellular channel protein 1 OS=Homo sapiens OX=9606 GN=CLIC1 PE=1 SV=4	CLIC1	27 kDa									2	2			1	3	3	4	1	1	3	6	4	2	1	2	12	7	2	2	2	3	7	7	2	3
311	Annexin A2 OS=Homo sapiens OX=9606 GN=ANXA2 PE=1 SV=2	ANXA2	39 kDa	8	8	3	2	10	8	5	3	3	2	6	3	1	1	1	1	1			1					2	2	2	1		1	1	1	2	
312	Guanine nucleotide-binding protein G(I)/G(S)/G(T) subunit beta-1 OS=Homo sapiens OX=9606 GN=GNB1 PE=1 SV=3	GNB1	37 kDa									2	2			1	1	3	3			2	4					12	8	2	2	1	1	5	7	9	6
313	Cartilage acidic protein 1 OS=Homo sapiens OX=9606 GN=CRTAC1 PE=1 SV=2	CRTAC1	71 kDa	3	3	6	7	2	1			3	4			1	2	1	1	1	2	1	3	4	2	3	1	1	0	0			0	3	1	0	
314	Actin-related protein 2/3 complex subunit 3 OS=Homo sapiens OX=9606 GN=ARPC3 PE=1 SV=3	ARPC3	21 kDa													1		2	3		2	2	2		1			7	5	1	1			6	7	5	3
315	Apolipoprotein(a) OS=Homo sapiens OX=9606 GN=LPA PE=1 SV=1	LPA	501 kDa													1	13		3	5	5							3	13					1	4		
316	ATP synthase subunit beta, mitochondrial OS=Homo sapiens OX=9606 GN=ATP5F1B PE=1 SV=3	ATP5F1B	57 kDa	19	19											1						3	6														
317	Vasodilator-stimulated phosphoprotein OS=Homo sapiens OX=9606 GN=VASP PE=1 SV=3	VASP	40 kDa													1	2	1	2	2	1	2	2		1			6	5			1	0	2	6	2	
318	Blood group Rh(D) polypeptide OS=Homo sapiens OX=9606 GN=RHD PE=1 SV=3	RHD	45 kDa													1	1	2	2	1	1	1	1	1	1	1	1	2	4	2	1	1	1	2	2	1	1
319	Drebrin-like protein OS=Homo sapiens OX=9606 GN=DBNL PE=1 SV=1	DBNL	48 kDa													1	1	1	2		2	2	4					4	6					5	5	5	2
320	Tetranectin OS=Homo sapiens OX=9606 GN=CLEC3B PE=1 SV=3	CLEC3B	23 kDa	2	2	6	5			1	1	4	4		1	1		1		2									0						1		
321	Solute carrier family 2, facilitated glucose transporter member 3 OS=Homo sapiens OX=9606 GN=SLC2A3 PE=1 SV=1	SLC2A3	54 kDa													1	1		1			3	2			1		1	1	1	1		1	2	1	4	2
322	Flotillin-2 OS=Homo sapiens OX=9606 GN=FLOT2 PE=1 SV=2	FLOT2	47 kDa													1		1	2	1	1	1	1					6	5			1	1	1	1		
323	Immunoglobulin lambda variable 3-19 OS=Homo sapiens OX=9606 GN=IGLV3-19 PE=1 SV=2	IGLV3-19	12 kDa									2	3			1	1		1	1	1	1	1	1	1	1	2	1	1	1	1	1	1	1	1	1	1
324	Immunoglobulin lambda variable 1-47 OS=Homo sapiens OX=9606 GN=IGLV1-47 PE=1 SV=2	IGLV1-47	12 kDa	1	1	1	1			2	2	2	2			1	2		1	1	1	1	1	1					1				1	1			
325	Immunoglobulin heavy variable 1-69 OS=Homo sapiens OX=9606 GN=IGHV1-69 PE=1 SV=2	IGHV1-69	13 kDa									2	3			1	1	1	1	1	2						1	1	1			1	2	2	3	1	1
326	26S proteasome non-ATPase regulatory subunit 2 OS=Homo sapiens OX=9606 GN=PSMD2 PE=1 SV=3	PSMD2	100 kDa													1		1										2	2	3	4			3	3	2	1
327	Rho GDP-dissociation inhibitor 1 OS=Homo sapiens OX=9606 GN=ARHGDIA PE=1 SV=3	ARHGDIA	23 kDa						1							1		1	1	1	1	1	1	1	1			2	1	1	1			1	1	1	1
328	Oncoprotein-induced transcript 3 protein OS=Homo sapiens OX=9606 GN=OIT3 PE=1 SV=2	OIT3	60 kDa													1		1	1	2	3				1				1			2	3				
329	Endosialin OS=Homo sapiens OX=9606 GN=CD248 PE=1 SV=1	CD248	81 kDa													1	2			1										0		1					
330	EMILIN-1 OS=Homo sapiens OX=9606 GN=EMILIN1 PE=1 SV=3	EMILIN1	107 kDa			3	1									1		1		2		14	17	19	17	2	2	13	16			3	2	2	2	1	1
331	EH domain-containing protein 1 OS=Homo sapiens OX=9606 GN=EHD1 PE=1 SV=2	EHD1	61 kDa													1	1	2	2	2	2	5	7		0			13	8	4	5			18	18	11	10
332	Biotinidase OS=Homo sapiens OX=9606 GN=BTD PE=1 SV=2	BTD	61 kDa	2	2	4	3	1	2	1	2	1	3	1	1	1							1	0		1	1			1		1	1				
333	Platelet glycoprotein VI OS=Homo sapiens OX=9606 GN=GP6 PE=1 SV=4	GP6	37 kDa													1		3						2			1	5	3	2	1		1	8	6	2	2
334	FYN-binding protein 1 OS=Homo sapiens OX=9606 GN=FYB1 PE=1 SV=2	FYB1	85 kDa													1	0	2					1					4	4	1				5	6	2	3
335	Transforming protein RhoA OS=Homo sapiens OX=9606 GN=RHOA PE=1 SV=1	RHOA	22 kDa													1	0	1	2	0	1	3	3	1				6	6	2	2			4	5	2	3
336	Versican core protein OS=Homo sapiens OX=9606 GN=VCAN PE=1 SV=3	VCAN	373 kDa			0							0			1	2	2	2	1	2		1	2	1	2	1	4	1	1	1	1	1	3	2	1	1
337	Polymeric immunoglobulin receptor OS=Homo sapiens OX=9606 GN=PIGR PE=1 SV=4	PIGR	83 kDa									4	2			1				1													1				
338	Tyrosine-protein kinase BTK OS=Homo sapiens OX=9606 GN=BTK PE=1 SV=3	BTK	76 kDa													1												2	1					0		0	1
339	Nucleosome assembly protein 1-like 1 OS=Homo sapiens OX=9606 GN=NAP1L1 PE=1 SV=1	NAP1L1	45 kDa									1	1			1	1	3	4	2	3	4	4	1	0	0		4	4	8	8	1	2	8	9	7	6
340	Methanethiol oxidase OS=Homo sapiens OX=9606 GN=SELENBP1 PE=1 SV=2	SELENBP1	52 kDa					7	10							1																					
341	Soluble scavenger receptor cysteine-rich domain-containing protein SSC5D OS=Homo sapiens OX=9606 GN=SSC5D PE=1 SV=3	SSC5D	166 kDa													0	13		10	15	19							2	15			5	4	4	16	2	1
342	Calponin-2 OS=Homo sapiens OX=9606 GN=CNN2 PE=1 SV=4	CNN2	34 kDa													0	0		2			1	2	2				4	3	0				2	1	2	3
343	Platelet basic protein OS=Homo sapiens OX=9606 GN=PPBP PE=1 SV=3	PPBP	14 kDa													0	3	4	4	1	2	1	2	3	4	1		2	2	1		4	1	1	3	1	
344	Protein S100-A8 OS=Homo sapiens OX=9606 GN=S100A8 PE=1 SV=1	S100A8	11 kDa	2	2		1	1		1	1	1	2	2	3	0	2	2	1	3	4	0						6	6	9	11	7	4	15	18	16	18
345	Integrin alpha-2 OS=Homo sapiens OX=9606 GN=ITGA2 PE=1 SV=1	ITGA2	129 kDa													0												3	11	2	5	0		11	8	9	7
346	Actin-related protein 2/3 complex subunit 5 OS=Homo sapiens OX=9606 GN=ARPC5 PE=1 SV=3	ARPC5	16 kDa													0						1						2	2					2	3	2	1
347	Importin subunit beta-1 OS=Homo sapiens OX=9606 GN=KPNB1 PE=1 SV=2	KPNB1	97 kDa									2	1			0	1	2	4		2	3	2	1	1	1	2	4	7	6	7	0		6	7	1	2
348	Sarcoplasmic/endoplasmic reticulum calcium ATPase 3 OS=Homo sapiens OX=9606 GN=ATP2A3 PE=1 SV=2	ATP2A3	114 kDa													0	0	0	0	0	0	3	3	1	0			0	0	0		0	0	0	0	0	0
349	Fibrocystin-L OS=Homo sapiens OX=9606 GN=PKHD1L1 PE=2 SV=2	PKHD1L1	466 kDa													0	0			1	0		0					0	3			0	0	0	0		
350	CD151 antigen OS=Homo sapiens OX=9606 GN=CD151 PE=1 SV=3	CD151	28 kDa													0							1					1	1					2	0	1	1
351	Histone H2A type 1-B/E OS=Homo sapiens OX=9606 GN=H2AC4 PE=1 SV=2	H2AC4	14 kDa	3	3	1						2	2			0																				2	
352	Heat shock protein beta-1 OS=Homo sapiens OX=9606 GN=HSPB1 PE=1 SV=2	HSPB1	23 kDa	1	1	1		7	7					1		0	1		0			2	3	2				1	1						2	1	0
353	Coagulation factor VIII OS=Homo sapiens OX=9606 GN=F8 PE=1 SV=1	F8	267 kDa													0		0		1				1		1		0	1			3	1			1	
354	Aggrecan core protein OS=Homo sapiens OX=9606 GN=ACAN PE=1 SV=3	ACAN	261 kDa													0		0	0	0	0			1	1			2	4	1	1	2		4	4		
355	Actin-related protein 2/3 complex subunit 2 OS=Homo sapiens OX=9606 GN=ARPC2 PE=1 SV=1	ARPC2	34 kDa													0	5	2	5	2	2	5	5	1				14	15	1	1	1	1	10	8	7	8
356	Vimentin OS=Homo sapiens OX=9606 GN=VIM PE=1 SV=4	VIM	54 kDa	27	27			5	8			39	33			0				6	5							5	6	15	17	4	4	20	20	14	16
357	Basement membrane-specific heparan sulfate proteoglycan core protein OS=Homo sapiens OX=9606 GN=HSPG2 PE=1 SV=4	HSPG2	469 kDa	12	12	30	26	7	12			8	7			0	5		7	4	7				1		1	1	4			2	1	3	6		
358	Thyroxine-binding globulin OS=Homo sapiens OX=9606 GN=SERPINA7 PE=1 SV=2	SERPINA7	46 kDa									1	1			0		0	0	0	2	0				0		0	1			1	1	1	0		
359	Clathrin heavy chain 1 OS=Homo sapiens OX=9606 GN=CLTC PE=1 SV=5	CLTC	192 kDa	5	5				0			2	2			0	1	1	3	0		3	8	1	0			6	11	0		1	1	1	0	1	
360	Retinol-binding protein 3 OS=Homo sapiens OX=9606 GN=RBP3 PE=1 SV=2	RBP3	135 kDa	169	169	227	211	122	130	21	100	206	227	42	21														0								
361	Keratin, type II cytoskeletal 5 OS=Homo sapiens OX=9606 GN=KRT5 PE=1 SV=3	KRT5	62 kDa	59	59	54	25	77	56	64	63	43	46	86	66		5		4	6	8	6		4		2		7	8	28	31		8	6	16	42	9
362	Keratin, type II cytoskeletal 6C OS=Homo sapiens OX=9606 GN=KRT6C PE=1 SV=3	KRT6C	60 kDa			48		65		72	58	43	47	81																							
363	C-reactive protein OS=Homo sapiens OX=9606 GN=CRP PE=1 SV=1	CRP	25 kDa															249	268	3	2			1		5	3	1	1					1	1	4	2
364	Thrombospondin-2 OS=Homo sapiens OX=9606 GN=THBS2 PE=1 SV=2	THBS2	130 kDa															11	22														23				
365	Pregnancy zone protein OS=Homo sapiens OX=9606 GN=PZP PE=1 SV=4	PZP	164 kDa																	30	35							29	33	19	17			20	22		
366	Opticin OS=Homo sapiens OX=9606 GN=OPTC PE=1 SV=1	OPTC	37 kDa	40	40	58	51	27	27	22	32	46	57	23	24																						
367	Dickkopf-related protein 3 OS=Homo sapiens OX=9606 GN=DKK3 PE=1 SV=2	DKK3	38 kDa	35	35	53	43	19	20	29	32	29	32	35	30		1	1	1		1				1						0	0	1		0		
368	Calsyntenin-1 OS=Homo sapiens OX=9606 GN=CLSTN1 PE=1 SV=1	CLSTN1	110 kDa	33	33	54	55	22	32	5	20	20	27	11	7			0			1				1			0	1	2			1	0	1		
369	Tubulin beta-4B chain OS=Homo sapiens OX=9606 GN=TUBB4B PE=1 SV=1	TUBB4B	50 kDa	9	9							9	9				7	9	14	5	12	14	12	8	6			25	22	15	12		6	32	30	25	14
370	Amyloid-like protein 2 OS=Homo sapiens OX=9606 GN=APLP2 PE=1 SV=2	APLP2	87 kDa	13	13	57	43	23	25	3	8	4	3	7	8							4	6	3	3			2	1	2	2	3	2	5	4	1	
371	Cathepsin D OS=Homo sapiens OX=9606 GN=CTSD PE=1 SV=1	CTSD	45 kDa	31	31	32	30	14	23	13	19	7	8	11	13									0													0
372	Protein S100-A9 OS=Homo sapiens OX=9606 GN=S100A9 PE=1 SV=1	S100A9	13 kDa	7	7	3	3	2	2	6	6	4	2	10	8		0	2	2	6	6			1				8	9	13	12	6	4	27	26	26	20
373	14-3-3 protein gamma OS=Homo sapiens OX=9606 GN=YWHAG PE=1 SV=2	YWHAG	28 kDa	3	3			1	3			10	8				5	7	6	4	5	9	14	5	3	3	4	14	14	14	14	7	5	14	15	16	16
374	Radixin OS=Homo sapiens OX=9606 GN=RDX PE=1 SV=1	RDX	69 kDa															8					6		5				21			14		17			
375	Keratin, type II cytoskeletal 4 OS=Homo sapiens OX=9606 GN=KRT4 PE=1 SV=4	KRT4	57 kDa			26				22	18	5	8	15											3												
376	Lactotransferrin OS=Homo sapiens OX=9606 GN=LTF PE=1 SV=6	LTF	78 kDa			1	1	7	1			30	32					8	9	5	13			2	2			45	46			10	10		0		
377	Alpha-actinin-4 OS=Homo sapiens OX=9606 GN=ACTN4 PE=1 SV=2	ACTN4	105 kDa	7	7																	21	29					14	10					20	20		
378	Hemoglobin subunit gamma-1 OS=Homo sapiens OX=9606 GN=HBG1 PE=1 SV=2	HBG1	16 kDa					19	19									10	12									9	8	12	9						
379	L-lactate dehydrogenase A chain OS=Homo sapiens OX=9606 GN=LDHA PE=1 SV=2	LDHA	37 kDa	22	22			1	2			6	4	1	1		2	5	6	1	1	7	9	3	2			14	13	4	4	1		8	11	11	8
380	Tenascin-X OS=Homo sapiens OX=9606 GN=TNXB PE=1 SV=5	TNXB	458 kDa														12		38	17	24							1	11	1	2	2		14	30	2	
381	Cystatin-C OS=Homo sapiens OX=9606 GN=CST3 PE=1 SV=1	CST3	16 kDa	16	16	17	13	12	13	15	15	10	9	9	10																						
382	Prostaglandin-H2 D-isomerase OS=Homo sapiens OX=9606 GN=PTGDS PE=1 SV=1	PTGDS	21 kDa	13	13	12	16	9	11	10	11	8	6	11	13																						
383	Hornerin OS=Homo sapiens OX=9606 GN=HRNR PE=1 SV=2	HRNR	282 kDa	20	20	11	3	11	12	9	12	6	8	3	4		1		1		2	1						1	1	7	2	1				10	
384	Desmoplakin OS=Homo sapiens OX=9606 GN=DSP PE=1 SV=3	DSP	332 kDa	22	22	6	1	7	17	12	12	1	2	9	7																					1	
385	Acidic leucine-rich nuclear phosphoprotein 32 family member B OS=Homo sapiens OX=9606 GN=ANP32B PE=1 SV=1	ANP32B	29 kDa									2	1	0				1		5	6							7	10	18	18	2	2	16	19	12	14
386	Keratin, type II cytoskeletal 80 OS=Homo sapiens OX=9606 GN=KRT80 PE=1 SV=2	KRT80	51 kDa				7				13		5	13									0														
387	Tropomyosin alpha-3 chain OS=Homo sapiens OX=9606 GN=TPM3 PE=1 SV=2	TPM3	33 kDa	2	2				2			3					4	4	5		3	7	12	5			3	4	10	2	3	3	2	4	4	3	6
388	Desmoglein-1 OS=Homo sapiens OX=9606 GN=DSG1 PE=1 SV=2	DSG1	114 kDa	17	17	4	1	14	6	6	13	3	4	13	11															1	0					1	
389	Glutathione peroxidase 3 OS=Homo sapiens OX=9606 GN=GPX3 PE=1 SV=2	GPX3	26 kDa	8	8	10	7	6	7	8	9	12	10	9	11		0											1				1					
390	Ras GTPase-activating-like protein IQGAP2 OS=Homo sapiens OX=9606 GN=IQGAP2 PE=1 SV=4	IQGAP2	181 kDa																1			4	3					11	9	14	13		1	17	14	10	10
391	EH domain-containing protein 3 OS=Homo sapiens OX=9606 GN=EHD3 PE=1 SV=2	EHD3	61 kDa														1	4	4	1		5	9					12	8					14	18	7	5
392	Heat shock 70 kDa protein 1A OS=Homo sapiens OX=9606 GN=HSPA1A PE=1 SV=1	HSPA1A	70 kDa	4	4			5	9									3				4	5		2			5	6		4	2	1	3	7	4	3
393	Keratin, type II cytoskeletal 78 OS=Homo sapiens OX=9606 GN=KRT78 PE=1 SV=2	KRT78	57 kDa	6	6	4		7	6	5	9		2	8	7																					2	
394	Latent-transforming growth factor beta-binding protein 2 OS=Homo sapiens OX=9606 GN=LTBP2 PE=1 SV=3	LTBP2	195 kDa	6	6	13	10	3	7		1	25	26	1	1																						
395	14-3-3 protein sigma OS=Homo sapiens OX=9606 GN=SFN PE=1 SV=1	SFN	28 kDa					1	4																												
396	Ras-related protein Rab-8B OS=Homo sapiens OX=9606 GN=RAB8B PE=1 SV=2	RAB8B	24 kDa																8									9	11					9	7	6	
397	Cytoplasmic FMR1-interacting protein 1 OS=Homo sapiens OX=9606 GN=CYFIP1 PE=1 SV=1	CYFIP1	145 kDa														2	2	5	2	4	3	4					16	20	2	2	2	2	6	10	5	7
398	Osteopontin OS=Homo sapiens OX=9606 GN=SPP1 PE=1 SV=1	SPP1	35 kDa	2	2	19	15	5	8	2	5	16	14	7	8																				1		
399	Agrin OS=Homo sapiens OX=9606 GN=AGRN PE=1 SV=6	AGRN	217 kDa	0	0	10	7		1			34	35						1			0					0					0					
400	Myocilin OS=Homo sapiens OX=9606 GN=MYOC PE=1 SV=2	MYOC	57 kDa	3	3	10	5	4	6	4	7	22	16	2	3																						
401	Peroxiredoxin-6 OS=Homo sapiens OX=9606 GN=PRDX6 PE=1 SV=3	PRDX6	25 kDa	5	5			6	7			1	2					1	2		0	3	5	1	1	1		9	7	4	2	1		4	7	2	2
402	C3 and PZP-like alpha-2-macroglobulin domain-containing protein 8 OS=Homo sapiens OX=9606 GN=CPAMD8 PE=1 SV=2	CPAMD8	207 kDa	12	12	17	14	3	6	3	7	2	3	4	1																						
403	Acidic leucine-rich nuclear phosphoprotein 32 family member A OS=Homo sapiens OX=9606 GN=ANP32A PE=1 SV=1	ANP32A	29 kDa									1								2	2							3	6	12	10	1	1	16	16	5	6
404	Leukocyte elastase inhibitor OS=Homo sapiens OX=9606 GN=SERPINB1 PE=1 SV=1	SERPINB1	43 kDa														0			2	2	2	5					10	8	6	5	1		14	10	11	7
405	Serum amyloid A-2 protein OS=Homo sapiens OX=9606 GN=SAA2 PE=1 SV=1	SAA2	14 kDa									1	1					33	39	1	1											3	2				
406	Glycogen [starch] synthase, muscle OS=Homo sapiens OX=9606 GN=GYS1 PE=1 SV=2	GYS1	84 kDa																	7	6							13	13			4	2	6	5	9	8
407	Peroxiredoxin-1 OS=Homo sapiens OX=9606 GN=PRDX1 PE=1 SV=1	PRDX1	22 kDa	3	3			8	7			1		2				3	2			2		2				6	4	3	3			3	5	2	
408	Calpain-1 catalytic subunit OS=Homo sapiens OX=9606 GN=CAPN1 PE=1 SV=1	CAPN1	82 kDa					1	3								1	2	3			3	6	1		1	1	10	8	2	2			6	7	6	5
409	Azurocidin OS=Homo sapiens OX=9606 GN=AZU1 PE=1 SV=3	AZU1	27 kDa															1	2	3	5							6	5	3	2	6	2	5	4	8	5
410	Syntaxin-binding protein 2 OS=Homo sapiens OX=9606 GN=STXBP2 PE=1 SV=2	STXBP2	66 kDa															1	2			3	6					14	11	5	2			7	6	6	5
411	High mobility group protein B1 OS=Homo sapiens OX=9606 GN=HMGB1 PE=1 SV=3	HMGB1	25 kDa									6	3								1							1		7	6			19	24	3	6
412	Amyloid-like protein 1 OS=Homo sapiens OX=9606 GN=APLP1 PE=1 SV=3	APLP1	72 kDa	8	8	19	16	6	8		3	1	1	1	1						0																
413	Ras-related C3 botulinum toxin substrate 1 OS=Homo sapiens OX=9606 GN=RAC1 PE=1 SV=1	RAC1	21 kDa																4									10	9					5	8	3	
414	Ras GTPase-activating protein 3 OS=Homo sapiens OX=9606 GN=RASA3 PE=1 SV=3	RASA3	96 kDa														1	1	1									18	14	2	1			8	9	8	3
415	Calmodulin-1 OS=Homo sapiens OX=9606 GN=CALM1 PE=1 SV=1	CALM1	17 kDa	1	1			1		1		1		2	1		1	3	2	2	3	1	1		1			5	5	6	7	2	3	5	9	3	3
416	Carboxypeptidase E OS=Homo sapiens OX=9606 GN=CPE PE=1 SV=1	CPE	53 kDa	9	9	8	6	4	6	7	8			6	4																						
417	Beta-1,4-glucuronyltransferase 1 OS=Homo sapiens OX=9606 GN=B4GAT1 PE=1 SV=1	B4GAT1	47 kDa	8	8	12	7	5	8	2	4	1	2	4	2																						
418	Elongation factor 1-alpha 1 OS=Homo sapiens OX=9606 GN=EEF1A1 PE=1 SV=1	EEF1A1	50 kDa	8	8			1	1			8	11						1			1	0					1	5	1	2			5	2	4	2
419	Purine nucleoside phosphorylase OS=Homo sapiens OX=9606 GN=PNP PE=1 SV=2	PNP	32 kDa					4	4								1	1	2	1	2	1	4					16	10	2	1			6	7	3	
420	Cytoplasmic FMR1-interacting protein 2 OS=Homo sapiens OX=9606 GN=CYFIP2 PE=1 SV=2	CYFIP2	148 kDa																		5							9	12								
421	Gamma-enolase OS=Homo sapiens OX=9606 GN=ENO2 PE=1 SV=3	ENO2	47 kDa									2	2									3															
422	Semenogelin-1 OS=Homo sapiens OX=9606 GN=SEMG1 PE=1 SV=2	SEMG1	52 kDa					67	1																												
423	Major vault protein OS=Homo sapiens OX=9606 GN=MVP PE=1 SV=4	MVP	99 kDa									1	3							8	5							19	15	1		4	3	0		1	
424	Beta-2-microglobulin OS=Homo sapiens OX=9606 GN=B2M PE=1 SV=1	B2M	14 kDa			1				5	6	3	4	3	6		2	1	2	1	2		2					4	4	1	2	1		3	4	5	2
425	Transforming growth factor beta-1 proprotein OS=Homo sapiens OX=9606 GN=TGFB1 PE=1 SV=2	TGFB1	44 kDa																0		1	4	7	5	6	3	3	6	4	3		3	3	2	1	3	3
426	Serum amyloid A-1 protein OS=Homo sapiens OX=9606 GN=SAA1 PE=1 SV=1	SAA1	14 kDa									4	2					24	25													3	2				
427	F-actin-capping protein subunit alpha-1 OS=Homo sapiens OX=9606 GN=CAPZA1 PE=1 SV=3	CAPZA1	33 kDa										1					1	1	1		2	5	0				7	5	3	1			9	8	9	5
428	Importin-7 OS=Homo sapiens OX=9606 GN=IPO7 PE=1 SV=1	IPO7	120 kDa																1			2	2	0		0		7	8	8	7	2	1	9	5	1	2
429	Guanine nucleotide-binding protein G(i) subunit alpha OS=Homo sapiens OX=9606 GN=GNAI3 PE=1 SV=3	GNAI3	41 kDa																									9	12	3	2					3	
430	Ras-related protein Rab-14 OS=Homo sapiens OX=9606 GN=RAB14 PE=1 SV=4	RAB14	24 kDa																4			3	6					5	7	1	2		1	4	6	2	2
431	Neutral alpha-glucosidase AB OS=Homo sapiens OX=9606 GN=GANAB PE=1 SV=3	GANAB	107 kDa										1				1	8	7	1	0	3	7	6	5	1	1	0				3	3				
432	Junction plakoglobin OS=Homo sapiens OX=9606 GN=JUP PE=1 SV=3	JUP	82 kDa	4	4	2	0	2	4	2	4	0	0	4	0		0	0	0		0	0	0	0	0	0	0			1	0					2	0
433	Tyrosine-protein kinase Lyn OS=Homo sapiens OX=9606 GN=LYN PE=1 SV=3	LYN	59 kDa														1	1	2		1		2	1				6	5	1	1			5	7	7	8
434	Guanine nucleotide-binding protein subunit alpha-13 OS=Homo sapiens OX=9606 GN=GNA13 PE=1 SV=2	GNA13	44 kDa															2				1	2					8	10	2				6	7	4	6
435	Intercellular adhesion molecule 2 OS=Homo sapiens OX=9606 GN=ICAM2 PE=1 SV=2	ICAM2	31 kDa															2				3	4				1	6	8	4	3			7	8	3	3
436	Glucose-6-phosphate 1-dehydrogenase OS=Homo sapiens OX=9606 GN=G6PD PE=1 SV=4	G6PD	59 kDa																			1	1					4	3	5	4			9	11	7	5
437	Actin-related protein 2 OS=Homo sapiens OX=9606 GN=ACTR2 PE=1 SV=1	ACTR2	45 kDa																2	0	1		1					13	9					11	10	4	4
438	Chitinase-3-like protein 1 OS=Homo sapiens OX=9606 GN=CHI3L1 PE=1 SV=2	CHI3L1	43 kDa	1	1					2	2	22	23																								
439	Lysozyme C OS=Homo sapiens OX=9606 GN=LYZ PE=1 SV=1	LYZ	17 kDa	0	0	1	2	1		2	1	13	9	3	2									1				2	1	1	1						1
440	cAMP-dependent protein kinase type I-alpha regulatory subunit OS=Homo sapiens OX=9606 GN=PRKAR1A PE=1 SV=1	PRKAR1A	43 kDa																3			2	3					9	9	2	1			9	7	3	4
441	Tyrosine-protein kinase Fyn OS=Homo sapiens OX=9606 GN=FYN PE=1 SV=3	FYN	61 kDa																3				3					7	7					5	7	6	4
442	Rab GDP dissociation inhibitor beta OS=Homo sapiens OX=9606 GN=GDI2 PE=1 SV=2	GDI2	51 kDa					1										3	1			4	5	1	2			7	7	2	1	1		3	2	6	2
443	Actin-related protein 2/3 complex subunit 4 OS=Homo sapiens OX=9606 GN=ARPC4 PE=1 SV=3	ARPC4	20 kDa														3	2	2	1	3	3	5	2	1			7	5	2	2			5	5	5	3
444	Flotillin-1 OS=Homo sapiens OX=9606 GN=FLOT1 PE=1 SV=3	FLOT1	47 kDa	0	0	0	0					0	0					2	3	1	1	2	2	1	1	0	0	4	4	0	1		0	1	0	0	0
445	Aquaporin-1 OS=Homo sapiens OX=9606 GN=AQP1 PE=1 SV=3	AQP1	29 kDa	1	1				1								1	3	3		1	3	7	4	2	2	2	6	5	1	4			1	2	1	
446	Wnt inhibitory factor 1 OS=Homo sapiens OX=9606 GN=WIF1 PE=1 SV=3	WIF1	42 kDa	7	7	9	9	4	6	1	2				2																						
447	Myosin-10 OS=Homo sapiens OX=9606 GN=MYH10 PE=1 SV=3	MYH10	229 kDa	3	3																											1		3	2		
448	60S acidic ribosomal protein P0 OS=Homo sapiens OX=9606 GN=RPLP0 PE=1 SV=1	RPLP0	34 kDa									4	6															1	2	8	5			8	8	3	2
449	Alpha-amylase 1 OS=Homo sapiens OX=9606 GN=AMY1A PE=1 SV=2	AMY1A	58 kDa								1	21	24																	1	1						
450	55 kDa erythrocyte membrane protein OS=Homo sapiens OX=9606 GN=MPP1 PE=1 SV=2	MPP1	52 kDa														0	4	2			3	3	1	1	1	1	8	8	1	1			2	4	2	1
451	Semenogelin-2 OS=Homo sapiens OX=9606 GN=SEMG2 PE=1 SV=1	SEMG2	65 kDa					45					0													0	0										
452	(E3-independent) E2 ubiquitin-conjugating enzyme OS=Homo sapiens OX=9606 GN=UBE2O PE=1 SV=3	UBE2O	141 kDa															1	1			4	1					4	4	6	1	2	2	4	7	2	1
453	Out at first protein homolog OS=Homo sapiens OX=9606 GN=OAF PE=2 SV=1	OAF	31 kDa	4	4	11	11	2	2	1	2	4	5	1	1																						
454	Nucleolin OS=Homo sapiens OX=9606 GN=NCL PE=1 SV=3	NCL	77 kDa	1	1							8	12																	3	4			4	5	1	0
455	Disintegrin and metalloproteinase domain-containing protein 10 OS=Homo sapiens OX=9606 GN=ADAM10 PE=1 SV=1	ADAM10	84 kDa															0	1	0			1					9	8	0	1		0	4	4	1	2
456	Ras-related protein Rab-6B OS=Homo sapiens OX=9606 GN=RAB6B PE=1 SV=1	RAB6B	23 kDa																				4					4	5					5	4		
457	Src substrate cortactin OS=Homo sapiens OX=9606 GN=CTTN PE=1 SV=2	CTTN	62 kDa														0	1	1		0		2				0	6	5					6	8	4	5
458	Ubiquitin-like modifier-activating enzyme 1 OS=Homo sapiens OX=9606 GN=UBA1 PE=1 SV=3	UBA1	118 kDa	11	11				1										1			2	1					6	3					2	2		
459	Low-density lipoprotein receptor-related protein 2 OS=Homo sapiens OX=9606 GN=LRP2 PE=1 SV=3	LRP2	522 kDa	3	3	12	10	1	3			1	1				0										0										0
460	Cathepsin G OS=Homo sapiens OX=9606 GN=CTSG PE=1 SV=2	CTSG	29 kDa									1	1						1	1	1							4	3	3	2			2	2	6	4
461	Nucleosome assembly protein 1-like 4 OS=Homo sapiens OX=9606 GN=NAP1L4 PE=1 SV=1	NAP1L4	43 kDa																2									2	1	8	6		2	7	6	3	2
462	Guanine nucleotide-binding protein G(z) subunit alpha OS=Homo sapiens OX=9606 GN=GNAZ PE=1 SV=3	GNAZ	41 kDa																0			1	2	0				7	5	2	2			3	3	5	4
463	Immunoglobulin lambda variable 3-10 OS=Homo sapiens OX=9606 GN=IGLV3-10 PE=3 SV=2	IGLV3-10	12 kDa			1	0			1	0	2	0				2				1	2	2	2	2	1	2	1	1	1	1	1		1	1	2	3
464	Protein SET OS=Homo sapiens OX=9606 GN=SET PE=1 SV=3	SET	33 kDa									2	1							1	0							2	1	5	6			3	5	5	5
465	Nardilysin OS=Homo sapiens OX=9606 GN=NRDC PE=1 SV=3	NRDC	132 kDa																									1	1	4	5			6	6	4	6
466	ATP synthase subunit alpha, mitochondrial OS=Homo sapiens OX=9606 GN=ATP5F1A PE=1 SV=1	ATP5F1A	60 kDa	15	15																	2	5	1													
467	Retinoschisin OS=Homo sapiens OX=9606 GN=RS1 PE=1 SV=2	RS1	26 kDa	3	3	3	4	1	2	1	2	3	4		1																						
468	Dermcidin OS=Homo sapiens OX=9606 GN=DCD PE=1 SV=2	DCD	11 kDa	4	4	3	7	4	2	4	3	0		2	6																						
469	Insulin-like growth factor-binding protein 6 OS=Homo sapiens OX=9606 GN=IGFBP6 PE=1 SV=1	IGFBP6	25 kDa	3	3	3	4	2	4	3	6	3	2	2	2																						
470	Receptor-type tyrosine-protein phosphatase eta OS=Homo sapiens OX=9606 GN=PTPRJ PE=1 SV=3	PTPRJ	146 kDa																2									5	9		0			5	7	5	2
471	Guanine nucleotide-binding protein subunit beta-4 OS=Homo sapiens OX=9606 GN=GNB4 PE=1 SV=3	GNB4	38 kDa																									6	5								3
472	Glutathione S-transferase P OS=Homo sapiens OX=9606 GN=GSTP1 PE=1 SV=2	GSTP1	23 kDa	5	5			2	1			2							1		0	1	2					2	3	1	1			2	2	2	1
473	F-actin-capping protein subunit beta OS=Homo sapiens OX=9606 GN=CAPZB PE=1 SV=4	CAPZB	31 kDa									1	0									1	1	1				6	4	1	1			4	5	3	3
474	Src kinase-associated phosphoprotein 2 OS=Homo sapiens OX=9606 GN=SKAP2 PE=1 SV=1	SKAP2	41 kDa															1	1			2	2					2	2	1	1			6	8	2	3
475	Nucleobindin-1 OS=Homo sapiens OX=9606 GN=NUCB1 PE=1 SV=4	NUCB1	54 kDa	3	3	5	3		2	2	3	7	4	1	1																						
476	Flavin reductase (NADPH) OS=Homo sapiens OX=9606 GN=BLVRB PE=1 SV=3	BLVRB	22 kDa					15	14									1		1								1	1					1	1		
477	Neutrophil defensin 1 OS=Homo sapiens OX=9606 GN=DEFA1 PE=1 SV=1	DEFA1	10 kDa						1			1	1							1	1							3	2	2	1	3	1	1	2	6	6
478	Filaggrin-2 OS=Homo sapiens OX=9606 GN=FLG2 PE=1 SV=1	FLG2	248 kDa	5	5	2	2	2	3	3	3	1	2	4	3															1						1	
479	Golgi membrane protein 1 OS=Homo sapiens OX=9606 GN=GOLM1 PE=1 SV=1	GOLM1	45 kDa			0	0					2	6	0	0			2	1	3	3				0			1						6	3		
480	Prothymosin alpha OS=Homo sapiens OX=9606 GN=PTMA PE=1 SV=2	PTMA	12 kDa																									2	1	7	8			5	5	4	5
481	Aldehyde dehydrogenase, dimeric NADP-preferring OS=Homo sapiens OX=9606 GN=ALDH3A1 PE=1 SV=3	ALDH3A1	50 kDa	9	9			5	5		1	1	2		1																						
482	Selenoprotein P OS=Homo sapiens OX=9606 GN=SELENOP PE=1 SV=3	SELENOP	43 kDa	1	1														1		2	4	4	2	2			1	2	2	1	4	2	3	2	1	
483	Prolargin OS=Homo sapiens OX=9606 GN=PRELP PE=1 SV=1	PRELP	44 kDa	1	1	7	4	0	1	1	2	1	2	2	2																						
484	Serine/threonine-protein kinase 24 OS=Homo sapiens OX=9606 GN=STK24 PE=1 SV=1	STK24	49 kDa																									5	4	0	0			2	4	3	2
485	Acidic leucine-rich nuclear phosphoprotein 32 family member E OS=Homo sapiens OX=9606 GN=ANP32E PE=1 SV=1	ANP32E	31 kDa																	1	1							2	2	5	4			4	7	5	4
486	Ras-related protein Rab-7a OS=Homo sapiens OX=9606 GN=RAB7A PE=1 SV=1	RAB7A	23 kDa																1			4	6					4	3		1			1	4	2	2
487	Decorin OS=Homo sapiens OX=9606 GN=DCN PE=1 SV=1	DCN	40 kDa	10	10	1	1	2	3		1	3	3																								
488	Nidogen-2 OS=Homo sapiens OX=9606 GN=NID2 PE=1 SV=3	NID2	151 kDa			1													1	0		3	3	2	2	0		2	3					2	0	1	
489	Glutathione S-transferase omega-1 OS=Homo sapiens OX=9606 GN=GSTO1 PE=1 SV=2	GSTO1	28 kDa					0	0													1	1					7	5	1				3	2	2	3
490	Immunoglobulin delta heavy chain OS=Homo sapiens OX=9606 PE=1 SV=1	IgD	56 kDa															8	6		0	1	1	2				0	1	1	1		2	2		1	
491	Growth arrest-specific protein 6 OS=Homo sapiens OX=9606 GN=GAS6 PE=1 SV=3	GAS6	75 kDa			8	5	2	3			4	4	2			0		1																	1	
492	Ras GTPase-activating-like protein IQGAP1 OS=Homo sapiens OX=9606 GN=IQGAP1 PE=1 SV=1	IQGAP1	189 kDa									3	4															3	0	3	3			3	5	1	1
493	Calcium-binding protein 39 OS=Homo sapiens OX=9606 GN=CAB39 PE=1 SV=1	CAB39	40 kDa														1		1									4	5		1			2	4	4	3
494	Neural cell adhesion molecule L1-like protein OS=Homo sapiens OX=9606 GN=CHL1 PE=1 SV=4	CHL1	135 kDa	0	0	4	4		3			6	6													0					0		0				0
495	Target of Nesh-SH3 OS=Homo sapiens OX=9606 GN=ABI3BP PE=1 SV=1	ABI3BP	119 kDa	2	2	6	4					2	3					1	1	1								1	1					2	2		
496	Cystatin-A OS=Homo sapiens OX=9606 GN=CSTA PE=1 SV=1	CSTA	11 kDa	3	3	3	2	4	1	4	2			2	5																						
497	F-actin-capping protein subunit alpha-2 OS=Homo sapiens OX=9606 GN=CAPZA2 PE=1 SV=3	CAPZA2	33 kDa																			1	4	0				3	3	1	0			4	6	4	2
498	Spondin-1 OS=Homo sapiens OX=9606 GN=SPON1 PE=1 SV=2	SPON1	91 kDa	4	4	5	3		1		3	2	0	0																							
499	General vesicular transport factor p115 OS=Homo sapiens OX=9606 GN=USO1 PE=1 SV=2	USO1	108 kDa																	1								1	1		0			5	10	6	3
500	High mobility group protein B2 OS=Homo sapiens OX=9606 GN=HMGB2 PE=1 SV=2	HMGB2	24 kDa									1	2								1							1	1	3	6	1		2	3	1	5
501	Transketolase OS=Homo sapiens OX=9606 GN=TKT PE=1 SV=3	TKT	68 kDa	4	4				2			1	1				0		0		1							2	2	0				1		2	0
502	Ubiquitin carboxyl-terminal hydrolase 15 OS=Homo sapiens OX=9606 GN=USP15 PE=1 SV=3	USP15	112 kDa																				1					4	7	1	2	0		4	3		1
503	Caveolae-associated protein 2 OS=Homo sapiens OX=9606 GN=CAVIN2 PE=1 SV=3	CAVIN2	47 kDa															1	3		1	3	3	2	2			1	2	1	1			1		1	1
504	Transaldolase OS=Homo sapiens OX=9606 GN=TALDO1 PE=1 SV=2	TALDO1	38 kDa						2													1	2	1	1		1	3	3		1			1	1	2	2
505	Desmocollin-1 OS=Homo sapiens OX=9606 GN=DSC1 PE=1 SV=2	DSC1	100 kDa	2	2			3	1	2	4	1	1	3	4															1						1	
506	Ras-related protein Ral-B OS=Homo sapiens OX=9606 GN=RALB PE=1 SV=1	RALB	23 kDa															1	2		1		1	0				2	2	2	1			2	2	5	0
507	Acid ceramidase OS=Homo sapiens OX=9606 GN=ASAH1 PE=1 SV=5	ASAH1	45 kDa	4	4	5	4	1	1	1	2			1	1																						
508	T-complex protein 1 subunit epsilon OS=Homo sapiens OX=9606 GN=CCT5 PE=1 SV=1	CCT5	60 kDa																				2					2	2	2	4			1	3	3	1
509	Annexin A5 OS=Homo sapiens OX=9606 GN=ANXA5 PE=1 SV=2	ANXA5	36 kDa	1	1																							2	2	1	2			3	5	1	
510	Secretogranin-3 OS=Homo sapiens OX=9606 GN=SCG3 PE=1 SV=3	SCG3	53 kDa	2	2	10	8				1	1	1																								
511	Elongation factor 1-gamma OS=Homo sapiens OX=9606 GN=EEF1G PE=1 SV=3	EEF1G	50 kDa	1	1							2	1									2	2					2	2	1	1			3	3	2	2
512	Prostaglandin E synthase 3 OS=Homo sapiens OX=9606 GN=PTGES3 PE=1 SV=1	PTGES3	19 kDa																1	1	1		1	1				3	3	4	2	0		3	4	1	
513	Twinfilin-2 OS=Homo sapiens OX=9606 GN=TWF2 PE=1 SV=2	TWF2	40 kDa														1	1	1			2	2					4	4					3	5	4	
514	Sulfhydryl oxidase 1 OS=Homo sapiens OX=9606 GN=QSOX1 PE=1 SV=3	QSOX1	83 kDa			4	3					5	3				1						1		0			0		1		1	1				
515	Cullin-associated NEDD8-dissociated protein 1 OS=Homo sapiens OX=9606 GN=CAND1 PE=1 SV=2	CAND1	136 kDa						1				0										1					1	3	3	2			4	6	2	
516	Integrin beta-2 OS=Homo sapiens OX=9606 GN=ITGB2 PE=1 SV=2	ITGB2	85 kDa															2		0	0							6	6	1	0	1	2				
517	Thioredoxin OS=Homo sapiens OX=9606 GN=TXN PE=1 SV=3	TXN	12 kDa	2	2		0	3	1	2	2			2	2							1						1	1	1					1		
518	Dynactin subunit 1 OS=Homo sapiens OX=9606 GN=DCTN1 PE=1 SV=3	DCTN1	142 kDa															0	1	1	2							3	3	2	2			2	2	2	2
519	Microtubule-associated protein RP/EB family member 2 OS=Homo sapiens OX=9606 GN=MAPRE2 PE=1 SV=1	MAPRE2	37 kDa																1				1	1				4	1	1				3	2	2	2
520	Tripeptidyl-peptidase 1 OS=Homo sapiens OX=9606 GN=TPP1 PE=1 SV=2	TPP1	61 kDa	5	5	5	4	2	3		1			1																							
521	Nck-associated protein 1 OS=Homo sapiens OX=9606 GN=NCKAP1 PE=1 SV=1	NCKAP1	129 kDa																									3	5					1	5	3	3
522	Angiopoietin-related protein 6 OS=Homo sapiens OX=9606 GN=ANGPTL6 PE=1 SV=1	ANGPTL6	52 kDa														1	1	2	1	1		2					1		2	3	1		5	1	1	1
523	Synaptosomal-associated protein 23 OS=Homo sapiens OX=9606 GN=SNAP23 PE=1 SV=1	SNAP23	23 kDa															1				1	1					3	3	2	1			3	2	1	1
524	Testican-1 OS=Homo sapiens OX=9606 GN=SPOCK1 PE=1 SV=1	SPOCK1	49 kDa	1	1	8	4		1	0	0	2	4																								
525	Retinal dehydrogenase 1 OS=Homo sapiens OX=9606 GN=ALDH1A1 PE=1 SV=2	ALDH1A1	55 kDa					10	12																									0	1		
526	Hsc70-interacting protein OS=Homo sapiens OX=9606 GN=ST13 PE=1 SV=2	ST13	41 kDa	1	1			2	3			1	1					1	1			1	2			1		1	1	0				2	2		
527	Cathelicidin antimicrobial peptide OS=Homo sapiens OX=9606 GN=CAMP PE=1 SV=1	CAMP	19 kDa															1	4	1	2							4	4	1	1	1	1		1		1
528	Ras-related protein Rab-11B OS=Homo sapiens OX=9606 GN=RAB11B PE=1 SV=4	RAB11B	24 kDa																			2	6					2	2	1	0	1		1	3	0	
529	Transferrin receptor protein 1 OS=Homo sapiens OX=9606 GN=TFRC PE=1 SV=2	TFRC	85 kDa														1	0	1			3	2					1	1	3	3			0	0		
530	Ran GTPase-activating protein 1 OS=Homo sapiens OX=9606 GN=RANGAP1 PE=1 SV=1	RANGAP1	64 kDa															0			1							1	1	3	4			3	3	2	2
531	Pappalysin-2 OS=Homo sapiens OX=9606 GN=PAPPA2 PE=1 SV=4	PAPPA2	199 kDa			4	2					6	8																								
532	T-complex protein 1 subunit gamma OS=Homo sapiens OX=9606 GN=CCT3 PE=1 SV=4	CCT3	61 kDa																				0					2	1	2	2			2	5	1	1
533	ATPase ASNA1 OS=Homo sapiens OX=9606 GN=ASNA1 PE=1 SV=2	ASNA1	39 kDa															1		1								0		4	2			3	4	2	1
534	Integrin alpha-M OS=Homo sapiens OX=9606 GN=ITGAM PE=1 SV=2	ITGAM	127 kDa																									5	9	1		4	2				1
535	Seizure protein 6 homolog OS=Homo sapiens OX=9606 GN=SEZ6 PE=1 SV=2	SEZ6	107 kDa			7	8		2			3	1																								
536	Receptor-type tyrosine-protein phosphatase C OS=Homo sapiens OX=9606 GN=PTPRC PE=1 SV=3	PTPRC	147 kDa														1		1									8	7						1		
537	SH3 domain-binding glutamic acid-rich-like protein 3 OS=Homo sapiens OX=9606 GN=SH3BGRL3 PE=1 SV=1	SH3BGRL3	10 kDa														1	1	1		1	1	1					2	4	1	1	1	0	1	1	1	1
538	Myosin light polypeptide 6 OS=Homo sapiens OX=9606 GN=MYL6 PE=1 SV=2	MYL6	17 kDa									3	2				1		0			1	1	2	2			1	2	1	1			1	2		1
539	Cysteine and glycine-rich protein 1 OS=Homo sapiens OX=9606 GN=CSRP1 PE=1 SV=3	CSRP1	21 kDa																1			2	2	0	1			2	2	0				2	3	3	1
540	Ribonuclease inhibitor OS=Homo sapiens OX=9606 GN=RNH1 PE=1 SV=2	RNH1	50 kDa	1	1			2	1													0	1		0			1	0	2				3	2	0	0
541	Thymidine phosphorylase OS=Homo sapiens OX=9606 GN=TYMP PE=1 SV=2	TYMP	50 kDa									4	7										1					2	2					1	1	1	1
542	Coatomer subunit gamma-1 OS=Homo sapiens OX=9606 GN=COPG1 PE=1 SV=1	COPG1	98 kDa																	0	0							2	3	2	2			2	2	3	2
543	Eukaryotic translation initiation factor 3 subunit D OS=Homo sapiens OX=9606 GN=EIF3D PE=1 SV=1	EIF3D	64 kDa																									2	3	2	2			2	1	2	2
544	Adipocyte enhancer-binding protein 1 OS=Homo sapiens OX=9606 GN=AEBP1 PE=1 SV=1	AEBP1	131 kDa			1						7	7				1		1	1	0																
545	Kinesin-like protein KIF2A OS=Homo sapiens OX=9606 GN=KIF2A PE=1 SV=3	KIF2A	80 kDa																									1	2	0				6	4	1	1
546	Clusterin-like protein 1 OS=Homo sapiens OX=9606 GN=CLUL1 PE=2 SV=1	CLUL1	54 kDa	2	2	4	4					2	3																								
547	Rho-related GTP-binding protein RhoG OS=Homo sapiens OX=9606 GN=RHOG PE=1 SV=1	RHOG	21 kDa																	0								3	3	1	1						0
548	Matrix metalloproteinase-9 OS=Homo sapiens OX=9606 GN=MMP9 PE=1 SV=3	MMP9	78 kDa															0										7	7			3				0	
549	Nucleophosmin OS=Homo sapiens OX=9606 GN=NPM1 PE=1 SV=2	NPM1	33 kDa									2	1					1		1	1							2	2	2	3	1	1	1	1		2
550	cAMP-dependent protein kinase catalytic subunit beta OS=Homo sapiens OX=9606 GN=PRKACB PE=1 SV=2	PRKACB	41 kDa															1				2	1					4	1	1	1			2	2	1	1
551	Lipocalin-1 OS=Homo sapiens OX=9606 GN=LCN1 PE=1 SV=1	LCN1	19 kDa	1	1	2	2	1	2		2	4	2		1																						
552	Prolactin-inducible protein OS=Homo sapiens OX=9606 GN=PIP PE=1 SV=1	PIP	17 kDa			1	1	2	1	1	1	5	3	0	2																						
553	Kalirin OS=Homo sapiens OX=9606 GN=KALRN PE=1 SV=3	KALRN	340 kDa															0	0			1	2					2	2	1				2	2	2	3
554	Coactosin-like protein OS=Homo sapiens OX=9606 GN=COTL1 PE=1 SV=3	COTL1	16 kDa																				1	1				4	2	1				1	2	2	1
555	P-selectin OS=Homo sapiens OX=9606 GN=SELP PE=1 SV=3	SELP	91 kDa																			5	4							2	1			2	1	1	1
556	Heterogeneous nuclear ribonucleoprotein U OS=Homo sapiens OX=9606 GN=HNRNPU PE=1 SV=6	HNRNPU	91 kDa									3	3																	2	1			3	3	3	1
557	Caspase-14 OS=Homo sapiens OX=9606 GN=CASP14 PE=1 SV=2	CASP14	28 kDa	1	1			1		3	2	1		6	5																						
558	Rho GTPase-activating protein 18 OS=Homo sapiens OX=9606 GN=ARHGAP18 PE=1 SV=3	ARHGAP18	75 kDa																									3	3					0	4	0	
559	Heterogeneous nuclear ribonucleoprotein U-like protein 2 OS=Homo sapiens OX=9606 GN=HNRNPUL2 PE=1 SV=1	HNRNPUL2	85 kDa																											1	2			5	5	0	
560	Proteasome subunit alpha type-6 OS=Homo sapiens OX=9606 GN=PSMA6 PE=1 SV=1	PSMA6	27 kDa	1	1			1	2			3	3				1		1			1						1		1	1				1	1	0
561	Metalloproteinase inhibitor 1 OS=Homo sapiens OX=9606 GN=TIMP1 PE=1 SV=1	TIMP1	23 kDa	1	1	1	1	1	1	1	1	4	4	1	1																						
562	Syntaxin-11 OS=Homo sapiens OX=9606 GN=STX11 PE=1 SV=1	STX11	33 kDa															1				1	2					3	4	1	1			1	1	2	1
563	Protein-glutamine gamma-glutamyltransferase E OS=Homo sapiens OX=9606 GN=TGM3 PE=1 SV=4	TGM3	77 kDa	1	1	1		2	1	0	1	1	3	2	0																					1	
564	Secreted frizzled-related protein 3 OS=Homo sapiens OX=9606 GN=FRZB PE=1 SV=2	FRZB	36 kDa	3	3	3	2		1	1	2			1	1																						
565	C-type lectin domain family 1 member B OS=Homo sapiens OX=9606 GN=CLEC1B PE=1 SV=2	CLEC1B	27 kDa																				1					3	4	1	0			3	1	2	1
566	Serine/threonine-protein phosphatase PP1-alpha catalytic subunit OS=Homo sapiens OX=9606 GN=PPP1CA PE=1 SV=1	PPP1CA	38 kDa																			1	1					4	0					3	2	3	2
567	A-kinase anchor protein 12 OS=Homo sapiens OX=9606 GN=AKAP12 PE=1 SV=4	AKAP12	191 kDa														0		2	1	4					0		1	1					2	0		
568	Tryptophan--tRNA ligase, cytoplasmic OS=Homo sapiens OX=9606 GN=WARS PE=1 SV=2	WARS	53 kDa									8	8																								
569	Apolipoprotein A-V OS=Homo sapiens OX=9606 GN=APOA5 PE=1 SV=1	APOA5	41 kDa														1	0	4	1	2							1		1		1	1			2	1
570	CD226 antigen OS=Homo sapiens OX=9606 GN=CD226 PE=1 SV=2	CD226	39 kDa																				1					3	3	1	0			3	2	2	0
571	Glia maturation factor gamma OS=Homo sapiens OX=9606 GN=GMFG PE=1 SV=1	GMFG	17 kDa																				0					2	2	0	0			1	1	2	1
572	Serine/threonine-protein kinase TAO3 OS=Homo sapiens OX=9606 GN=TAOK3 PE=1 SV=2	TAOK3	105 kDa																									5	2				0	5	4	1	1
573	Na(+)/H(+) exchange regulatory cofactor NHE-RF1 OS=Homo sapiens OX=9606 GN=SLC9A3R1 PE=1 SV=4	SLC9A3R1	39 kDa															0										2	6					3	3		
574	Rho GDP-dissociation inhibitor 2 OS=Homo sapiens OX=9606 GN=ARHGDIB PE=1 SV=3	ARHGDIB	23 kDa																		1	2	0	1	1			2	2	1	1			1	2	1	2
575	Neuronal cell adhesion molecule OS=Homo sapiens OX=9606 GN=NRCAM PE=1 SV=3	NRCAM	144 kDa			4	2		3			5	2																							1	
576	Choline transporter-like protein 1 OS=Homo sapiens OX=9606 GN=SLC44A1 PE=1 SV=1	SLC44A1	73 kDa																									3	4			1		4	2	0	
577	Alpha-synuclein OS=Homo sapiens OX=9606 GN=SNCA PE=1 SV=1	SNCA	14 kDa					7	8																				1		0						
578	Ras-related protein Rap-2b OS=Homo sapiens OX=9606 GN=RAP2B PE=1 SV=1	RAP2B	21 kDa																			1	1	1				2	1	2	2	1		1	1	2	1
579	Lymphocyte antigen 6 complex locus protein G6f OS=Homo sapiens OX=9606 GN=LY6G6F PE=1 SV=2	LY6G6F	32 kDa															0	1			1	1	0	1			1	2	1				2	2	1	1
580	T-complex protein 1 subunit delta OS=Homo sapiens OX=9606 GN=CCT4 PE=1 SV=4	CCT4	58 kDa										0					0	1			1	1					2	2	1	2	0		1	1	1	
581	Annexin A1 OS=Homo sapiens OX=9606 GN=ANXA1 PE=1 SV=2	ANXA1	39 kDa			1		1	2					1						2								2	2			1				1	
582	6-phosphogluconate dehydrogenase, decarboxylating OS=Homo sapiens OX=9606 GN=PGD PE=1 SV=3	PGD	53 kDa	1	1			1	2														1					2	3					1	0		1
583	Syndecan-4 OS=Homo sapiens OX=9606 GN=SDC4 PE=1 SV=2	SDC4	22 kDa																	1	1	2	1	2	1				0		1			2	2		
584	Phosphatidylinositol 5-phosphate 4-kinase type-2 alpha OS=Homo sapiens OX=9606 GN=PIP4K2A PE=1 SV=2	PIP4K2A	46 kDa						1									1										3	3	1	1			2	2	1	
585	Bridging integrator 2 OS=Homo sapiens OX=9606 GN=BIN2 PE=1 SV=3	BIN2	62 kDa															1	0			2	2	1	1					1	1					1	2
586	Alpha-adducin OS=Homo sapiens OX=9606 GN=ADD1 PE=1 SV=2	ADD1	81 kDa														1	2	1				1					5	3					2	1	0	
587	Tyrosine-protein phosphatase non-receptor type 6 OS=Homo sapiens OX=9606 GN=PTPN6 PE=1 SV=1	PTPN6	68 kDa																				0					3	4	0	0	0		1	1	1	2
588	Arachidonate 12-lipoxygenase, 12S-type OS=Homo sapiens OX=9606 GN=ALOX12 PE=1 SV=4	ALOX12	76 kDa																			0	2					2	3					3	3		1
589	Follistatin-related protein 1 OS=Homo sapiens OX=9606 GN=FSTL1 PE=1 SV=1	FSTL1	35 kDa			4	5					4	3																								
590	Carbonic anhydrase 3 OS=Homo sapiens OX=9606 GN=CA3 PE=1 SV=3	CA3	30 kDa	6	6			0	1																												
591	S-arrestin OS=Homo sapiens OX=9606 GN=SAG PE=1 SV=3	SAG	45 kDa									6	8																								
592	Immunoglobulin lambda variable 2-11 OS=Homo sapiens OX=9606 GN=IGLV2-11 PE=1 SV=2	IGLV2-11	13 kDa																															1	2		
593	GTP-binding nuclear protein Ran OS=Homo sapiens OX=9606 GN=RAN PE=1 SV=3	RAN	24 kDa	1	1			1	1			1											1					2	2	1	1			1	1	1	0
594	Serpin B3 OS=Homo sapiens OX=9606 GN=SERPINB3 PE=1 SV=2	SERPINB3	45 kDa	1	1		1	1		1	1	1	1	2	1																						
595	Extracellular superoxide dismutase [Cu-Zn] OS=Homo sapiens OX=9606 GN=SOD3 PE=1 SV=2	SOD3	26 kDa			2	1					3	0				1			1				1					2					1	2		
596	Importin subunit alpha-3 OS=Homo sapiens OX=9606 GN=KPNA4 PE=1 SV=1	KPNA4	58 kDa																									1	1	1	3			2	2	2	1
597	Protein flightless-1 homolog OS=Homo sapiens OX=9606 GN=FLII PE=1 SV=2	FLII	145 kDa																			1							1	2	1			2	1	2	3
598	Elongation factor 1-delta OS=Homo sapiens OX=9606 GN=EEF1D PE=1 SV=5	EEF1D	31 kDa									1	1															1	0	1	1			3	5	1	
599	E3 ubiquitin-protein ligase UBR4 OS=Homo sapiens OX=9606 GN=UBR4 PE=1 SV=1	UBR4	574 kDa			0																	1						2	2	2			3	1	3	
600	Destrin OS=Homo sapiens OX=9606 GN=DSTN PE=1 SV=3	DSTN	19 kDa														1											3	2					2	4	2	1
601	Growth factor receptor-bound protein 2 OS=Homo sapiens OX=9606 GN=GRB2 PE=1 SV=1	GRB2	25 kDa																			0						2	2					1	1	1	0
602	ProSAAS OS=Homo sapiens OX=9606 GN=PCSK1N PE=1 SV=1	PCSK1N	27 kDa	2	2	5	5					2																									
603	Bcl-2-like protein 13 OS=Homo sapiens OX=9606 GN=BCL2L13 PE=1 SV=1	BCL2L13	53 kDa	6	6																																
604	Protein diaphanous homolog 1 OS=Homo sapiens OX=9606 GN=DIAPH1 PE=1 SV=2	DIAPH1	141 kDa																	0								2	2	1	1			1	1	2	1
605	Protein S100-A4 OS=Homo sapiens OX=9606 GN=S100A4 PE=1 SV=1	S100A4	12 kDa	1	1			2	3														1	0					0	1						1	1
606	Glucose-6-phosphate isomerase OS=Homo sapiens OX=9606 GN=GPI PE=1 SV=4	GPI	63 kDa																			1	1					2	2					1	1	2	2
607	Nck-associated protein 1-like OS=Homo sapiens OX=9606 GN=NCKAP1L PE=1 SV=3	NCKAP1L	128 kDa																	1								6	2			1	2	1	1	1	
608	Fibrillin-1 OS=Homo sapiens OX=9606 GN=FBN1 PE=1 SV=4	FBN1	312 kDa	1	1	2	1					2	1									1	0	0	0					0	0						
609	Alpha-centractin OS=Homo sapiens OX=9606 GN=ACTR1A PE=1 SV=1	ACTR1A	43 kDa																			2	3					3	1	1				0	1	1	
610	Serpin B6 OS=Homo sapiens OX=9606 GN=SERPINB6 PE=1 SV=3	SERPINB6	43 kDa																			0						3	1	1	0			3	1	3	1
611	Microtubule-associated protein RP/EB family member 1 OS=Homo sapiens OX=9606 GN=MAPRE1 PE=1 SV=3	MAPRE1	30 kDa																									1	1	2	2			2	1	1	
612	Retbindin OS=Homo sapiens OX=9606 GN=RTBDN PE=1 SV=2	RTBDN	25 kDa	1	1	4	3		2			3	1																								
613	Inter-alpha-trypsin inhibitor heavy chain H5 OS=Homo sapiens OX=9606 GN=ITIH5 PE=2 SV=2	ITIH5	105 kDa	1	1	6	3	1	1				1																								
614	116 kDa U5 small nuclear ribonucleoprotein component OS=Homo sapiens OX=9606 GN=EFTUD2 PE=1 SV=1	EFTUD2	109 kDa																									0	2	4	1				1	2	1
615	Neuroserpin OS=Homo sapiens OX=9606 GN=SERPINI1 PE=1 SV=1	SERPINI1	46 kDa	2	2	4	3		0			1																									
616	Tetraspanin-33 OS=Homo sapiens OX=9606 GN=TSPAN33 PE=1 SV=1	TSPAN33	32 kDa																1									4	2					1	3	1	
617	Protein kinase C and casein kinase substrate in neurons protein 2 OS=Homo sapiens OX=9606 GN=PACSIN2 PE=1 SV=2	PACSIN2	56 kDa																			1	1	0				3						2	3	0	
618	Sodium/potassium-transporting ATPase subunit alpha-1 OS=Homo sapiens OX=9606 GN=ATP1A1 PE=1 SV=1	ATP1A1	113 kDa	6	6																							1	1					0			
619	T-complex protein 1 subunit alpha OS=Homo sapiens OX=9606 GN=TCP1 PE=1 SV=1	TCP1	60 kDa																									3	3					4	2		0
620	Microfibril-associated glycoprotein 4 OS=Homo sapiens OX=9606 GN=MFAP4 PE=1 SV=2	MFAP4	29 kDa	2	2	5	4																														
621	Guanylate cyclase soluble subunit beta-1 OS=Homo sapiens OX=9606 GN=GUCY1B1 PE=1 SV=1	GUCY1B1	71 kDa																			0	0	0	0	0	0			0	0					1	2
622	Myc target protein 1 OS=Homo sapiens OX=9606 GN=MYCT1 PE=1 SV=1	MYCT1	27 kDa															1				1	1					2	1	1				2	2	1	2
623	Proteasome subunit alpha type-5 OS=Homo sapiens OX=9606 GN=PSMA5 PE=1 SV=3	PSMA5	26 kDa	1	1			2				2	2									1						1		1	2				1		
624	Plexin domain-containing protein 2 OS=Homo sapiens OX=9606 GN=PLXDC2 PE=1 SV=1	PLXDC2	60 kDa									0						2				1	1	1	1		1	1				2	2				
625	Ras-related protein Rab-5C OS=Homo sapiens OX=9606 GN=RAB5C PE=1 SV=2	RAB5C	23 kDa																1				2					2	2					2	1	2	1
626	Lymphocyte cytosolic protein 2 OS=Homo sapiens OX=9606 GN=LCP2 PE=1 SV=1	LCP2	60 kDa															1	1		1							2	2				0	2	2	1	
627	Proteasome subunit beta type-6 OS=Homo sapiens OX=9606 GN=PSMB6 PE=1 SV=4	PSMB6	25 kDa	2	2			2	3										0										1	1	1					0	
628	Splicing factor 3B subunit 3 OS=Homo sapiens OX=9606 GN=SF3B3 PE=1 SV=4	SF3B3	136 kDa																									2	0	2	2			1	2	2	1
629	Heterogeneous nuclear ribonucleoprotein K OS=Homo sapiens OX=9606 GN=HNRNPK PE=1 SV=1	HNRNPK	51 kDa	3	3							2	1															0						1		1	
630	Dynamin-1-like protein OS=Homo sapiens OX=9606 GN=DNM1L PE=1 SV=2	DNM1L	82 kDa															1				2	4					2	1							1	
631	T-complex protein 1 subunit eta OS=Homo sapiens OX=9606 GN=CCT7 PE=1 SV=2	CCT7	59 kDa																				1					2	2		0			0	1	0	
632	Myeloperoxidase OS=Homo sapiens OX=9606 GN=MPO PE=1 SV=1	MPO	84 kDa																	1								3	3			2	3				
633	Unconventional myosin-Ig OS=Homo sapiens OX=9606 GN=MYO1G PE=1 SV=2	MYO1G	116 kDa																									4	4	1						1	0
634	Galectin-1 OS=Homo sapiens OX=9606 GN=LGALS1 PE=1 SV=2	LGALS1	15 kDa	1	1							5	3																								
635	Cytosolic non-specific dipeptidase OS=Homo sapiens OX=9606 GN=CNDP2 PE=1 SV=2	CNDP2	53 kDa	5	5																							1	1								
636	Alpha-aminoadipic semialdehyde dehydrogenase OS=Homo sapiens OX=9606 GN=ALDH7A1 PE=1 SV=5	ALDH7A1	58 kDa	5	5																																
637	Proteasome subunit beta type-3 OS=Homo sapiens OX=9606 GN=PSMB3 PE=1 SV=2	PSMB3	23 kDa						2			3	1					1					1	1		1		1	1						1		
638	Annexin A7 OS=Homo sapiens OX=9606 GN=ANXA7 PE=1 SV=3	ANXA7	53 kDa														1	1	1					1				1	2	1				2	1		
639	T-complex protein 1 subunit beta OS=Homo sapiens OX=9606 GN=CCT2 PE=1 SV=4	CCT2	57 kDa						1														2					1	2	1	1			1	1	1	
640	Tetraspanin-14 OS=Homo sapiens OX=9606 GN=TSPAN14 PE=1 SV=1	TSPAN14	31 kDa																1		1		1					2	2				1	2	2	0	
641	Follistatin-related protein 5 OS=Homo sapiens OX=9606 GN=FSTL5 PE=2 SV=2	FSTL5	96 kDa	1	1	3	2	0	3			1																									
642	V-type proton ATPase subunit S1 OS=Homo sapiens OX=9606 GN=ATP6AP1 PE=1 SV=2	ATP6AP1	52 kDa			3		2	2			0	2	1	2																						
643	Secretogranin-1 OS=Homo sapiens OX=9606 GN=CHGB PE=1 SV=2	CHGB	78 kDa	1	1	6	3	1					1																								
644	Rho GTPase-activating protein 45 OS=Homo sapiens OX=9606 GN=ARHGAP45 PE=1 SV=2	ARHGAP45	125 kDa																			0		0	0	0		0	1		1			0	3	1	0
645	T-complex protein 1 subunit theta OS=Homo sapiens OX=9606 GN=CCT8 PE=1 SV=4	CCT8	60 kDa																									1	2		1			0	3	0	
646	Iduronate 2-sulfatase OS=Homo sapiens OX=9606 GN=IDS PE=1 SV=1	IDS	62 kDa	0	0	4	2		2																												
647	Reelin OS=Homo sapiens OX=9606 GN=RELN PE=1 SV=3	RELN	388 kDa			4	4		1								0		0																		
648	Coatomer subunit alpha OS=Homo sapiens OX=9606 GN=COPA PE=1 SV=2	COPA	138 kDa																											0				4	4	0	1
649	Voltage-dependent anion-selective channel protein 3 OS=Homo sapiens OX=9606 GN=VDAC3 PE=1 SV=1	VDAC3	31 kDa																			4	3	1													
650	Stress-70 protein, mitochondrial OS=Homo sapiens OX=9606 GN=HSPA9 PE=1 SV=2	HSPA9	74 kDa	6	6																																
651	Proteasome subunit alpha type-7 OS=Homo sapiens OX=9606 GN=PSMA7 PE=1 SV=1	PSMA7	28 kDa	1	1				1			1	2														1		1		1				1		
652	Plasminogen activator inhibitor 1 OS=Homo sapiens OX=9606 GN=SERPINE1 PE=1 SV=1	SERPINE1	45 kDa															1	1									2	2	1	0			1	1	1	
653	Limbic system-associated membrane protein OS=Homo sapiens OX=9606 GN=LSAMP PE=1 SV=2	LSAMP	37 kDa	1	1	2	2		2		1	1	1																								
654	Delta-aminolevulinic acid dehydratase OS=Homo sapiens OX=9606 GN=ALAD PE=1 SV=1	ALAD	36 kDa					2	3								1		1				1					2	1						1		
655	Claudin-5 OS=Homo sapiens OX=9606 GN=CLDN5 PE=2 SV=1	CLDN5	23 kDa																			1	1					1	0		1			0	2	1	2
656	Seizure 6-like protein OS=Homo sapiens OX=9606 GN=SEZ6L PE=1 SV=1	SEZ6L	112 kDa			2	0					1								0	1		0		1				1	1				1			
657	High affinity immunoglobulin epsilon receptor subunit gamma OS=Homo sapiens OX=9606 GN=FCER1G PE=1 SV=1	FCER1G	10 kDa																1									2	1		0			2	3	1	1
658	Galectin-related protein OS=Homo sapiens OX=9606 GN=LGALSL PE=1 SV=2	LGALSL	19 kDa																1				0					2	3					1	1	0	1
659	Serine/threonine-protein phosphatase 6 catalytic subunit OS=Homo sapiens OX=9606 GN=PPP6C PE=1 SV=1	PPP6C	35 kDa																									1	1	1	2			0	2	1	
660	Peroxiredoxin-5, mitochondrial OS=Homo sapiens OX=9606 GN=PRDX5 PE=1 SV=4	PRDX5	22 kDa	3	3							1																0	1						0	1	
661	Adenylate kinase isoenzyme 1 OS=Homo sapiens OX=9606 GN=AK1 PE=1 SV=3	AK1	22 kDa					2	2														1					3	1						1		
662	Serine/threonine-protein kinase TAO1 OS=Homo sapiens OX=9606 GN=TAOK1 PE=1 SV=1	TAOK1	116 kDa																	0	0							3						2	1	2	1
663	Poly(rC)-binding protein 1 OS=Homo sapiens OX=9606 GN=PCBP1 PE=1 SV=2	PCBP1	37 kDa																				1					3	1					3	2		
664	Phosphatidylethanolamine-binding protein 1 OS=Homo sapiens OX=9606 GN=PEBP1 PE=1 SV=3	PEBP1	21 kDa	2	2				0																			1	3						0		
665	Protein-arginine deiminase type-2 OS=Homo sapiens OX=9606 GN=PADI2 PE=1 SV=2	PADI2	76 kDa																			0	0	0	0	0	0							1	2	1	
666	Ganglioside GM2 activator OS=Homo sapiens OX=9606 GN=GM2A PE=1 SV=4	GM2A	21 kDa			2	1					3	2																								
667	Protein-glutamine gamma-glutamyltransferase 2 OS=Homo sapiens OX=9606 GN=TGM2 PE=1 SV=2	TGM2	77 kDa					3	9																												
668	26S proteasome regulatory subunit 8 OS=Homo sapiens OX=9606 GN=PSMC5 PE=1 SV=1	PSMC5	46 kDa														1		1				1					1	1	1				1	2	1	1
669	Chloride intracellular channel protein 4 OS=Homo sapiens OX=9606 GN=CLIC4 PE=1 SV=4	CLIC4	29 kDa															1	1					1	1			2	2					1	1		1
670	EGF-containing fibulin-like extracellular matrix protein 2 OS=Homo sapiens OX=9606 GN=EFEMP2 PE=1 SV=3	EFEMP2	49 kDa	1	1	2	3		1	1	1	1																									
671	Choline transporter-like protein 2 OS=Homo sapiens OX=9606 GN=SLC44A2 PE=1 SV=3	SLC44A2	80 kDa																				1					2	1			1			1	2	1
672	Eukaryotic initiation factor 4A-I OS=Homo sapiens OX=9606 GN=EIF4A1 PE=1 SV=1	EIF4A1	46 kDa	0	0								1															1	1	1				2	1		
673	Keratocan OS=Homo sapiens OX=9606 GN=KERA PE=1 SV=1	KERA	41 kDa	2	2	1	1	1	2																												
674	Claudin-3 OS=Homo sapiens OX=9606 GN=CLDN3 PE=1 SV=1	CLDN3	23 kDa																			1						2	2	1				1	1		
675	Inverted formin-2 OS=Homo sapiens OX=9606 GN=INF2 PE=1 SV=2	INF2	136 kDa									0											1			0		2	2					3		1	
676	Galectin-7 OS=Homo sapiens OX=9606 GN=LGALS7 PE=1 SV=2	LGALS7	15 kDa	3	3					3				1	1																						
677	Protein disulfide-isomerase A6 OS=Homo sapiens OX=9606 GN=PDIA6 PE=1 SV=1	PDIA6	48 kDa										1									3	5	1	1												
678	Equilibrative nucleoside transporter 1 OS=Homo sapiens OX=9606 GN=SLC29A1 PE=1 SV=3	SLC29A1	50 kDa															2	0									3	4						1		
679	Phosphoglucomutase-1 OS=Homo sapiens OX=9606 GN=PGM1 PE=1 SV=3	PGM1	61 kDa																									4	5					1			1
680	Renin receptor OS=Homo sapiens OX=9606 GN=ATP6AP2 PE=1 SV=2	ATP6AP2	39 kDa			2	0					4	4																								
681	Aflatoxin B1 aldehyde reductase member 2 OS=Homo sapiens OX=9606 GN=AKR7A2 PE=1 SV=3	AKR7A2	40 kDa					1														1	1					2	1					1	1	1	0
682	Glia-derived nexin OS=Homo sapiens OX=9606 GN=SERPINE2 PE=1 SV=1	SERPINE2	44 kDa																			1		3					1	1		1	1	1	1		
683	Transforming growth factor beta-1-induced transcript 1 protein OS=Homo sapiens OX=9606 GN=TGFB1I1 PE=1 SV=2	TGFB1I1	50 kDa																									2	1					2	2	2	1
684	Ubiquitin-like-conjugating enzyme ATG3 OS=Homo sapiens OX=9606 GN=ATG3 PE=1 SV=1	ATG3	36 kDa																									1	1	1	1			2	3		
685	Attractin OS=Homo sapiens OX=9606 GN=ATRN PE=1 SV=2	ATRN	159 kDa																	2	1	1							1					1	1		
686	Nucleoside diphosphate kinase A OS=Homo sapiens OX=9606 GN=NME1 PE=1 SV=1	NME1	17 kDa	1	1												1		1									3	0						1		
687	Peptidyl-prolyl cis-trans isomerase B OS=Homo sapiens OX=9606 GN=PPIB PE=1 SV=2	PPIB	24 kDa																0			2	1		0				1	1	1						
688	Glycogenin-1 OS=Homo sapiens OX=9606 GN=GYG1 PE=1 SV=4	GYG1	39 kDa																	1	0							3	2			1				1	
689	E3 ubiquitin-protein ligase HUWE1 OS=Homo sapiens OX=9606 GN=HUWE1 PE=1 SV=3	HUWE1	482 kDa														0												1	0	0			2	1	0	
690	Myotrophin OS=Homo sapiens OX=9606 GN=MTPN PE=1 SV=2	MTPN	13 kDa																									3	3					1	3		
691	Dihydropyrimidinase-related protein 3 OS=Homo sapiens OX=9606 GN=DPYSL3 PE=1 SV=1	DPYSL3	62 kDa	3	3							1	1																								
692	Vascular cell adhesion protein 1 OS=Homo sapiens OX=9606 GN=VCAM1 PE=1 SV=1	VCAM1	81 kDa									4	5																								
693	Protein MGARP OS=Homo sapiens OX=9606 GN=MGARP PE=1 SV=1	MGARP	25 kDa	5	5																																
694	Unconventional myosin-If OS=Homo sapiens OX=9606 GN=MYO1F PE=1 SV=3	MYO1F	125 kDa																									2	1	1	1					2	1
695	60S acidic ribosomal protein P2 OS=Homo sapiens OX=9606 GN=RPLP2 PE=1 SV=1	RPLP2	12 kDa									1																	1	3	2			1	1		
696	Eukaryotic translation initiation factor 5A-1 OS=Homo sapiens OX=9606 GN=EIF5A PE=1 SV=2	EIF5A	17 kDa					1																				0	2					0	1	1	
697	Collagen alpha-2(IX) chain OS=Homo sapiens OX=9606 GN=COL9A2 PE=1 SV=2	COL9A2	65 kDa	1	1	4	1		1																												
698	Insulin-like growth factor-binding protein 7 OS=Homo sapiens OX=9606 GN=IGFBP7 PE=1 SV=1	IGFBP7	29 kDa	1	1	2	1			0	0	0	1																								
699	Low affinity immunoglobulin gamma Fc region receptor III-B OS=Homo sapiens OX=9606 GN=FCGR3B PE=1 SV=2	FCGR3B	26 kDa																	1								2	3			1	2				
700	Proteasome subunit alpha type-4 OS=Homo sapiens OX=9606 GN=PSMA4 PE=1 SV=1	PSMA4	29 kDa									1	2										1					2								1	
701	Proteasome subunit alpha type-2 OS=Homo sapiens OX=9606 GN=PSMA2 PE=1 SV=2	PSMA2	26 kDa	1	1							4	2																		1						
702	Protein-arginine deiminase type-4 OS=Homo sapiens OX=9606 GN=PADI4 PE=1 SV=2	PADI4	74 kDa																															2	1	3	1
703	Lymphocyte-specific protein 1 OS=Homo sapiens OX=9606 GN=LSP1 PE=1 SV=1	LSP1	37 kDa									1	1																		0			3	2		
704	ATP-dependent 6-phosphofructokinase, platelet type OS=Homo sapiens OX=9606 GN=PFKP PE=1 SV=2	PFKP	86 kDa	2	2																		1					1						0			
705	Testican-2 OS=Homo sapiens OX=9606 GN=SPOCK2 PE=1 SV=1	SPOCK2	47 kDa	0	0	2	1	0	2																												
706	Superoxide dismutase [Mn], mitochondrial OS=Homo sapiens OX=9606 GN=SOD2 PE=1 SV=3	SOD2	25 kDa														1					2	3		1												
707	Tenascin-R OS=Homo sapiens OX=9606 GN=TNR PE=1 SV=3	TNR	150 kDa			4	2					1	2																								
708	ATP-citrate synthase OS=Homo sapiens OX=9606 GN=ACLY PE=1 SV=3	ACLY	121 kDa																									2	3		0			1	2		
709	Laminin subunit gamma-1 OS=Homo sapiens OX=9606 GN=LAMC1 PE=1 SV=3	LAMC1	178 kDa									2	2							1		1			1							1					
710	Protein DDI1 homolog 2 OS=Homo sapiens OX=9606 GN=DDI2 PE=1 SV=1	DDI2	45 kDa					1	1									1										1	2					0	1		
711	T-complex protein 1 subunit zeta OS=Homo sapiens OX=9606 GN=CCT6A PE=1 SV=3	CCT6A	58 kDa																				1					1			1			1	2	1	
712	ADP-ribosylation factor 6 OS=Homo sapiens OX=9606 GN=ARF6 PE=1 SV=2	ARF6	20 kDa																									1	2						2	1	1
713	Programmed cell death protein 10 OS=Homo sapiens OX=9606 GN=PDCD10 PE=1 SV=1	PDCD10	25 kDa																									1	1						2	2	2
714	Cadherin-related family member 1 OS=Homo sapiens OX=9606 GN=CDHR1 PE=1 SV=2	CDHR1	94 kDa	1	1	0						1	4																								
715	Phosphoglycerate mutase 1 OS=Homo sapiens OX=9606 GN=PGAM1 PE=1 SV=2	PGAM1	29 kDa	2	2																							1	2	0							
716	Serine protease HTRA1 OS=Homo sapiens OX=9606 GN=HTRA1 PE=1 SV=1	HTRA1	51 kDa			2	1					1	2																								
717	Receptor-type tyrosine-protein phosphatase alpha OS=Homo sapiens OX=9606 GN=PTPRA PE=1 SV=3	PTPRA	91 kDa																									1	1					3	2		0
718	Serine/threonine-protein kinase MRCK beta OS=Homo sapiens OX=9606 GN=CDC42BPB PE=1 SV=2	CDC42BPB	194 kDa																									1	3					1	1		
719	Rho GTPase-activating protein 1 OS=Homo sapiens OX=9606 GN=ARHGAP1 PE=1 SV=1	ARHGAP1	50 kDa																				0					2	1	0				0		1	
720	Protein-L-isoaspartate(D-aspartate) O-methyltransferase OS=Homo sapiens OX=9606 GN=PCMT1 PE=1 SV=4	PCMT1	25 kDa																									2	1								1
721	60 kDa heat shock protein, mitochondrial OS=Homo sapiens OX=9606 GN=HSPD1 PE=1 SV=2	HSPD1	61 kDa	3	3																																
722	Cellular retinoic acid-binding protein 1 OS=Homo sapiens OX=9606 GN=CRABP1 PE=1 SV=2	CRABP1	16 kDa	4	4																																
723	Serpin E3 OS=Homo sapiens OX=9606 GN=SERPINE3 PE=2 SV=2	SERPINE3	47 kDa			5	3																														
724	Superoxide dismutase [Cu-Zn] OS=Homo sapiens OX=9606 GN=SOD1 PE=1 SV=2	SOD1	16 kDa					4	4																												
725	Neuroendocrine convertase 2 OS=Homo sapiens OX=9606 GN=PCSK2 PE=1 SV=2	PCSK2	71 kDa			4	3																														
726	Coatomer subunit beta' OS=Homo sapiens OX=9606 GN=COPB2 PE=1 SV=2	COPB2	102 kDa																															2			0
727	Myosin light chain kinase, smooth muscle OS=Homo sapiens OX=9606 GN=MYLK PE=1 SV=4	MYLK	211 kDa																				2														
728	Four and a half LIM domains protein 1 OS=Homo sapiens OX=9606 GN=FHL1 PE=1 SV=4	FHL1	36 kDa																1				1					2	1					1	1		
729	Elongation factor 1-beta OS=Homo sapiens OX=9606 GN=EEF1B2 PE=1 SV=3	EEF1B2	25 kDa	1	1							2	2																						1		
730	Proprotein convertase subtilisin/kexin type 6 OS=Homo sapiens OX=9606 GN=PCSK6 PE=1 SV=1	PCSK6	106 kDa																			1	2	2	1			1									
731	Casein kinase II subunit alpha OS=Homo sapiens OX=9606 GN=CSNK2A1 PE=1 SV=1	CSNK2A1	45 kDa																									2		1	0			0	1	1	
732	Heterogeneous nuclear ribonucleoproteins C1/ C2 OS=Homo sapiens OX=9606 GN=HNRNPC PE=1 SV=4	HNRNPC	34 kDa									2																			1			2	2		0
733	Proteasome subunit alpha type-1 OS=Homo sapiens OX=9606 GN=PSMA1 PE=1 SV=1	PSMA1	30 kDa					1	1			2	1											0													
734	Insulin-like growth factor-binding protein 2 OS=Homo sapiens OX=9606 GN=IGFBP2 PE=1 SV=2	IGFBP2	35 kDa			3	2					1	1																								
735	Hematopoietic lineage cell-specific protein OS=Homo sapiens OX=9606 GN=HCLS1 PE=1 SV=3	HCLS1	54 kDa									1																			1			3	2		
736	40S ribosomal protein S3 OS=Homo sapiens OX=9606 GN=RPS3 PE=1 SV=2	RPS3	27 kDa																											1	1				3	1	
737	Cytoplasmic protein NCK2 OS=Homo sapiens OX=9606 GN=NCK2 PE=1 SV=2	NCK2	43 kDa																									2	3					1	1		
738	PDZ and LIM domain protein 1 OS=Homo sapiens OX=9606 GN=PDLIM1 PE=1 SV=4	PDLIM1	36 kDa																			3	1	2											1		
739	Contactin-1 OS=Homo sapiens OX=9606 GN=CNTN1 PE=1 SV=1	CNTN1	113 kDa			2	2		1																										0		
740	Neurexin-3 OS=Homo sapiens OX=9606 GN=NRXN3 PE=1 SV=4	NRXN3	181 kDa			3	2						1																								
741	Voltage-dependent anion-selective channel protein 2 OS=Homo sapiens OX=9606 GN=VDAC2 PE=1 SV=2	VDAC2	32 kDa	3	3																		1														
742	RAS guanyl-releasing protein 2 OS=Homo sapiens OX=9606 GN=RASGRP2 PE=1 SV=1	RASGRP2	69 kDa																									4							1		1
743	Lamin-B1 OS=Homo sapiens OX=9606 GN=LMNB1 PE=1 SV=2	LMNB1	66 kDa			1						2	3																								
744	Coronin-1B OS=Homo sapiens OX=9606 GN=CORO1B PE=1 SV=1	CORO1B	54 kDa																				0					3	0						2		
745	Complement factor D OS=Homo sapiens OX=9606 GN=CFD PE=1 SV=5	CFD	27 kDa									3	4																								
746	Cathepsin F OS=Homo sapiens OX=9606 GN=CTSF PE=1 SV=1	CTSF	53 kDa			2	2																														
747	FH1/FH2 domain-containing protein 1 OS=Homo sapiens OX=9606 GN=FHOD1 PE=1 SV=3	FHOD1	127 kDa																									2	1								
748	Voltage-dependent anion-selective channel protein 1 OS=Homo sapiens OX=9606 GN=VDAC1 PE=1 SV=2	VDAC1	31 kDa	2	2												0					0	0														
749	Plexin-A4 OS=Homo sapiens OX=9606 GN=PLXNA4 PE=1 SV=4	PLXNA4	212 kDa																									1	2					1	1		
750	Creatine kinase B-type OS=Homo sapiens OX=9606 GN=CKB PE=1 SV=1	CKB	43 kDa	1	1							2	2																								
751	GTPase HRas OS=Homo sapiens OX=9606 GN=HRAS PE=1 SV=1	HRAS	21 kDa																									2	2					1	1		
752	Reticulon-3 OS=Homo sapiens OX=9606 GN=RTN3 PE=1 SV=2	RTN3	113 kDa																															1	1	1	2
753	GTPase-activating protein and VPS9 domain-containing protein 1 OS=Homo sapiens OX=9606 GN=GAPVD1 PE=1 SV=2	GAPVD1	165 kDa																									1		2	2				0		
754	Erbin OS=Homo sapiens OX=9606 GN=ERBIN PE=1 SV=2	ERBIN	158 kDa																			0							2					1		1	1
755	Glutathione reductase, mitochondrial OS=Homo sapiens OX=9606 GN=GSR PE=1 SV=2	GSR	56 kDa																									2	1						1		
756	Coatomer subunit beta OS=Homo sapiens OX=9606 GN=COPB1 PE=1 SV=3	COPB1	107 kDa																															3	1	1	
757	Carboxypeptidase Q OS=Homo sapiens OX=9606 GN=CPQ PE=1 SV=1	CPQ	52 kDa			4	1						1																								
758	UV excision repair protein RAD23 homolog A OS=Homo sapiens OX=9606 GN=RAD23A PE=1 SV=1	RAD23A	40 kDa					3	2									0																			
759	Protein kinase C-binding protein NELL2 OS=Homo sapiens OX=9606 GN=NELL2 PE=1 SV=1	NELL2	91 kDa			2	3					0																									
760	Malate dehydrogenase, mitochondrial OS=Homo sapiens OX=9606 GN=MDH2 PE=1 SV=3	MDH2	36 kDa																			3	1	0	0												
761	Importin-5 OS=Homo sapiens OX=9606 GN=IPO5 PE=1 SV=4	IPO5	124 kDa																									1			2				0		
762	Endonuclease domain-containing 1 protein OS=Homo sapiens OX=9606 GN=ENDOD1 PE=1 SV=2	ENDOD1	55 kDa																			2	4														
763	Aldo-keto reductase family 1 member C1 OS=Homo sapiens OX=9606 GN=AKR1C1 PE=1 SV=1	AKR1C1	37 kDa	3	3																																
764	Bisphosphoglycerate mutase OS=Homo sapiens OX=9606 GN=BPGM PE=1 SV=2	BPGM	30 kDa					3	3																												
765	Collagen alpha-2(I) chain OS=Homo sapiens OX=9606 GN=COL1A2 PE=1 SV=7	COL1A2	129 kDa	2	2																																
766	Cathepsin B OS=Homo sapiens OX=9606 GN=CTSB PE=1 SV=3	CTSB	38 kDa									4	1																								
767	Fibroblast growth factor-binding protein 2 OS=Homo sapiens OX=9606 GN=FGFBP2 PE=1 SV=1	FGFBP2	25 kDa			1						2	1																						1		
768	Neutrophil gelatinase-associated lipocalin OS=Homo sapiens OX=9606 GN=LCN2 PE=1 SV=2	LCN2	23 kDa										1															2	1	0		1					
769	Retinoic acid receptor responder protein 2 OS=Homo sapiens OX=9606 GN=RARRES2 PE=1 SV=1	RARRES2	19 kDa	1	1	2	0																														
770	Alpha-soluble NSF attachment protein OS=Homo sapiens OX=9606 GN=NAPA PE=1 SV=3	NAPA	33 kDa																										1					1	2		
771	Protein XRP2 OS=Homo sapiens OX=9606 GN=RP2 PE=1 SV=4	RP2	40 kDa																									1	2					1	0		
772	60S ribosomal protein L15 OS=Homo sapiens OX=9606 GN=RPL15 PE=1 SV=2	RPL15	24 kDa																															2	2	1	
773	Ficolin-2 OS=Homo sapiens OX=9606 GN=FCN2 PE=1 SV=2	FCN2	34 kDa																									1	2						0		
774	Chordin-like protein 1 OS=Homo sapiens OX=9606 GN=CHRDL1 PE=1 SV=2	CHRDL1	52 kDa	0	0	2	1																														
775	Inositol polyphosphate-5-phosphatase A OS=Homo sapiens OX=9606 GN=INPP5A PE=1 SV=1	INPP5A	48 kDa																									1	0						2		
776	Collagen alpha-1(VI) chain OS=Homo sapiens OX=9606 GN=COL6A1 PE=1 SV=3	COL6A1	109 kDa																	0				2				1									
777	Tyrosine-protein kinase SYK OS=Homo sapiens OX=9606 GN=SYK PE=1 SV=1	SYK	72 kDa																				0					1	3								
778	Peroxisomal multifunctional enzyme type 2 OS=Homo sapiens OX=9606 GN=HSD17B4 PE=1 SV=3	HSD17B4	80 kDa																			2	2														
779	Isocitrate dehydrogenase [NADP], mitochondrial OS=Homo sapiens OX=9606 GN=IDH2 PE=1 SV=2	IDH2	51 kDa																			2	2														
780	Glutamate receptor 4 OS=Homo sapiens OX=9606 GN=GRIA4 PE=1 SV=2	GRIA4	101 kDa			4	1																														
781	Adenosylhomocysteinase OS=Homo sapiens OX=9606 GN=AHCY PE=1 SV=4	AHCY	48 kDa					1	1																										2		
782	Beta-arrestin-1 OS=Homo sapiens OX=9606 GN=ARRB1 PE=1 SV=2	ARRB1	47 kDa																									2	1							1	
783	CD109 antigen OS=Homo sapiens OX=9606 GN=CD109 PE=1 SV=2	CD109	162 kDa																										2					1	0		
784	Metalloproteinase inhibitor 2 OS=Homo sapiens OX=9606 GN=TIMP2 PE=1 SV=2	TIMP2	24 kDa			1	2	0																													
785	Immunoglobulin lambda variable 9-49 OS=Homo sapiens OX=9606 GN=IGLV9-49 PE=1 SV=1	IGLV9-49	13 kDa																									2	1								0
786	Ubiquitin carboxyl-terminal hydrolase 14 OS=Homo sapiens OX=9606 GN=USP14 PE=1 SV=3	USP14	56 kDa					1	2																												
787	Spliceosome RNA helicase DDX39B OS=Homo sapiens OX=9606 GN=DDX39B PE=1 SV=1	DDX39B	49 kDa									2	1																								
788	Kell blood group glycoprotein OS=Homo sapiens OX=9606 GN=KEL PE=1 SV=2	KEL	83 kDa																				1						3								
789	45 kDa calcium-binding protein OS=Homo sapiens OX=9606 GN=SDF4 PE=1 SV=1	SDF4	42 kDa									1	2																								
790	Endothelial cell-selective adhesion molecule OS=Homo sapiens OX=9606 GN=ESAM PE=1 SV=1	ESAM	41 kDa																			2	0													0	
791	Prosaposin OS=Homo sapiens OX=9606 GN=PSAP PE=1 SV=2	PSAP	58 kDa			3					0																										
792	ADAM DEC1 OS=Homo sapiens OX=9606 GN=ADAMDEC1 PE=1 SV=2	ADAMDEC1	53 kDa									2	1																								
793	Platelet-activating factor acetylhydrolase OS=Homo sapiens OX=9606 GN=PLA2G7 PE=1 SV=1	PLA2G7	50 kDa									2	1																								
794	Thromboxane-A synthase OS=Homo sapiens OX=9606 GN=TBXAS1 PE=1 SV=3	TBXAS1	61 kDa																			1	2														
795	Leucine-rich repeat-containing protein 15 OS=Homo sapiens OX=9606 GN=LRRC15 PE=2 SV=2	LRRC15	64 kDa									2																								1	
796	Lysosomal alpha-glucosidase OS=Homo sapiens OX=9606 GN=GAA PE=1 SV=4	GAA	105 kDa			2	1																														
797	Peptidyl-prolyl cis-trans isomerase F, mitochondrial OS=Homo sapiens OX=9606 GN=PPIF PE=1 SV=1	PPIF	22 kDa																				2	1													
798	Eukaryotic translation initiation factor 5 OS=Homo sapiens OX=9606 GN=EIF5 PE=1 SV=2	EIF5	49 kDa																											2	1						
799	Unconventional myosin-Ic OS=Homo sapiens OX=9606 GN=MYO1C PE=1 SV=4	MYO1C	122 kDa																									0							2		
800	SH3 domain-binding glutamic acid-rich-like protein OS=Homo sapiens OX=9606 GN=SH3BGRL PE=1 SV=1	SH3BGRL	13 kDa																									2									0
801	Thyroglobulin OS=Homo sapiens OX=9606 GN=TG PE=1 SV=5	TG	305 kDa																	3																	
802	Stromal interaction molecule 1 OS=Homo sapiens OX=9606 GN=STIM1 PE=1 SV=3	STIM1	77 kDa																			2															
803	Fc receptor-like protein 5 OS=Homo sapiens OX=9606 GN=FCRL5 PE=1 SV=3	FCRL5	106 kDa									2																									

Sixty-seven proteins were not previously reported and have been described here in association with EVs for the first time (Figure 3A and Table 3). Among these 67 proteins, 50 were found in both OT and CAT groups, 10 were found only in the CAT group, and seven were found only in the OT group (Figure 3B). Additionally, among the seven proteins in the OT group, two were described only in the AH in patient 3 (Table 2). The other five proteins were found in the plasma. The protein ARHGAP45 was detected in patients 2, 4, 5, and 6, the ACAN protein was detected in patients 1, 2, and 4, and the ERBIN protein was detected in patients 2, 5, and 6 (Table 2).

**Table 3:** New proteins identified in the aqueous humor- and plasma-derived EVs.

#	Identified Proteins	Alternative Name	MW	AH		Plasma	
				CAT	OT	CAT	OT
360	Retinol-binding protein 3 OS=Homo sapiens OX=9606 GN=RBP3 PE=1 SV=2	RBP3	135 kDa	171	103	0	0
412	Amyloid-like protein 1 OS=Homo sapiens OX=9606 GN=APLP1 PE=1 SV=3	APLP1	72 kDa	11	1	0	0
417	Beta-1,4-glucuronyltransferase 1 OS=Homo sapiens OX=9606 GN=B4GAT1 PE=1 SV=1	B4GAT1	47 kDa	8	3	0	0
446	Wnt inhibitory factor 1 OS=Homo sapiens OX=9606 GN=WIF1 PE=1 SV=3	WIF1	42 kDa	7	2	0	0
510	Secretogranin-3 OS=Homo sapiens OX=9606 GN=SCG3 PE=1 SV=3	SCG3	53 kDa	6	1	0	0
693	Protein MGARP OS=Homo sapiens OX=9606 GN=MGARP PE=1 SV=1	MGARP	25 kDa	5	0	0	0
723	Serpin E3 OS=Homo sapiens OX=9606 GN=SERPINE3 PE=2 SV=2	SERPINE3	47 kDa	4	0	0	0
366	Opticin OS=Homo sapiens OX=9606 GN=OPTC PE=1 SV=1	OPTC	37 kDa	41	34	0	0
725	Neuroendocrine convertase 2 OS=Homo sapiens OX=9606 GN=PCSK2 PE=1 SV=2	PCSK2	71 kDa	4	0	0	0
707	Tenascin-R OS=Homo sapiens OX=9606 GN=TNR PE=1 SV=3	TNR	150 kDa	3	2	0	0
546	Clusterin-like protein 1 OS=Homo sapiens OX=9606 GN=CLUL1 PE=2 SV=1	CLUL1	54 kDa	3	3	0	0
467	Retinoschisin OS=Homo sapiens OX=9606 GN=RS1 PE=1 SV=2	RS1	26 kDa	3	2	0	0
780	Glutamate receptor 4 OS=Homo sapiens OX=9606 GN=GRIA4 PE=1 SV=2	GRIA4	101 kDa	3	0	0	0
759	Protein kinase C-binding protein NELL2 OS=Homo sapiens OX=9606 GN=NELL2 PE=1 SV=1	NELL2	91 kDa	3	0	0	0
740	Neurexin-3 OS=Homo sapiens OX=9606 GN=NRXN3 PE=1 SV=4	NRXN3	181 kDa	3	1	0	0
564	Secreted frizzled-related protein 3 OS=Homo sapiens OX=9606 GN=FRZB PE=1 SV=2	FRZB	36 kDa	2	1	0	0
612	Retbindin OS=Homo sapiens OX=9606 GN=RTBDN PE=1 SV=2	RTBDN	25 kDa	2	2	0	0
641	Follistatin-related protein 5 OS=Homo sapiens OX=9606 GN=FSTL5 PE=2 SV=2	FSTL5	96 kDa	2	1	0	0
646	Iduronate 2-sulfatase OS=Homo sapiens OX=9606 GN=IDS PE=1 SV=1	IDS	62 kDa	2	0	0	0
673	Keratocan OS=Homo sapiens OX=9606 GN=KERA PE=1 SV=1	KERA	41 kDa	2	0	0	0
705	Testican-2 OS=Homo sapiens OX=9606 GN=SPOCK2 PE=1 SV=1	SPOCK2	47 kDa	1	0	0	0
774	Chordin-like protein 1 OS=Homo sapiens OX=9606 GN=CHRDL1 PE=1 SV=2	CHRDL1	52 kDa	1	0	0	0
714	Cadherin-related family member 1 OS=Homo sapiens OX=9606 GN=CDHR1 PE=1 SV=2	CDHR1	94 kDa	1	3	0	0
792	ADAM DEC1 OS=Homo sapiens OX=9606 GN=ADAMDEC1 PE=1 SV=2	ADAMDEC1	53 kDa	0	2	0	0
803	Fc receptor-like protein 5 OS=Homo sapiens OX=9606 GN=FCRL5 PE=1 SV=3	FCRL5	106 kDa	0	2	0	0
71	Putative keratin-87 protein OS=Homo sapiens OX=9606 GN=KRT87P PE=5 SV=4	KRT87P	29 kDa	51	0	41	63
266	Histone H2B type 1-K OS=Homo sapiens OX=9606 GN=H2BC12 PE=1 SV=3	H2BC12	14 kDa	4	3	2	1
302	Receptor-type tyrosine-protein phosphatase zeta OS=Homo sapiens OX=9606 GN=PTPRZ1 PE=1 SV=4	PTPRZ1	255 kDa	2	2	1	2
280	Histone H4 OS=Homo sapiens OX=9606 GN=H4C1 PE=1 SV=2	H4C1	11 kDa	2	2	1	2
105	Vitamin K-dependent protein C OS=Homo sapiens OX=9606 GN=PROC PE=1 SV=1	PROC	52 kDa	2	8	14	17
245	Immunoglobulin kappa variable 3-15 OS=Homo sapiens OX=9606 GN=IGKV3-15 PE=1 SV=2	IGKV3-15	12 kDa	2	3	3	3
656	Seizure 6-like protein OS=Homo sapiens OX=9606 GN=SEZ6L PE=1 SV=1	SEZ6L	112 kDa	1	1	1	1
204	Immunoglobulin heavy variable 5-51 OS=Homo sapiens OX=9606 GN=IGHV5-51 PE=3 SV=1	IGHV5-51	13 kDa	1	2	2	2
211	Immunoglobulin heavy variable 4-61 OS=Homo sapiens OX=9606 GN=IGHV4-61 PE=3 SV=1	IGHV4-61	13 kDa	1	3	3	3
290	Immunoglobulin kappa variable 2D-29 OS=Homo sapiens OX=9606 GN=IGKV2D-29 PE=3 SV=1	IGKV2D-29	13 kDa	1	2	1	2
324	Immunoglobulin lambda variable 1-47 OS=Homo sapiens OX=9606 GN=IGLV1-47 PE=1 SV=2	IGLV1-47	12 kDa	1	2	1	1
463	Immunoglobulin lambda variable 3-10 OS=Homo sapiens OX=9606 GN=IGLV3-10 PE=3 SV=2	IGLV3-10	12 kDa	1	1	2	1
274	Neuropilin-2 OS=Homo sapiens OX=9606 GN=NRP2 PE=1 SV=3	NRP2	105 kDa	0	2	4	2
323	Immunoglobulin lambda variable 3-19 OS=Homo sapiens OX=9606 GN=IGLV3-19 PE=1 SV=2	IGLV3-19	12 kDa	0	3	1	1
325	Immunoglobulin heavy variable 1-69 OS=Homo sapiens OX=9606 GN=IGHV1-69 PE=1 SV=2	IGHV1-69	13 kDa	0	3	1	2
243	Immunoglobulin lambda variable 1-51 OS=Homo sapiens OX=9606 GN=IGLV1-51 PE=1 SV=2	IGLV1-51	12 kDa	0	6	2	1
190	Immunoglobulin heavy variable 3-74 OS=Homo sapiens OX=9606 GN=IGHV3-74 PE=3 SV=1	IGHV3-74	13 kDa	1	3	3	5
229	Immunoglobulin J chain OS=Homo sapiens OX=9606 GN=JCHAIN PE=1 SV=4	JCHAIN	18 kDa	1	1	3	3
234	Immunoglobulin kappa variable 3-11 OS=Homo sapiens OX=9606 GN=IGKV3-11 PE=1 SV=1	IGKV3-11	13 kDa	1	2	2	3
265	Immunoglobulin heavy variable 3-49 OS=Homo sapiens OX=9606 GN=IGHV3-49 PE=3 SV=1	IGHV3-49	13 kDa	0	1	1	1
244	Thrombospondin-3 OS=Homo sapiens OX=9606 GN=THBS3 PE=1 SV=1	THBS3	104 kDa	0	0	5	5
341	Soluble scavenger receptor cysteine-rich domain-containing protein SSC5D OS=Homo sapiens OX=9606 GN=SSC5D PE=1 SV=3	SSC5D	166 kDa	0	0	11	6
194	Immunoglobulin heavy variable 3-43D OS=Homo sapiens OX=9606 GN=IGHV3-43D PE=3 SV=1	IGHV3-43D	13 kDa	0	0	4	4
221	Plexin domain-containing protein 1 OS=Homo sapiens OX=9606 GN=PLXDC1 PE=1 SV=2	PLXDC1	56 kDa	0	0	2	2
170	Extracellular matrix protein 2 OS=Homo sapiens OX=9606 GN=ECM2 PE=2 SV=1	ECM2	80 kDa	0	0	4	3
318	Blood group Rh(D) polypeptide OS=Homo sapiens OX=9606 GN=RHD PE=1 SV=3	RHD	45 kDa	0	0	1	2
465	Nardilysin OS=Homo sapiens OX=9606 GN=NRDC PE=1 SV=3	NRDC	132 kDa	0	0	0	4
295	Arf-GAP with GTPase, ANK repeat and PH domain-containing protein 9 OS=Homo sapiens OX=9606 GN=AGAP9 PE=3 SV=2	AGAP9	78 kDa	0	0	1	1
334	FYN-binding protein 1 OS=Homo sapiens OX=9606 GN=FYB1 PE=1 SV=2	FYB1	85 kDa	0	0	1	4
354	Aggrecan core protein OS=Homo sapiens OX=9606 GN=ACAN PE=1 SV=3	ACAN	261 kDa	0	0	0	3
503	Caveolae-associated protein 2 OS=Homo sapiens OX=9606 GN=CAVIN2 PE=1 SV=3	CAVIN2	47 kDa	0	0	2	1
223	Adipocyte plasma membrane-associated protein OS=Homo sapiens OX=9606 GN=APMAP PE=1 SV=2	APMAP	46 kDa	0	0	1	2
621	Guanylate cyclase soluble subunit beta-1 OS=Homo sapiens OX=9606 GN=GUCY1B1 PE=1 SV=1	GUCY1B1	71 kDa	0	0	0	1
644	Rho GTPase-activating protein 45 OS=Homo sapiens OX=9606 GN=ARHGAP45 PE=1 SV=2	ARHGAP45	125 kDa	0	0	0	1
754	Erbin OS=Homo sapiens OX=9606 GN=ERBIN PE=1 SV=2	ERBIN	158 kDa	0	0	0	1
785	Immunoglobulin lambda variable 9-49 OS=Homo sapiens OX=9606 GN=IGLV9-49 PE=1 SV=1	IGLV9-49	13 kDa	0	0	0	1
482	Selenoprotein P OS=Homo sapiens OX=9606 GN=SELENOP PE=1 SV=3	SELENOP	43 kDa	0	0	3	2
241	Probable non-functional immunoglobulin kappa variable 2D-24 OS=Homo sapiens OX=9606 GN=IGKV2D-24 PE=5 SV=1	IGKV2D-24	13 kDa	0	0	1	1
209	L-selectin OS=Homo sapiens OX=9606 GN=SELL PE=1 SV=2	SELL	42 kDa	0	0	3	5
281	Immunoglobulin lambda variable 8-61 OS=Homo sapiens OX=9606 GN=IGLV8-61 PE=3 SV=7	IGLV8-61	13 kDa	0	0	2	2
230	Beta-Ala-His dipeptidase OS=Homo sapiens OX=9606 GN=CNDP1 PE=1 SV=4	CNDP1	57 kDa	0	0	3	2
801	Thyroglobulin OS=Homo sapiens OX=9606 GN=TG PE=1 SV=5	TG	305 kDa	0	0	3	0

### OT sample-derived EVs were enriched in proteins related to eye diseases

**Figure 4:**
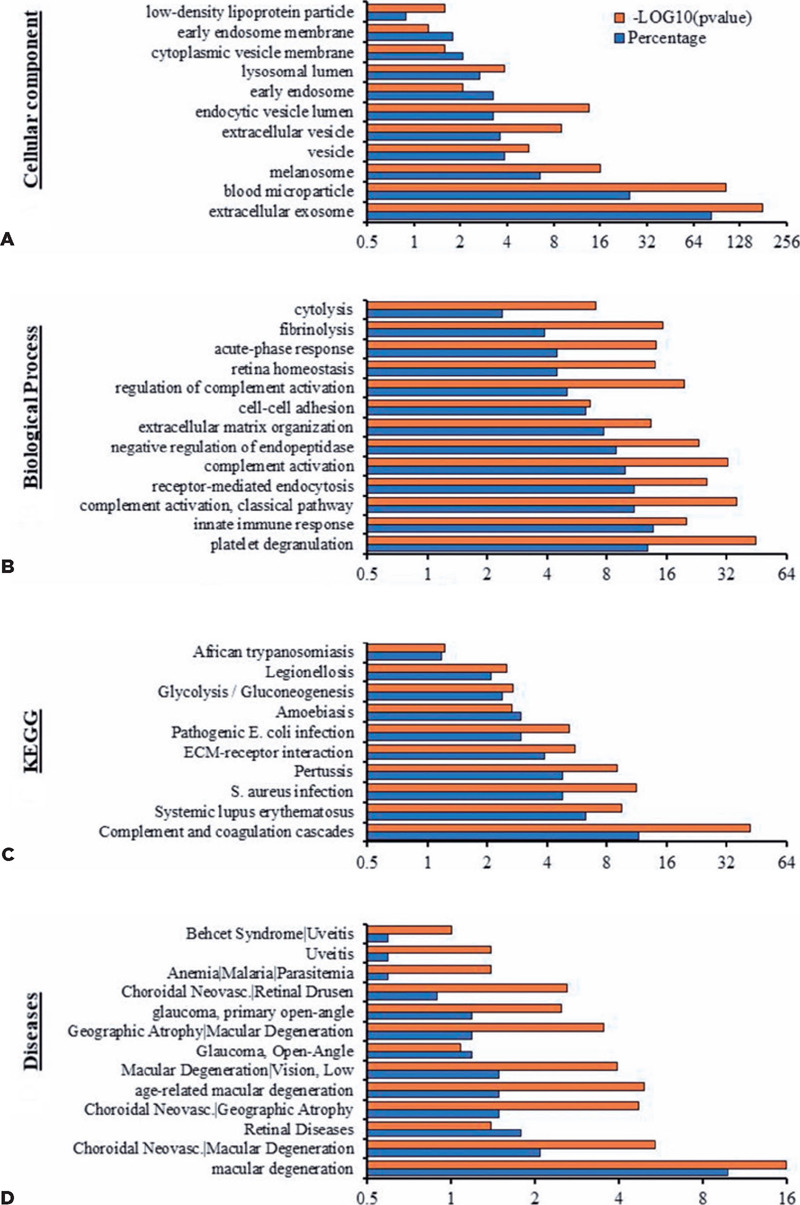
Gene ontology classification of the EV proteins cargo. The most enriched categories in the (A) cellular component, (B) biological process, (C) molecular function, and (D) diseases clustering are depicted. Analyses were performed using the DAVID bioinformatics database. The –LOG10 (p-value) (orange) refers to the *Benjamini-*adjusted p-value. The percentage (blue) refers to the percentage of genes in the DAVID database that are associated with the particular annotation term.

EV proteins from the plasma and AH of patients with OT were clustered to identify their relation to physiological processes (Table 4). Cluster analysis by cellular component demonstrated sets of proteins related to extracellular exosomes, microparticles, vesicles, and endosomes (Figure 4A). Moreover, the analysis highlighted categories consistent with pathways related to complement activation, immune response activation, and retinal homeostasis (Figure 4B).

**Table 4:** Common proteins between the plasma- and aqueous humor-derived EVs in patients with OT.

Accession	Protein Name
P02768	Serum albumin OS=Homo sapiens OX=9606 GN=ALB PE=1 SV=2
P19823	Inter-alpha-trypsin inhibitor heavy chain H2 OS=Homo sapiens OX=9606 GN=ITIH2 PE=1 SV=2
P19827	Inter-alpha-trypsin inhibitor heavy chain H1 OS=Homo sapiens OX=9606 GN=ITIH1 PE=1 SV=3
Q06033	Inter-alpha-trypsin inhibitor heavy chain H3 OS=Homo sapiens OX=9606 GN=ITIH3 PE=1 SV=2
P00734	Prothrombin OS=Homo sapiens OX=9606 GN=F2 PE=1 SV=2
P01024	Complement C3 OS=Homo sapiens OX=9606 GN=C3 PE=1 SV=2
P0C0L5	Complement C4-B OS=Homo sapiens OX=9606 GN=C4B PE=1 SV=2
P0C0L4	Complement C4-A OS=Homo sapiens OX=9606 GN=C4A PE=1 SV=2
P07996	Thrombospondin-1 OS=Homo sapiens OX=9606 GN=THBS1 PE=1 SV=2
Q14520	Hyaluronan-binding protein 2 OS=Homo sapiens OX=9606 GN=HABP2 PE=1 SV=1
P0DOX5	Immunoglobulin gamma-1 heavy chain OS=Homo sapiens OX=9606 PE=1 SV=2
P02787	Serotransferrin OS=Homo sapiens OX=9606 GN=TF PE=1 SV=3
Q9Y490	Talin-1 OS=Homo sapiens OX=9606 GN=TLN1 PE=1 SV=3
P02647	Apolipoprotein A-I OS=Homo sapiens OX=9606 GN=APOA1 PE=1 SV=1
P02675	Fibrinogen beta chain OS=Homo sapiens OX=9606 GN=FGB PE=1 SV=2
P01023	Alpha-2-macroglobulin OS=Homo sapiens OX=9606 GN=A2M PE=1 SV=3
P21333	Filamin-A OS=Homo sapiens OX=9606 GN=FLNA PE=1 SV=4
P02671	Fibrinogen alpha chain OS=Homo sapiens OX=9606 GN=FGA PE=1 SV=2
P01834	Immunoglobulin kappa constant OS=Homo sapiens OX=9606 GN=IGKC PE=1 SV=2
P04264	Keratin, type II cytoskeletal 1 OS=Homo sapiens OX=9606 GN=KRT1 PE=1 SV=6
P02679	Fibrinogen gamma chain OS=Homo sapiens OX=9606 GN=FGG PE=1 SV=3
P12259	Coagulation factor V OS=Homo sapiens OX=9606 GN=F5 PE=1 SV=4
P13645	Keratin, type I cytoskeletal 10 OS=Homo sapiens OX=9606 GN=KRT10 PE=1 SV=6
P02760	Protein AMBP OS=Homo sapiens OX=9606 GN=AMBP PE=1 SV=1
P01860	Immunoglobulin heavy constant gamma 3 OS=Homo sapiens OX=9606 GN=IGHG3 PE=1 SV=2
P35527	Keratin, type I cytoskeletal 9 OS=Homo sapiens OX=9606 GN=KRT9 PE=1 SV=3
P01859	Immunoglobulin heavy constant gamma 2 OS=Homo sapiens OX=9606 GN=IGHG2 PE=1 SV=2
P68871	Hemoglobin subunit beta OS=Homo sapiens OX=9606 GN=HBB PE=1 SV=2
P01009	Alpha-1-antitrypsin OS=Homo sapiens OX=9606 GN=SERPINA1 PE=1 SV=3
P49747	Cartilage oligomeric matrix protein OS=Homo sapiens OX=9606 GN=COMP PE=1 SV=2
P0DOX7	Immunoglobulin kappa light chain OS=Homo sapiens OX=9606 PE=1 SV=1
P00450	Ceruloplasmin OS=Homo sapiens OX=9606 GN=CP PE=1 SV=1
P10909	Clusterin OS=Homo sapiens OX=9606 GN=CLU PE=1 SV=1
P01861	Immunoglobulin heavy constant gamma 4 OS=Homo sapiens OX=9606 GN=IGHG4 PE=1 SV=1
P60709	Actin, cytoplasmic 1 OS=Homo sapiens OX=9606 GN=ACTB PE=1 SV=1
P02730	Band 3 anion transport protein OS=Homo sapiens OX=9606 GN=SLC4A1 PE=1 SV=3
P04004	Vitronectin OS=Homo sapiens OX=9606 GN=VTN PE=1 SV=1
P35908	Keratin, type II cytoskeletal 2 epidermal OS=Homo sapiens OX=9606 GN=KRT2 PE=1 SV=2
P00740	Coagulation factor IX OS=Homo sapiens OX=9606 GN=F9 PE=1 SV=2
P01871	Immunoglobulin heavy constant mu OS=Homo sapiens OX=9606 GN=IGHM PE=1 SV=4
P69905	Hemoglobin subunit alpha OS=Homo sapiens OX=9606 GN=HBA1 PE=1 SV=2
P08514	Integrin alpha-IIb OS=Homo sapiens OX=9606 GN=ITGA2B PE=1 SV=3
P10745	Retinol-binding protein 3 OS=Homo sapiens OX=9606 GN=RBP3 PE=1 SV=2
P00738	Haptoglobin OS=Homo sapiens OX=9606 GN=HP PE=1 SV=1
P51884	Lumican OS=Homo sapiens OX=9606 GN=LUM PE=1 SV=2
P01876	Immunoglobulin heavy constant alpha 1 OS=Homo sapiens OX=9606 GN=IGHA1 PE=1 SV=2
P02649	Apolipoprotein E OS=Homo sapiens OX=9606 GN=APOE PE=1 SV=1
P06727	Apolipoprotein A-IV OS=Homo sapiens OX=9606 GN=APOA4 PE=1 SV=3
P00742	Coagulation factor X OS=Homo sapiens OX=9606 GN=F10 PE=1 SV=2
P02774	Vitamin D-binding protein OS=Homo sapiens OX=9606 GN=GC PE=1 SV=2
P02042	Hemoglobin subunit delta OS=Homo sapiens OX=9606 GN=HBD PE=1 SV=2
P02766	Transthyretin OS=Homo sapiens OX=9606 GN=TTR PE=1 SV=1
P0DOY2	Immunoglobulin lambda constant 2 OS=Homo sapiens OX=9606 GN=IGLC2 PE=1 SV=1
P02751	Fibronectin OS=Homo sapiens OX=9606 GN=FN1 PE=1 SV=5
P06396	Gelsolin OS=Homo sapiens OX=9606 GN=GSN PE=1 SV=1
Q08380	Galectin-3-binding protein OS=Homo sapiens OX=9606 GN=LGALS3BP PE=1 SV=1
P27169	Serum paraoxonase/arylesterase 1 OS=Homo sapiens OX=9606 GN=PON1 PE=1 SV=3
P05155	Plasma protease C1 inhibitor OS=Homo sapiens OX=9606 GN=SERPING1 PE=1 SV=2
Q86UX7	Fermitin family homolog 3 OS=Homo sapiens OX=9606 GN=FERMT3 PE=1 SV=1
P02790	Hemopexin OS=Homo sapiens OX=9606 GN=HPX PE=1 SV=2
P09871	Complement C1s subcomponent OS=Homo sapiens OX=9606 GN=C1S PE=1 SV=1
P01042	Kininogen-1 OS=Homo sapiens OX=9606 GN=KNG1 PE=1 SV=2
P00736	Complement C1r subcomponent OS=Homo sapiens OX=9606 GN=C1R PE=1 SV=2
P14625	Endoplasmin OS=Homo sapiens OX=9606 GN=HSP90B1 PE=1 SV=1
P07900	Heat shock protein HSP 90-alpha OS=Homo sapiens OX=9606 GN=HSP90AA1 PE=1 SV=5
P13647	Keratin, type II cytoskeletal 5 OS=Homo sapiens OX=9606 GN=KRT5 PE=1 SV=3
P04003	C4b-binding protein alpha chain OS=Homo sapiens OX=9606 GN=C4BPA PE=1 SV=2
P14543	Nidogen-1 OS=Homo sapiens OX=9606 GN=NID1 PE=1 SV=3
Q14624	Inter-alpha-trypsin inhibitor heavy chain H4 OS=Homo sapiens OX=9606 GN=ITIH4 PE=1 SV=4
P02538	Keratin, type II cytoskeletal 6A OS=Homo sapiens OX=9606 GN=KRT6A PE=1 SV=3
P00739	Haptoglobin-related protein OS=Homo sapiens OX=9606 GN=HPR PE=2 SV=2
P04259	Keratin, type II cytoskeletal 6B OS=Homo sapiens OX=9606 GN=KRT6B PE=1 SV=5
P22891	Vitamin K-dependent protein Z OS=Homo sapiens OX=9606 GN=PROZ PE=1 SV=2
P26038	Moesin OS=Homo sapiens OX=9606 GN=MSN PE=1 SV=3
P08697	Alpha-2-antiplasmin OS=Homo sapiens OX=9606 GN=SERPINF2 PE=1 SV=3
P01008	Antithrombin-III OS=Homo sapiens OX=9606 GN=SERPINC1 PE=1 SV=1
P05106	Integrin beta-3 OS=Homo sapiens OX=9606 GN=ITGB3 PE=1 SV=2
P02533	Keratin, type I cytoskeletal 14 OS=Homo sapiens OX=9606 GN=KRT14 PE=1 SV=4
P01019	Angiotensinogen OS=Homo sapiens OX=9606 GN=AGT PE=1 SV=1
P0DOX8	Immunoglobulin lambda-1 light chain OS=Homo sapiens OX=9606 PE=1 SV=1
Q14515	SPARC-like protein 1 OS=Homo sapiens OX=9606 GN=SPARCL1 PE=1 SV=2
P08603	Complement factor H OS=Homo sapiens OX=9606 GN=CFH PE=1 SV=4
P01011	Alpha-1-antichymotrypsin OS=Homo sapiens OX=9606 GN=SERPINA3 PE=1 SV=2
P07359	Platelet glycoprotein Ib alpha chain OS=Homo sapiens OX=9606 GN=GP1BA PE=1 SV=2
P0DOX2	Immunoglobulin alpha-2 heavy chain OS=Homo sapiens OX=9606 PE=1 SV=2
P07225	Vitamin K-dependent protein S OS=Homo sapiens OX=9606 GN=PROS1 PE=1 SV=1
P08779	Keratin, type I cytoskeletal 16 OS=Homo sapiens OX=9606 GN=KRT16 PE=1 SV=4
P61224	Ras-related protein Rap-1b OS=Homo sapiens OX=9606 GN=RAP1B PE=1 SV=1
P27797	Calreticulin OS=Homo sapiens OX=9606 GN=CALR PE=1 SV=1
P00747	Plasminogen OS=Homo sapiens OX=9606 GN=PLG PE=1 SV=2
P55072	Transitional endoplasmic reticulum ATPase OS=Homo sapiens OX=9606 GN=VCP PE=1 SV=4
P05090	Apolipoprotein D OS=Homo sapiens OX=9606 GN=APOD PE=1 SV=1
P63104	14-3-3 protein zeta/delta OS=Homo sapiens OX=9606 GN=YWHAZ PE=1 SV=1
P00751	Complement factor B OS=Homo sapiens OX=9606 GN=CFB PE=1 SV=2
P68363	Tubulin alpha-1B chain OS=Homo sapiens OX=9606 GN=TUBA1B PE=1 SV=1
P02765	Alpha-2-HS-glycoprotein OS=Homo sapiens OX=9606 GN=AHSG PE=1 SV=2
P04196	Histidine-rich glycoprotein OS=Homo sapiens OX=9606 GN=HRG PE=1 SV=1
P04406	Glyceraldehyde-3-phosphate dehydrogenase OS=Homo sapiens OX=9606 GN=GAPDH PE=1 SV=3
P08238	Heat shock protein HSP 90-beta OS=Homo sapiens OX=9606 GN=HSP90AB1 PE=1 SV=4
P02652	Apolipoprotein A-II OS=Homo sapiens OX=9606 GN=APOA2 PE=1 SV=1
P04217	Alpha-1B-glycoprotein OS=Homo sapiens OX=9606 GN=A1BG PE=1 SV=4
P68366	Tubulin alpha-4A chain OS=Homo sapiens OX=9606 GN=TUBA4A PE=1 SV=1
P05546	Heparin cofactor 2 OS=Homo sapiens OX=9606 GN=SERPIND1 PE=1 SV=3
Q71U36	Tubulin alpha-1A chain OS=Homo sapiens OX=9606 GN=TUBA1A PE=1 SV=1
P13591	Neural cell adhesion molecule 1 OS=Homo sapiens OX=9606 GN=NCAM1 PE=1 SV=3
P02748	Complement component C9 OS=Homo sapiens OX=9606 GN=C9 PE=1 SV=2
P12814	Alpha-actinin-1 OS=Homo sapiens OX=9606 GN=ACTN1 PE=1 SV=2
P02749	Beta-2-glycoprotein 1 OS=Homo sapiens OX=9606 GN=APOH PE=1 SV=3
P14618	Pyruvate kinase PKM OS=Homo sapiens OX=9606 GN=PKM PE=1 SV=4
P06733	Alpha-enolase OS=Homo sapiens OX=9606 GN=ENO1 PE=1 SV=2
Q04695	Keratin, type I cytoskeletal 17 OS=Homo sapiens OX=9606 GN=KRT17 PE=1 SV=2
P05067	Amyloid-beta precursor protein OS=Homo sapiens OX=9606 GN=APP PE=1 SV=3
P32119	Peroxiredoxin-2 OS=Homo sapiens OX=9606 GN=PRDX2 PE=1 SV=5
P07437	Tubulin beta chain OS=Homo sapiens OX=9606 GN=TUBB PE=1 SV=2
Q7Z794	Keratin, type II cytoskeletal 1b OS=Homo sapiens OX=9606 GN=KRT77 PE=2 SV=3
Q9UBP4	Dickkopf-related protein 3 OS=Homo sapiens OX=9606 GN=DKK3 PE=1 SV=2
Q9UK55	Protein Z-dependent protease inhibitor OS=Homo sapiens OX=9606 GN=SERPINA10 PE=1 SV=1
P02763	Alpha-1-acid glycoprotein 1 OS=Homo sapiens OX=9606 GN=ORM1 PE=1 SV=1
P36955	Pigment epithelium-derived factor OS=Homo sapiens OX=9606 GN=SERPINF1 PE=1 SV=4
P04070	Vitamin K-dependent protein C OS=Homo sapiens OX=9606 GN=PROC PE=1 SV=1
O94985	Calsyntenin-1 OS=Homo sapiens OX=9606 GN=CLSTN1 PE=1 SV=1
P01031	Complement C5 OS=Homo sapiens OX=9606 GN=C5 PE=1 SV=4
O95678	Keratin, type II cytoskeletal 75 OS=Homo sapiens OX=9606 GN=KRT75 PE=1 SV=2
P35579	Myosin-9 OS=Homo sapiens OX=9606 GN=MYH9 PE=1 SV=4
P07737	Profilin-1 OS=Homo sapiens OX=9606 GN=PFN1 PE=1 SV=2
P62258	14-3-3 protein epsilon OS=Homo sapiens OX=9606 GN=YWHAE PE=1 SV=1
P68371	Tubulin beta-4B chain OS=Homo sapiens OX=9606 GN=TUBB4B PE=1 SV=1
P08670	Vimentin OS=Homo sapiens OX=9606 GN=VIM PE=1 SV=4
P43652	Afamin OS=Homo sapiens OX=9606 GN=AFM PE=1 SV=1
P05154	Plasma serine protease inhibitor OS=Homo sapiens OX=9606 GN=SERPINA5 PE=1 SV=3
Q9H4B7	Tubulin beta-1 chain OS=Homo sapiens OX=9606 GN=TUBB1 PE=1 SV=1
Q15323	Keratin, type I cuticular Ha1 OS=Homo sapiens OX=9606 GN=KRT31 PE=1 SV=3
O43790	Keratin, type II cuticular Hb6 OS=Homo sapiens OX=9606 GN=KRT86 PE=1 SV=1
P23528	Cofilin-1 OS=Homo sapiens OX=9606 GN=CFL1 PE=1 SV=3
P09486	SPARC OS=Homo sapiens OX=9606 GN=SPARC PE=1 SV=1
P04040	Catalase OS=Homo sapiens OX=9606 GN=CAT PE=1 SV=3
Q12805	EGF-containing fibulin-like extracellular matrix protein 1 OS=Homo sapiens OX=9606 GN=EFEMP1 PE=1 SV=2
P18428	Lipopolysaccharide-binding protein OS=Homo sapiens OX=9606 GN=LBP PE=1 SV=3
P02656	Apolipoprotein C-III OS=Homo sapiens OX=9606 GN=APOC3 PE=1 SV=1
P31946	14-3-3 protein beta/alpha OS=Homo sapiens OX=9606 GN=YWHAB PE=1 SV=3
Q06481	Amyloid-like protein 2 OS=Homo sapiens OX=9606 GN=APLP2 PE=1 SV=2
P07339	Cathepsin D OS=Homo sapiens OX=9606 GN=CTSD PE=1 SV=1
P78386	Keratin, type II cuticular Hb5 OS=Homo sapiens OX=9606 GN=KRT85 PE=1 SV=1
P06702	Protein S100-A9 OS=Homo sapiens OX=9606 GN=S100A9 PE=1 SV=1
P11142	Heat shock cognate 71 kDa protein OS=Homo sapiens OX=9606 GN=HSPA8 PE=1 SV=1
P02753	Retinol-binding protein 4 OS=Homo sapiens OX=9606 GN=RBP4 PE=1 SV=3
Q14525	Keratin, type I cuticular Ha3-II OS=Homo sapiens OX=9606 GN=KRT33B PE=1 SV=3
P67936	Tropomyosin alpha-4 chain OS=Homo sapiens OX=9606 GN=TPM4 PE=1 SV=3
P61981	14-3-3 protein gamma OS=Homo sapiens OX=9606 GN=YWHAG PE=1 SV=2
P04075	Fructose-bisphosphate aldolase A OS=Homo sapiens OX=9606 GN=ALDOA PE=1 SV=2
P25311	Zinc-alpha-2-glycoprotein OS=Homo sapiens OX=9606 GN=AZGP1 PE=1 SV=2
P07237	Protein disulfide-isomerase OS=Homo sapiens OX=9606 GN=P4HB PE=1 SV=3
P02788	Lactotransferrin OS=Homo sapiens OX=9606 GN=LTF PE=1 SV=6
P23142	Fibulin-1 OS=Homo sapiens OX=9606 GN=FBLN1 PE=1 SV=4
Q01518	Adenylyl cyclase-associated protein 1 OS=Homo sapiens OX=9606 GN=CAP1 PE=1 SV=5
P19652	Alpha-1-acid glycoprotein 2 OS=Homo sapiens OX=9606 GN=ORM2 PE=1 SV=2
Q04917	14-3-3 protein eta OS=Homo sapiens OX=9606 GN=YWHAH PE=1 SV=4
P27348	14-3-3 protein theta OS=Homo sapiens OX=9606 GN=YWHAQ PE=1 SV=1
P04439	HLA class I histocompatibility antigen, A alpha chain OS=Homo sapiens OX=9606 GN=HLA-A PE=1 SV=2
O14791	Apolipoprotein L1 OS=Homo sapiens OX=9606 GN=APOL1 PE=1 SV=5
P10321	HLA class I histocompatibility antigen, C alpha chain OS=Homo sapiens OX=9606 GN=HLA-C PE=1 SV=3
P05109	Protein S100-A8 OS=Homo sapiens OX=9606 GN=S100A8 PE=1 SV=1
P00338	L-lactate dehydrogenase A chain OS=Homo sapiens OX=9606 GN=LDHA PE=1 SV=2
P01889	HLA class I histocompatibility antigen, B alpha chain OS=Homo sapiens OX=9606 GN=HLA-B PE=1 SV=3
P07195	L-lactate dehydrogenase B chain OS=Homo sapiens OX=9606 GN=LDHB PE=1 SV=2
P02743	Serum amyloid P-component OS=Homo sapiens OX=9606 GN=APCS PE=1 SV=2
O14786	Neuropilin-1 OS=Homo sapiens OX=9606 GN=NRP1 PE=1 SV=3
P98160	Basement membrane-specific heparan sulfate proteoglycan core protein OS=Homo sapiens OX=9606 GN=HSPG2 PE=1 SV=4
P04899	Guanine nucleotide-binding protein G(i) subunit alpha-2 OS=Homo sapiens OX=9606 GN=GNAI2 PE=1 SV=3
O76011	Keratin, type I cuticular Ha4 OS=Homo sapiens OX=9606 GN=KRT34 PE=1 SV=2
P01780	Immunoglobulin heavy variable 3-7 OS=Homo sapiens OX=9606 GN=IGHV3-7 PE=1 SV=2
P08571	Monocyte differentiation antigen CD14 OS=Homo sapiens OX=9606 GN=CD14 PE=1 SV=2
Q9P2E9	Ribosome-binding protein 1 OS=Homo sapiens OX=9606 GN=RRBP1 PE=1 SV=5
P39060	Collagen alpha-1(XVIII) chain OS=Homo sapiens OX=9606 GN=COL18A1 PE=1 SV=5
Q15582	Transforming growth factor-beta-induced protein ig-h3 OS=Homo sapiens OX=9606 GN=TGFBI PE=1 SV=1
P07358	Complement component C8 beta chain OS=Homo sapiens OX=9606 GN=C8B PE=1 SV=3
P24821	Tenascin OS=Homo sapiens OX=9606 GN=TNC PE=1 SV=3
Q86YZ3	Hornerin OS=Homo sapiens OX=9606 GN=HRNR PE=1 SV=2
A0A0B4J1X5	Immunoglobulin heavy variable 3-74 OS=Homo sapiens OX=9606 GN=IGHV3-74 PE=3 SV=1
P11021	Endoplasmic reticulum chaperone BiP OS=Homo sapiens OX=9606 GN=HSPA5 PE=1 SV=2
P15924	Desmoplakin OS=Homo sapiens OX=9606 GN=DSP PE=1 SV=3
P80108	Phosphatidylinositol-glycan-specific phospholipase D OS=Homo sapiens OX=9606 GN=GPLD1 PE=1 SV=3
Q92688	Acidic leucine-rich nuclear phosphoprotein 32 family member B OS=Homo sapiens OX=9606 GN=ANP32B PE=1 SV=1
P01619	Immunoglobulin kappa variable 3-20 OS=Homo sapiens OX=9606 GN=IGKV3-20 PE=1 SV=2
P16070	CD44 antigen OS=Homo sapiens OX=9606 GN=CD44 PE=1 SV=3
P06753	Tropomyosin alpha-3 chain OS=Homo sapiens OX=9606 GN=TPM3 PE=1 SV=2
Q02413	Desmoglein-1 OS=Homo sapiens OX=9606 GN=DSG1 PE=1 SV=2
Q9H0U4	Ras-related protein Rab-1B OS=Homo sapiens OX=9606 GN=RAB1B PE=1 SV=1
P55058	Phospholipid transfer protein OS=Homo sapiens OX=9606 GN=PLTP PE=1 SV=1
P22352	Glutathione peroxidase 3 OS=Homo sapiens OX=9606 GN=GPX3 PE=1 SV=2
P60174	Triosephosphate isomerase OS=Homo sapiens OX=9606 GN=TPI1 PE=1 SV=3
Q96PD5	N-acetylmuramoyl-L-alanine amidase OS=Homo sapiens OX=9606 GN=PGLYRP2 PE=1 SV=1
P13667	Protein disulfide-isomerase A4 OS=Homo sapiens OX=9606 GN=PDIA4 PE=1 SV=2
Q8N1N4	Keratin, type II cytoskeletal 78 OS=Homo sapiens OX=9606 GN=KRT78 PE=1 SV=2
P01893	Putative HLA class I histocompatibility antigen, alpha chain H OS=Homo sapiens OX=9606 GN=HLA-H PE=5 SV=3
Q07507	Dermatopontin OS=Homo sapiens OX=9606 GN=DPT PE=1 SV=2
P02654	Apolipoprotein C-I OS=Homo sapiens OX=9606 GN=APOC1 PE=1 SV=1
P06312	Immunoglobulin kappa variable 4-1 OS=Homo sapiens OX=9606 GN=IGKV4-1 PE=1 SV=1
P29622	Kallistatin OS=Homo sapiens OX=9606 GN=SERPINA4 PE=1 SV=3
Q99983	Osteomodulin OS=Homo sapiens OX=9606 GN=OMD PE=1 SV=1
P10451	Osteopontin OS=Homo sapiens OX=9606 GN=SPP1 PE=1 SV=1
P62979	Ubiquitin-40S ribosomal protein S27a OS=Homo sapiens OX=9606 GN=RPS27A PE=1 SV=2
Q03591	Complement factor H-related protein 1 OS=Homo sapiens OX=9606 GN=CFHR1 PE=1 SV=2
P14314	Glucosidase 2 subunit beta OS=Homo sapiens OX=9606 GN=PRKCSH PE=1 SV=2
O00468	Agrin OS=Homo sapiens OX=9606 GN=AGRN PE=1 SV=6
Q8N8A2	Serine/threonine-protein phosphatase 6 regulatory ankyrin repeat subunit B OS=Homo sapiens OX=9606 GN=ANKRD44 PE=1 SV=3
Q9Y240	C-type lectin domain family 11 member A OS=Homo sapiens OX=9606 GN=CLEC11A PE=1 SV=1
P30041	Peroxiredoxin-6 OS=Homo sapiens OX=9606 GN=PRDX6 PE=1 SV=3
P27824	Calnexin OS=Homo sapiens OX=9606 GN=CANX PE=1 SV=2
P10643	Complement component C7 OS=Homo sapiens OX=9606 GN=C7 PE=1 SV=2
P14151	L-selectin OS=Homo sapiens OX=9606 GN=SELL PE=1 SV=2
P00558	Phosphoglycerate kinase 1 OS=Homo sapiens OX=9606 GN=PGK1 PE=1 SV=3
P08185	Corticosteroid-binding globulin OS=Homo sapiens OX=9606 GN=SERPINA6 PE=1 SV=1
O00299	Chloride intracellular channel protein 1 OS=Homo sapiens OX=9606 GN=CLIC1 PE=1 SV=4
P55209	Nucleosome assembly protein 1-like 1 OS=Homo sapiens OX=9606 GN=NAP1L1 PE=1 SV=1
P61204	ADP-ribosylation factor 3 OS=Homo sapiens OX=9606 GN=ARF3 PE=1 SV=2
P39687	Acidic leucine-rich nuclear phosphoprotein 32 family member A OS=Homo sapiens OX=9606 GN=ANP32A PE=1 SV=1
A0A0C4DH41	Immunoglobulin heavy variable 4-61 OS=Homo sapiens OX=9606 GN=IGHV4-61 PE=3 SV=1
P15153	Ras-related C3 botulinum toxin substrate 2 OS=Homo sapiens OX=9606 GN=RAC2 PE=1 SV=1
P0DJI9	Serum amyloid A-2 protein OS=Homo sapiens OX=9606 GN=SAA2 PE=1 SV=1
Q06830	Peroxiredoxin-1 OS=Homo sapiens OX=9606 GN=PRDX1 PE=1 SV=1
P13671	Complement component C6 OS=Homo sapiens OX=9606 GN=C6 PE=1 SV=3
P07355	Annexin A2 OS=Homo sapiens OX=9606 GN=ANXA2 PE=1 SV=2
P09429	High mobility group protein B1 OS=Homo sapiens OX=9606 GN=HMGB1 PE=1 SV=3
P01624	Immunoglobulin kappa variable 3-15 OS=Homo sapiens OX=9606 GN=IGKV3-15 PE=1 SV=2
O60462	Neuropilin-2 OS=Homo sapiens OX=9606 GN=NRP2 PE=1 SV=3
P22792	Carboxypeptidase N subunit 2 OS=Homo sapiens OX=9606 GN=CPN2 PE=1 SV=3
Q13790	Apolipoprotein F OS=Homo sapiens OX=9606 GN=APOF PE=1 SV=2
P01591	Immunoglobulin J chain OS=Homo sapiens OX=9606 GN=JCHAIN PE=1 SV=4
A0A075B6S2	Immunoglobulin kappa variable 2D-29 OS=Homo sapiens OX=9606 GN=IGKV2D-29 PE=3 SV=1
P0DP23	Calmodulin-1 OS=Homo sapiens OX=9606 GN=CALM1 PE=1 SV=1
P35858	Insulin-like growth factor-binding protein complex acid labile subunit OS=Homo sapiens OX=9606 GN=IGFALS PE=1 SV=1
Q00610	Clathrin heavy chain 1 OS=Homo sapiens OX=9606 GN=CLTC PE=1 SV=5
P62873	Guanine nucleotide-binding protein G(I)/G(S)/G(T) subunit beta-1 OS=Homo sapiens OX=9606 GN=GNB1 PE=1 SV=3
P11597	Cholesteryl ester transfer protein OS=Homo sapiens OX=9606 GN=CETP PE=1 SV=2
P31146	Coronin-1A OS=Homo sapiens OX=9606 GN=CORO1A PE=1 SV=4
Q14974	Importin subunit beta-1 OS=Homo sapiens OX=9606 GN=KPNB1 PE=1 SV=2
P05156	Complement factor I OS=Homo sapiens OX=9606 GN=CFI PE=1 SV=2
P04433	Immunoglobulin kappa variable 3-11 OS=Homo sapiens OX=9606 GN=IGKV3-11 PE=1 SV=1
P68104	Elongation factor 1-alpha 1 OS=Homo sapiens OX=9606 GN=EEF1A1 PE=1 SV=1
P80748	Immunoglobulin lambda variable 3-21 OS=Homo sapiens OX=9606 GN=IGLV3-21 PE=1 SV=2
Q14764	Major vault protein OS=Homo sapiens OX=9606 GN=MVP PE=1 SV=4
Q16610	Extracellular matrix protein 1 OS=Homo sapiens OX=9606 GN=ECM1 PE=1 SV=2
P61769	Beta-2-microglobulin OS=Homo sapiens OX=9606 GN=B2M PE=1 SV=1
P0DJI8	Serum amyloid A-1 protein OS=Homo sapiens OX=9606 GN=SAA1 PE=1 SV=1
P52907	F-actin-capping protein subunit alpha-1 OS=Homo sapiens OX=9606 GN=CAPZA1 PE=1 SV=3
Q9NQ79	Cartilage acidic protein 1 OS=Homo sapiens OX=9606 GN=CRTAC1 PE=1 SV=2
Q14697	Neutral alpha-glucosidase AB OS=Homo sapiens OX=9606 GN=GANAB PE=1 SV=3
P14923	Junction plakoglobin OS=Homo sapiens OX=9606 GN=JUP PE=1 SV=3
P02747	Complement C1q subcomponent subunit C OS=Homo sapiens OX=9606 GN=C1QC PE=1 SV=3
P35542	Serum amyloid A-4 protein OS=Homo sapiens OX=9606 GN=SAA4 PE=1 SV=2
P02746	Complement C1q subcomponent subunit B OS=Homo sapiens OX=9606 GN=C1QB PE=1 SV=3
P61626	Lysozyme C OS=Homo sapiens OX=9606 GN=LYZ PE=1 SV=1
P02750	Leucine-rich alpha-2-glycoprotein OS=Homo sapiens OX=9606 GN=LRG1 PE=1 SV=2
P01701	Immunoglobulin lambda variable 1-51 OS=Homo sapiens OX=9606 GN=IGLV1-51 PE=1 SV=2
O75955	Flotillin-1 OS=Homo sapiens OX=9606 GN=FLOT1 PE=1 SV=3
A0A0C4DH38	Immunoglobulin heavy variable 5-51 OS=Homo sapiens OX=9606 GN=IGHV5-51 PE=3 SV=1
P00748	Coagulation factor XII OS=Homo sapiens OX=9606 GN=F12 PE=1 SV=3
O95445	Apolipoprotein M OS=Homo sapiens OX=9606 GN=APOM PE=1 SV=2
P13796	Plastin-2 OS=Homo sapiens OX=9606 GN=LCP1 PE=1 SV=6
P05388	60S acidic ribosomal protein P0 OS=Homo sapiens OX=9606 GN=RPLP0 PE=1 SV=1
P04745	Alpha-amylase 1 OS=Homo sapiens OX=9606 GN=AMY1A PE=1 SV=2
P10645	Chromogranin-A OS=Homo sapiens OX=9606 GN=CHGA PE=1 SV=7
A0A075B6R9	Probable non-functional immunoglobulin kappa variable 2D-24 OS=Homo sapiens OX=9606 GN=IGKV2D-24 PE=5 SV=1
P19338	Nucleolin OS=Homo sapiens OX=9606 GN=NCL PE=1 SV=3
P98164	Low-density lipoprotein receptor-related protein 2 OS=Homo sapiens OX=9606 GN=LRP2 PE=1 SV=3
P08311	Cathepsin G OS=Homo sapiens OX=9606 GN=CTSG PE=1 SV=2
P07357	Complement component C8 alpha chain OS=Homo sapiens OX=9606 GN=C8A PE=1 SV=2
P07360	Complement component C8 gamma chain OS=Homo sapiens OX=9606 GN=C8G PE=1 SV=3
A0A075B6K4	Immunoglobulin lambda variable 3-10 OS=Homo sapiens OX=9606 GN=IGLV3-10 PE=3 SV=2
Q01105	Protein SET OS=Homo sapiens OX=9606 GN=SET PE=1 SV=3
P13611	Versican core protein OS=Homo sapiens OX=9606 GN=VCAN PE=1 SV=3
P09211	Glutathione S-transferase P OS=Homo sapiens OX=9606 GN=GSTP1 PE=1 SV=2
P47756	F-actin-capping protein subunit beta OS=Homo sapiens OX=9606 GN=CAPZB PE=1 SV=4
P59665	Neutrophil defensin 1 OS=Homo sapiens OX=9606 GN=DEFA1 PE=1 SV=1
Q5D862	Filaggrin-2 OS=Homo sapiens OX=9606 GN=FLG2 PE=1 SV=1
Q8NBJ4	Golgi membrane protein 1 OS=Homo sapiens OX=9606 GN=GOLM1 PE=1 SV=1
P04792	Heat shock protein beta-1 OS=Homo sapiens OX=9606 GN=HSPB1 PE=1 SV=2
P05452	Tetranectin OS=Homo sapiens OX=9606 GN=CLEC3B PE=1 SV=3
P23471	Receptor-type tyrosine-protein phosphatase zeta OS=Homo sapiens OX=9606 GN=PTPRZ1 PE=1 SV=4
A0A075B6I0	Immunoglobulin lambda variable 8-61 OS=Homo sapiens OX=9606 GN=IGLV8-61 PE=3 SV=7
P06681	Complement C2 OS=Homo sapiens OX=9606 GN=C2 PE=1 SV=2
Q14393	Growth arrest-specific protein 6 OS=Homo sapiens OX=9606 GN=GAS6 PE=1 SV=3
P46940	Ras GTPase-activating-like protein IQGAP1 OS=Homo sapiens OX=9606 GN=IQGAP1 PE=1 SV=1
O00533	Neural cell adhesion molecule L1-like protein OS=Homo sapiens OX=9606 GN=CHL1 PE=1 SV=4
P43251	Biotinidase OS=Homo sapiens OX=9606 GN=BTD PE=1 SV=2
Q96KN2	Beta-Ala-His dipeptidase OS=Homo sapiens OX=9606 GN=CNDP1 PE=1 SV=4
Q7Z7G0	Target of Nesh-SH3 OS=Homo sapiens OX=9606 GN=ABI3BP PE=1 SV=1
P26583	High mobility group protein B2 OS=Homo sapiens OX=9606 GN=HMGB2 PE=1 SV=2
P29401	Transketolase OS=Homo sapiens OX=9606 GN=TKT PE=1 SV=3
A0A0A0MS15	Immunoglobulin heavy variable 3-49 OS=Homo sapiens OX=9606 GN=IGHV3-49 PE=3 SV=1
Q08554	Desmocollin-1 OS=Homo sapiens OX=9606 GN=DSC1 PE=1 SV=2
P01714	Immunoglobulin lambda variable 3-19 OS=Homo sapiens OX=9606 GN=IGLV3-19 PE=1 SV=2
P26641	Elongation factor 1-gamma OS=Homo sapiens OX=9606 GN=EEF1G PE=1 SV=3
O00391	Sulfhydryl oxidase 1 OS=Homo sapiens OX=9606 GN=QSOX1 PE=1 SV=3
Q86VP6	Cullin-associated NEDD8-dissociated protein 1 OS=Homo sapiens OX=9606 GN=CAND1 PE=1 SV=2
P10599	Thioredoxin OS=Homo sapiens OX=9606 GN=TXN PE=1 SV=3
A0A0B4J1Y9	Immunoglobulin heavy variable 3-72 OS=Homo sapiens OX=9606 GN=IGHV3-72 PE=3 SV=1
P01700	Immunoglobulin lambda variable 1-47 OS=Homo sapiens OX=9606 GN=IGLV1-47 PE=1 SV=2
P01742	Immunoglobulin heavy variable 1-69 OS=Homo sapiens OX=9606 GN=IGHV1-69 PE=1 SV=2
O60814	Histone H2B type 1-K OS=Homo sapiens OX=9606 GN=H2BC12 PE=1 SV=3
P50502	Hsc70-interacting protein OS=Homo sapiens OX=9606 GN=ST13 PE=1 SV=2
P60660	Myosin light polypeptide 6 OS=Homo sapiens OX=9606 GN=MYL6 PE=1 SV=2
P62805	Histone H4 OS=Homo sapiens OX=9606 GN=H4C1 PE=1 SV=2
P19971	Thymidine phosphorylase OS=Homo sapiens OX=9606 GN=TYMP PE=1 SV=2
P06748	Nucleophosmin OS=Homo sapiens OX=9606 GN=NPM1 PE=1 SV=2
Q00839	Heterogeneous nuclear ribonucleoprotein U OS=Homo sapiens OX=9606 GN=HNRNPU PE=1 SV=6
P60900	Proteasome subunit alpha type-6 OS=Homo sapiens OX=9606 GN=PSMA6 PE=1 SV=1
Q08188	Protein-glutamine gamma-glutamyltransferase E OS=Homo sapiens OX=9606 GN=TGM3 PE=1 SV=4
P04908	Histone H2A type 1-B/E OS=Homo sapiens OX=9606 GN=H2AC4 PE=1 SV=2
Q92823	Neuronal cell adhesion molecule OS=Homo sapiens OX=9606 GN=NRCAM PE=1 SV=3
P50991	T-complex protein 1 subunit delta OS=Homo sapiens OX=9606 GN=CCT4 PE=1 SV=4
P05543	Thyroxine-binding globulin OS=Homo sapiens OX=9606 GN=SERPINA7 PE=1 SV=2
P04083	Annexin A1 OS=Homo sapiens OX=9606 GN=ANXA1 PE=1 SV=2
P62826	GTP-binding nuclear protein Ran OS=Homo sapiens OX=9606 GN=RAN PE=1 SV=3
P08294	Extracellular superoxide dismutase [Cu-Zn] OS=Homo sapiens OX=9606 GN=SOD3 PE=1 SV=2
P29692	Elongation factor 1-delta OS=Homo sapiens OX=9606 GN=EEF1D PE=1 SV=5
P35555	Fibrillin-1 OS=Homo sapiens OX=9606 GN=FBN1 PE=1 SV=4
P28066	Proteasome subunit alpha type-5 OS=Homo sapiens OX=9606 GN=PSMA5 PE=1 SV=3
Q6UX71	Plexin domain-containing protein 2 OS=Homo sapiens OX=9606 GN=PLXDC2 PE=1 SV=1
P61978	Heterogeneous nuclear ribonucleoprotein K OS=Homo sapiens OX=9606 GN=HNRNPK PE=1 SV=1
P49720	Proteasome subunit beta type-3 OS=Homo sapiens OX=9606 GN=PSMB3 PE=1 SV=2
P02745	Complement C1q subcomponent subunit A OS=Homo sapiens OX=9606 GN=C1QA PE=1 SV=2
O14818	Proteasome subunit alpha type-7 OS=Homo sapiens OX=9606 GN=PSMA7 PE=1 SV=1
Q9BYH1	Seizure 6-like protein OS=Homo sapiens OX=9606 GN=SEZ6L PE=1 SV=1
P30044	Peroxiredoxin-5, mitochondrial OS=Homo sapiens OX=9606 GN=PRDX5 PE=1 SV=4
P60842	Eukaryotic initiation factor 4A-I OS=Homo sapiens OX=9606 GN=EIF4A1 PE=1 SV=1
Q27J81	Inverted formin-2 OS=Homo sapiens OX=9606 GN=INF2 PE=1 SV=2
P01833	Polymeric immunoglobulin receptor OS=Homo sapiens OX=9606 GN=PIGR PE=1 SV=4
P05387	60S acidic ribosomal protein P2 OS=Homo sapiens OX=9606 GN=RPLP2 PE=1 SV=1
P25789	Proteasome subunit alpha type-4 OS=Homo sapiens OX=9606 GN=PSMA4 PE=1 SV=1
P25787	Proteasome subunit alpha type-2 OS=Homo sapiens OX=9606 GN=PSMA2 PE=1 SV=2
P33241	Lymphocyte-specific protein 1 OS=Homo sapiens OX=9606 GN=LSP1 PE=1 SV=1
P11047	Laminin subunit gamma-1 OS=Homo sapiens OX=9606 GN=LAMC1 PE=1 SV=3
P24534	Elongation factor 1-beta OS=Homo sapiens OX=9606 GN=EEF1B2 PE=1 SV=3
P07910	Heterogeneous nuclear ribonucleoproteins C1/C2 OS=Homo sapiens OX=9606 GN=HNRNPC PE=1 SV=4
P14317	Hematopoietic lineage cell-specific protein OS=Homo sapiens OX=9606 GN=HCLS1 PE=1 SV=3
Q9BYJ0	Fibroblast growth factor-binding protein 2 OS=Homo sapiens OX=9606 GN=FGFBP2 PE=1 SV=1
P80188	Neutrophil gelatinase-associated lipocalin OS=Homo sapiens OX=9606 GN=LCN2 PE=1 SV=2
Q8TF66	Leucine-rich repeat-containing protein 15 OS=Homo sapiens OX=9606 GN=LRRC15 PE=2 SV=2

Molecular function clustering using KEGG pathway analysis revealed that isolated EVs were related to different infectious diseases, including African trypanosomiasis, Legionellosis, amoebiasis, and pathogenic *E. coli* (Figure 4C). In addition, analysis for associated diseases demonstrated that the proteins were clustered in retinalassociated diseases such as uveitis, choroidal neovascula-rization, and macular degeneration (Figure 4D).

## DISCUSSION

Classic early symptoms of OT are similar to those of uveitis in general, such as pain, redness, photophobia, and decreased vision^(5)^. Systemic protein biomarkers or biomarkers in the ocular fluids of patients with uveitis may help with diagnosis, prognosis, and treatment. Herein, we aimed to characterize the EV protein cargo isolated from the AH and plasma of patients with OT and compare it with that of patients with cataract (control group).

Several cells secrete EVs under physiological and pathological conditions^(6)^. EVs have been identified in the tears^(15)^, aqueous humor^(10,13)^, vitreous humor^(16)^, and blood^(17)^ of patients with several ocular diseases^(16,17)^. The proteins CD63 and TSG101 are common biochemical constituents in all EV subtypes^(7)^. Recent studies have used CD63 and TSG101 as markers to identify and characterize EVs in patients with lung cancer^(18)^, uveal melanoma^(14)^, chronic Chagas disease^(19)^ and cerebral toxoplasmosis^(20)^. In this study, we analyzed the expression of these EV markers and found that all the plasma samples from patients with OT and CAT were positive for both CD63 and TSG101. Four of the six AH samples from patients with OT were positive only for CD63. However, all the AH samples from patients with CAT were negative for both markers. Thus, the EV marker profiles appear to differ between the plasma and AH, and CD63-positive EVs are more commonly found in the AH of patients with OT than in the AH of the controls. This may be associated with the pathological conditions in the eye caused by *T. gondii*.

Our proteomic analysis detected 803 proteins differentially expressed in the plasma and AH of patients with OT and CAT. Among these, we identified 67 new proteins that have not been previously reported. Of these 67 proteins, seven were only detected in patients with OT, and 10 were detected only in patients with CAT.

In general, the proteins differentially expressed in the AH or plasma samples from patients with OT cover a diverse spectrum of functions associated with inflammatory processes commonly encountered in OT. For instance, the integrin metalloprotease ADAMDEC1 is primarily expressed in myeloid lineage cells and is upregulated by various stimuli and inflammatory states^(21)^. The overexpression of ADAMDEC1 has been described in cutaneous disease, pulmonary sarcoidosis, and systemic lupus erythematosus^(22)^. Fc receptor-like 5 is a novel IgG-binding protein expressed on B cells, and it is involved in the pathogenesis of inflammatory and infectious diseases^(23)^. This protein was described in AH in patients with OT for the first time in this study. However, a study with a larger sample size is required to confirm that the protein can be used as a biomarker.

Among the proteins differentially expressed in the plasma of patients with OT, we identified the Aggrecan core protein (PGCA), a proteoglycan that is the main component of the extracellular matrix of cartilaginous tissue^(24)^. We also found soluble guanylyl cyclase (sGC), a heterodimeric enzyme that is a crucial intracellular target of the signaling molecule nitric oxide (NO)^(25)^. High levels of NO have been identified in human Muller cells and the retinal pigment epithelium in response to cytomegalovirus infection^(26)^. In OT, NO plays a crucial role in protecting against *T. gondii* infection^(27)^.

Another highlighted protein was ArhGAP45, which acts as a Rac-GAP (GTPase-Activating Protein) in endo-thelial cells. It has a negative effect on endothelial barrier function. Silencing ArhGAP45 promotes basal endothelial barrier functions, whereas the loss of ArhGAP45 promotes migration and shear stress adaptation. This suggests that ArhGAP45 is a novel regulator that fine-tunes the regulation of basal endothelial integrity^(28)^. Erbin reportedly plays an important role in cell polarization, receptor localization, and signal transduction^(29)^. It is reportedly involved in the activation of NF-κB and cytokine secretion by interacting with Nod2^(30)^.

Proteomic mining of isolated EVs from the plasma and AH of patients with OT revealed a set of proteins involved in immune system activation, infectious diseases, retina homeostasis, and retina-associated diseases (i.e., uveitis and macular degeneration). These protein profiles warrant further investigation in relation to OT.

The label-free LC-MS used in our study has its limitations that it shares with methods that rely on quantification based on peptide ion peak area measurement. These issues include chromatographic alignment, pepti-de qualification for quantitation, and normalization. Nonetheless, it remains a viable alternative to array-based, gel-based, and stable isotope tag- or label-based approaches. Our analyses reveal that comprehensive, accurate, and reproducible protein identification and quantification are achievable between all sample groups.

In conclusion, EV-derived proteins from the plasma and/ or AH of patients with OT could be used as bio-markers in the future. The role of these proteins in the pathogenesis of OT needs further investigation, which may lead to the development of new treatment and diagnostic strategies.
